# Aberrant phase separation from a rare *ABI3* mutation drives microglial dysfunction and Alzheimer's risk

**DOI:** 10.1002/alz.71812

**Published:** 2026-09-24

**Authors:** Shengnan Li, Jiongtong Lu, Kaixing Zeng, Xingyu Lu, Jiahao Zhang, Sifan Feng, Chunmei Liang, Yujie Cai, Shi Yao, Qing Huang, Guocong Liang, Rui Chen, Li Li, Fubin Ma, Jiawei Xing, Chaoyue Ouyang, Lv Luo, Jiaying Wang, You Li, Shengbo He, Yuanyuan Zhao, Yan Wang, Lili Cui

**Affiliations:** ^1^ Institute of Neurology, Guangdong Key Laboratory of Age‐Related Cardiac and Cerebral Diseases Affiliated Hospital of Guangdong Medical University Zhanjiang China; ^2^ The Marine Biomedical Research Institute of Guangdong, School of Ocean and Tropical Medicine Guangdong Medical University Zhanjiang China; ^3^ Guangdong Laboratory for Lingnan Modern Agriculture, State Key Laboratory for Conservation and Utilization of Subtropical Agro‐Bioresources, Guangdong Provincial Key Laboratory of Plant Molecular Breeding South China Agricultural University Guangzhou China; ^4^ Institute of Neuroscience, Translational Medicine Institute Xi'an Jiaotong University Health Science Center Xi'an Shaanxi China; ^5^ Department of Human Anatomy, Histology and Embryology, School of Basic Medical Sciences Xi'an Jiaotong University Xi'an Shaanxi China

**Keywords:** Abelson‐interactor family member 3, Alzheimer's disease, amyloid beta, liquid–liquid phase separation, microglia

## Abstract

**INTRODUCTION:**

Although the *ABI3 S209F* variant is a recognized genetic risk for Alzheimer's disease (AD), its pathogenic mechanism remains elusive.

**METHODS:**

Using AD mouse models (amyloid precursor protein/presenilin 1 [APP/PS1] transgenic mice, 5×familial Alzheimer's disease (5×FAD) transgenic mice) with microglia‐specific knockdown of Abelson‐interactor family member 3 (*ABI3)* or expression of the wild‐type or *S209F* mutant, we assessed disease pathology. Primary mouse microglia and HMC3 cells were used to examine ABI3 function and *S209F* effects. Liquid–liquid phase separation (LLPS) properties were characterized in cellular systems and with recombinant proteins.

**RESULTS:**

ABI3 was highly expressed in AD microglia. ABI3 enhanced microglial clustering around amyloid beta (Aβ) plaques, promoted Aβ clearance, and ameliorated cognitive decline, whereas *S209F* abolished these effects. Mechanistically, ABI3 undergoes LLPS essential for microglial migration and phagocytosis. The S209 residue lies within an intrinsic disordered region, and *S209F* disrupts phosphorylation‐dependent LLPS, thereby impairing microglial functions.

**DISCUSSION:**

The AD‐associated *ABI3 S209F* variant drives pathogenesis by disrupting LLPS, causing microglial dysfunction. Enhancing ABI3 phase separation represents a potential therapeutic strategy to boost microglial activity against Aβ pathology.

## Highlights

- The ability of Abelson‐interactor (ABI) family member 3 (ABI3) to mitigate amyloid pathology and rescue memory deficits is critically dependent on the S209 site, as the *S209F* mutation completely abolishes these neuroprotective functions.
- The *ABI3 S209F* variant impairs its capacity for liquid–liquid phase separation (LLPS), as demonstrated in both live cells and in vitro reconstitution systems.
- LLPS of ABI3 is cooperatively regulated by phosphorylation at Ser209 and its intrinsic disordered region (IDR).
- The LLPS defect and microglial dysfunction caused by the *ABI3 S209F* mutation are rescued by the FUS RNA binding protein (FUS) IDR fusion.
- Loss‐of‐function (1,6‐hexanediol) and gain‐of‐function (optogenetic) experiments establish a causal link between impaired LLPS and *ABI3 S209F*‐mediated microglial dysfunction.

## 1 BACKGROUND

Alzheimer's disease (AD) is the most common age‐related neurodegenerative disorder, affecting tens of millions of individuals worldwide and placing an immense burden on public health and society.^1^ Despite decades of intensive study, the mechanisms driving AD pathogenesis remain incompletely understood, limiting therapeutic progress and underscoring the need to uncover novel molecular pathways.^2^, ^3^ Although mutations in amyloid precursor protein (*APP*) and presenilins (*PSEN1*, *PSEN2*) account for only a small proportion of early‐onset familial AD cases,^4^, ^5^ genome‐wide association studies (GWASs) have provided accumulating evidence that gene polymorphisms increase the risk of the more common late‐onset AD (LOAD).^6^, ^7^ Microglia are the resident macrophages of the central nervous system that exert protective function by phagocytosing and clearing pathological protein aggregates.^8^ Many LOAD risk‐associated genes, such as Apolipoprotein E4 (*APOE4)*,^9^, ^10^ Triggering receptor expressed on myeloid cells 2 (*TREM2)*,^11^ and Phosphatidylinositol‑binding clathrin assembly protein (*PICALM)*,^12^ have been implicated in regulating microglial immunity and lipid metabolism,^13^ supporting an important role of microglial biology in AD susceptibility.

Abelson‐interactor (ABI) family member 3 (ABI3) is a component of the WASP‑family verprolin‑homologous protein 2 (WAVE2 regulatory complex (WRC2) and has been implicated in regulating actin cytoskeleton dynamics.^14^A coding variant in *ABI3* (rs616338; p. Ser209Phe, *ABI3 S209F*) was first identified in a GWAS of more than 85,000 individuals as a risk allele for LOAD,^15^ with subsequent studies confirming its association.^16^ Functional studies suggest that loss of ABI3 exacerbates AD pathology through aberrant microglial responses,^17^ and the *S209F* mutation has been proposed to alter ABI3 phosphorylation and impair its function.^18^ Smith et al. demonstrated that deletion of *Abi3* (*Abi3* ^−/−^) leads to significant metabolic dysfunction, highlighting a broader physiological role for ABI3 in metabolic regulation.^19^ Recent studies have found that the *ABI3 S209F* variant is associated with an increased risk and earlier onset of AD, and when combined with apolipoprotein E (*APOE*) ε4, it can promote region‑specific Aβ accumulation and microglial activation.^20^ Despite these strong genetic and biological links, the molecular mechanism by which *ABI3 S209F* contributes to AD remains unknown.

Biomolecular condensates formed through liquid–liquid phase separation (LLPS) have emerged as critical organizers of cellular biochemistry. LLPS is often driven by proteins containing intrinsically disordered regions (IDRs), which facilitate multivalent interactions and dynamic condensate assembly.^21^ By dynamically compartmentalizing proteins and nucleic acids without membranes, LLPS regulates diverse processes in health and disease.^22^, ^23^ Aberrant LLPS, or its transition to liquid–solid states, promotes pathological protein aggregation in neurodegenerative disorders.^24^ In AD, tau's mutations or abnormal post‐translational modifications (PTMs) induce aberrant and excess LLPS, driving fibril formation and neurotoxicity.^25^ Notably, many proteins involved in actin cytoskeletal dynamics undergo LLPS to concentrate effectors and facilitate signal transduction.^26^ ABI3 is an important regulator of actin assembly^27^; however, whether it undergoes LLPS remains unknown. The AD‐associated *S209F* mutation, located adjacent to a critical phosphorylation site (Ser209) that regulates ABI3 function, is a key PTM that increases the risk of AD.^18^ Yet, whether the *S209F* mutation undergoes LLPS and contributes to neurodegeneration through mechanisms distinct from aggregation remains largely unexplored.^28^

Here, we found that both ABI3 deficiency and the *S209F* mutation disrupt normal microglial migration, phagocytosis, and clustering around Aβ plaques. ABI3 participates in LLPS both in vitro and in vivo. The *S209F* mutation appears to impair Ser209 phosphorylation and the IDR of ABI3, thereby diminishing the LLPS capacity of ABI3, leading to amyloid pathology and defective cognitive functions in AD models. These findings identify ABI3 as a previously unrecognized phase‐separating protein for neuroprotection and reveal a non‐aggregative LLPS mechanism linking genetic variation to impaired microglial function and AD pathogenesis.

**RESEARCH IN CONTEXT**

1. **Systematic review**: Genome‐wide association studies have identified the rare *ABI3 S209F* missense variant as a genetic risk factor for Alzheimer's disease (AD). However, the molecular mechanism by which this variant influences microglial activity and promotes AD pathogenesis has remained unknown.
2. **Interpretation**: We demonstrate that the *S209F* variant disrupts liquid–liquid phase separation (LLPS) of ABI3, impairing its ability to form cytosolic condensates. This defect compromises key microglial functions, including migration, phagocytosis, and clustering around amyloid beta (Aβ) plaques. Consequently, *ABI3 S209F* leads to increased amyloid burden and accelerated cognitive decline in 5×FAD mice, whereas microglial expression of wild‐type ABI3 rescues these phenotypes. Mechanistically, we establish that LLPS of ABI3 is functionally required for efficient microglial migration and phagocytosis. Our findings reveal a causal link between ABI3 LLPS deficiency, microglial dysfunction, and elevated AD risk.
3. **Future directions**: Future studies will focus on identifying ABI3‐interacting proteins and the molecular drivers of its LLPS behavior. We aim to develop small‐molecule strategies that enhance ABI3 phase separation as a potential therapeutic approach to restore microglial function in AD.

## 2 METHODS

### 2.1 Animals

All animal experiments described in this article were approved by the Ethics Committee for Experimental Animals of the Affiliated Hospital of Guangdong Medical University (License number: SYXK (Guangdong) 2022‐0286). 5×FAD male mice [B6SJL‐Tg (APPSwFlLon, PSEN1*M146L*L286V)6799Vas/Mmjax] were purchased from Cavins Laboratory Animal Co., Ltd., of Changzhou (Changzhou, China), and APP/PS1 male mice [C57BL/6(APPswe/PSEN1dE9)] were purchased from Hangzhou Ziyuan Laboratory Animal Technology Co., Ltd. All procedures followed National Institutes of Health (NIH) guidelines and were approved by the Affiliated Hospital of Guangdong Medical University Animal Care Committee.

For *ABI3* knockdown studies, we utilized APP/PS1 mice (adeno‑associated virus [AAV] injections at 6 months, tissue collection at 12 months). For overexpression studies of ABI3 wild‐type (WT) and *ABI3 S209F*, we employed 5×FAD mice (AAV injections at 6 months, behavioral testing at 8 months, and tissue collection at 9 months).

### 2.2 Cell culture

The human embryonic kidney (HEK)293T, human microglial cell line 3 (HMC3), mouse microglial BV2, and SH‐SY5Y human neuroblastoma cells were purchased from Procell Life Science & Technology Co., Ltd. HEK 293T cells and BV2 were maintained in Dulbecco's modified Eagle's medium (DMEM) supplemented with 10% fetal bovine serum (FBS) and 1% penicillin/streptomycin, and the HMC3 and SH‐SY5Y cells were maintained in minimum essential medium (MEM) supplemented with non‑essential amino acids (NEAA).

### 2.3 Plasmid construction and transfection

Full‐length ABI3 (NP_057512.1), *ABI3 S209F* (NP_057512.2), *ABI3‐S209A*, *ABI3‐S209D*, and different truncated variants, including ABI3‐∆IDR1 (89‐132), ABI3‐∆IDR2 (161‐303), ABI3‐∆SH3 domain, ABI3‐∆Abi domain, *ABI3 S209F*‐∆IDR3 (161‐200), *ABI3 S209F*‐∆IDR4 (207‐303), *ABI3 S209F*‐FUS IDR, ABI3‐ΔSH3 domain‐FUS IDR, ABI3 ΔIDR2‐FUS IDR, *ABI3 S209F*‐∆IDR4‐FUS IDR, and *ABI3 S209F*‐ABI3 IDR2 were amplified and subcloned into the pCMVPuro‐EGFP vector. ABI3 IDR[89‐132] was fused to the Cry2 protein to construct the recombinant plasmids optoABI3 IDR[89‐132]‐mCherry‐Cry2. One microgram of plasmid was transfected with Lipofectamine 2000 (Thermo Fisher) into HEK293T or SH‐SY5Y cells. Two micrograms of plasmid was transfected with Lipofectamine 3000 into HMC3 or BV2 cells. One microgram of plasmid was transfected with Lipofectamine 3000 into primary microglia.

### 2.4 IDR prediction

The IDRs of human full‐length ABI3 (NP_057512.1) and *ABI3 S209F* (NP_057512.2) [366 amino acids (aa)] were predicted using PONDR.

### 2.5 Recombinant ABI3 and *ABI3 S209F* protein expression and purification

The target DNA sequences of ABI3 and *ABI3 S209F* were optimized, synthesized, and cloned into the pET28a‐maltose‑binding protein (MBP) vector with a histidine‑tag (His tag), and then tagged with green fluorescent protein (GFP). The recombinant plasmids were transformed into Escherichia coli (*E* *. coli*) BL21 (DE3). After inoculating individual colonies in antibiotic‐supplemented Luria‑Bertani (LB) medium and incubating at 37°C with 200 rpm, IPTG was used to induce protein expression. When optical density at 600 nm (OD600) reached about 1.2, further induction was done at 15°C for 16 h. Cells were then processed for purification, using an MBP column, Tobacco Etch Virus (TEV) protease cleavage, an nickel‑affinity (Ni) column, and a Superdex 20 column. The target protein was sterilized, its concentration determined by Bradford assay, and its purity/molecular weight by sodium dodecyl sulfate‑polyacrylamide gel electrophoresis (SDS‐PAGE). The purified proteins were stored in a specific buffer.

### 2.6 Formation of ABI3 and *ABI3 S209F* condensates in the presence of different concentrations of crowding agents and salts

Purified ABI3‐GFP and *ABI3 S209F*‐GFP were diluted. Polyethylene glycol 8000 (PEG 8000), Ficoll 400, and NaCl were sterilized and diluted as described previously.^29^ Proteins were mixed with them, incubated, and observed under a confocal microscope. The size and number of condensates were measured by ImageJ software.

### 2.7 Analysis of ABI3 and *ABI3 S209F* protein condensate formation induced by 1,6‐hexanediol (1,6‐HD) in vitro and in cells

To assess the dependence of ABI3 condensates on liquid–liquid phase separation, cultured cells were treated with 1,6‐hexanediol (1,6‐HD; Sigma‐Aldrich, #240117). A 5% (v/v) stock solution was prepared fresh in culture medium and diluted to a final concentration of 2.5% immediately before use as described previously.^30^ ABI3 and *ABI3 S209F* recombinant proteins of specified concentration were mixed with 5% 1,6‐HD and placed in a confocal dish for 5 min in vitro before being observed.

To assess cell viability, CCK‐8 assays (Dojindo, #CK04) were performed strictly according to the manufacturer's standard protocol. HEK 293T cells were treated with 2.5% or 5% 1,6‐HD for 2.5, 5, 7.5 or 10 min, and absorbance at 450 nm was accurately measured using a professional microplate reader. Cell viability was calculated relative to matched untreated control groups. For experiments disrupting intracellular phase‐separated condensates, after 24‐h transfection in HEK 293T cells, 2.5% or 5% 1,6‐HD was added and incubated at 37°C for 5 min before observation by confocal microscope. For functional assays (migration and phagocytosis), HMC3 and BV2 cells were treated with 2.5% or 5% 1,6‐HD for 5 min prior to the assay.

### 2.8 Measurement of the turbidity of ABI3 and *ABI3 S209F* during LLPS in vitro

Recombinant ABI3 or *ABI3 S209F* was mixed with 10% PEG 8000, added to a 96‐well plate. OD600 was measured by an enzyme‐labeling instrument to assess solution turbidity.

### 2.9 Fluorescence recovery after photobleaching (FRAP) assay in vitro

Recombinant ABI3 and *ABI3 S209F* were combined with 10% PEG 8000. Fifty microliters of each mixture was incubated at room temperature for 5 min and placed in a glass‐bottomed confocal dish. An Olympus laser confocal microscope was used to observe condensates and conduct Fluorescence Recovery After Photobleaching (FRAP) tests. A 488 nm laser bleached the condensates at full intensity for 3 s, and images were captured every 20 s for 140 s post‐bleaching. ImageJ processed the pictures, normalizing region of interest (ROI) fluorescence. The FRAP experiment was repeated five times. GraphPad Prism 10.1.2 analyzed the data and plotted FRAP curves, while FIJI ImageJ measured droplet features. Consistent microscope settings ensured reliable results, and a 360‐4095 gray threshold removed background noise.

### 2.10 Analysis of ABI3 and *ABI3 S209F* protein condensate formation in HEK293T or HMC3 cells

HEK 293T or HMC3 cells were cultured on poly‑D‑lysine (PDL) (50 µg/mL)–coated dishes. The pCMVPuro‐EGFP, pCMVPuro‐EGFP‐ABI3, and pCMVPuro‐EGFP‐*ABI3 S209F* plasmids were transfected into the cells when the cell density reached ≈50%. After fixation and DAPI staining, condensates were visualized, and their number, diameter, and area were analyzed by FIJI ImageJ.

### 2.11 FRAP assay in HEK293T cells

HEK 293T or HMC3 cells on PDL (50 µg/mL)–coated dishes were transfected with ABI3 or *ABI3 S209F* plasmids at about 50% confluence. Twenty‐four hours later, live‐cell FRAP was recorded using a 60× oil lens and an Olympus confocal microscope. A 488 nm laser bleached protein condensates at full intensity for 5 s. Images were acquired every 10 s for a total of 200 s post‑bleaching. The experiment was repeated five times, and GraphPad Prism 10.1.2 was used to analyze the data and plot FRAP curves.

### 2.12 Monitoring of droplet fusion and fission by live‐cell imaging

Glass‐bottomed confocal dishes (35 mm) were coated with PDL (50 µg/mL) overnight and washed. HEK 293T or HMC3 cells were transfected at about 50% confluence. ABI3 and *ABI3 S209F* droplet fusion and fission were observed in real time for 15 min.

### 2.13 Light‐activated optoDroplets

HEK 293T cells were cultured overnight in 35 mm PDL‐coated glass‐bottomed dishes until 50% confluent and then transfected with mCherry‐Cry2 or ABI3 IDR [89‐132]—mCherry‐Cry2 plasmids. Cells with similar fluorescence intensities were activated with a 488 nm blue laser, while mCherry signals were imaged with a 561 nm laser every 15 s for 10 min to record phase separation dynamics, as described previously.^31^ For the optoDroplet‐FRAP, ABI3 biomolecular aggregates were photobleached for 5 s with a 561 nm laser at an 80% laser intensity and then photographed every 15 s.

For the optoDroplet‐cell migration and phagocytosis assays, HMC3 and BV2 cells were transfected with either opto‐ABI3 (ABI3‐IDR‐mCherry‐Cry2) or the control opto‐mCherry (mCherry‐Cry2) plasmid. Twenty‐four hours post‐transfection, cells were subjected to 6 h of continuous blue light (488 nm) to induce optogenetic activation. Immediately after light stimulation, cell migration and phagocytosis assays were carried out. The data were analyzed by GraphPad Prism 10.1.2.

### 2.14 Cell migration

HMC3 cells, BV2, or primary microglia were transduced with ABI3‐EGFP or *ABI3 S209F*‐EGFP lentivirus and seeded in a 6‐well plate. The next day, cells were scratched using a 200 µL pipette tip. After washing with phosphate‐buffered saline (PBS), MEM with 1% serum (including 100 nM Aβ) was added. Images were captured at 0, 6, 12, and 24 h. Image‐Pro Plus measured wound closure and relative wound density to evaluate cell migration.^17^

### 2.15 Cell phagocytosis

The number of red fluorescent latex beads phagocytosed by HMC3, BV2, or primary microglia was assessed to determine the uptake of the beads by the cells as described previously.^32^ Cells were grouped as pCMVPuro‐ABI3‐EGFP, pCMVPuro‐*ABI3S209F*‐EGFP, and pCMVPuro‐EGFP. Cells (2×10^5^) were seeded on PDL‐coated 35 mm dishes, cultured for 24 h, and then transfected. Another 24 h later, medium with fluorescent latex beads (1:10,000) was added. After 3‐h incubation, cells were collected, washed, fixed with 4% paraformaldehyde (PFA), stained with DAPI, and observed under a confocal microscope to count the phagocytosed beads.

### 2.16 AAV construct

*ABI3* (Iba1p‐MCS‐EGFP‐3Flag‐SV40 Poly A *ABI3* AAV) and *ABI3 S209F* (Iba1p‐MCS‐EGFP‐3Flag‐SV40 Poly A *ABI3* *S209F*) were constructed. AAV‐Control‐EGFP, AAV‐ABI3‐EGFP, and AAV‐ABI3 S209F‐EGFP were injected into the hippocampus of 6‐month‐old 5×FAD mice via a mouse brain stereotaxic instrument. For microglia‐specific knockdown of *ABI3*, we employed an AAV vector expressing an artificial miRNA under the control of the microglial Iba1 promoter. The vector backbone (Iba1p‐EGFP‐miR155 (mcs)‐SV40 PolyA) was custom designed and packaged by Shanghai Genechem Co., Ltd. (Shanghai, China). The IBA1 promoter used in this study has been validated extensively for microglial specificity, with reported transduction specificity of up to 95% in resident microglia.^33^, ^34^, ^35^ This promoter, paired with an artificial miRNA scaffold embedded in the miR‐RNA backbone, is a well‐established strategy for microglia‐specific gene manipulation.^33^, ^34^, ^35^ This vector uses the microglia‐specific promoter to drive expression of both EGFP (for visualization of transduced cells) and an miR155‐based artificial miRNA targeting *Abi3*. The shRNA sequences targeting mouse *Abi3* were: sense: 5'‐ACCGCTAGCTAACTGGAGGCTTGCTGAAGGCTGTATGCTGTGTAGCAGGCGCCTTTATGCTGTTTTGGCCACTGACTGACAGCATAAACGCCTGCTACACAGGACACAAGGCCTGTTACTAGCACTCACATGGAACAAATGGCCCAAGCTTGGT‐3'; antisense:5'‐ACCAAGCTTGGGCCATTTGTTCCATGTGAGTGCTAGTAACAGGCCTTGTGTCCTGTGTAGCAGGCGTTTATGCTGTCAGTCAGTGGCCAAAACAGCATAAAGGCGCCTGCTACACAGCATACAGCCTTCAGCAAGCCTCCAGTTAGCTAGCGGT‐3'. A scrambled non‐targeting sequence was used as control. AAV6 serotype was used for enhanced microglial tropism. Viruses were produced by Shanghai Genechem Co., Ltd. at a titer of 5.52 × 10^1^ ^2^ TU/mL.

### 2.17 Stereotactic injection of AAV into the hippocampus

The mice (6‐month‐old APP/PS1 or 5×FAD) were anesthetized with 1.25% 2,2,2‐tribromoethanol (200 µL/10 g, intraperitoneal [i.p.]) and fixed in a stereotaxic instrument. After disinfection, the scalp was cut and a 1‐mm hole was drilled in the skull. Based on the mouse brain stereotaxic map, specific coordinates were used for left and right hippocampus injection. A total of 1 µL of AAV suspension was injected bilaterally into the hippocampus at the following coordinates relative to bregma: anteroposterior (AP) –2.0 mm, mediolateral (ML) ± 1.5 mm, dorsoventral (DV) –1.8 mm. AAV (titer ∼1 × 10^1^ ^2^ TU) was slowly injected at 0.1 µL/min, taking 10 min. The needle stayed for 5 min before withdrawal, and hyaluronic acid gel was applied to the wound for healing. After injection, the scalp was sutured. APP/PS1 mice were allowed to recover for 6 months to ensure maximal transgene expression and gene knockdown effects, and were euthanized at 12 months of age to obtain tissue samples. For 5×FAD mice, behavioral experiments were conducted at 8 months of age to assess cognitive function, and they were euthanized at 9 months of age to obtain tissue samples.

### 2.18 Open field test

Mice were put in a 40 × 40 × 40 cm light‐proof square box, and their movements were video‐recorded for 10 min. Behavior analysis software and GraphPad Prism 10.1.2 analyzed parameters like speed, total distance, central‐area speed, distance, time, and time percentage.

### 2.19 Novel object recognition test

The novel object recognition test was carried out in a 40 × 40 × 40 cm opaque square box. The experiment had three phases, each 2 h apart: adaptation, training, and test. In the adaptation phase, with no objects in the box, mice moved freely for 10 min. In the training phase, two 5 × 5 × 5 cm wooden blocks of the same color and shape were placed 10 cm from the sidewalls. Mice in the box center were video‐recorded for 10 min while exploring the blocks. In the test phase, one block was replaced with a 5 cm diameter, 5 cm high cylinder. Mice were put between the two objects and explored freely for 8 min. Behavioral analysis software evaluated the exploration time. The recognition index (RI) for each group was calculated as: (time on new object / (time on new object + time on old object))×100%.

### 2.20 Y‐maze test

The Y‐maze test was conducted in a Y‐shaped maze (25 cm long, 8 cm wide, 40 cm deep) with three arms at 120° angles. It consisted of two parts: a working memory test and a spatial memory test. In the working memory phase, mice were placed randomly in one arm and freely explored all three arms. Consecutive entries into different arms were recorded, and the number of spontaneous alternations was counted to calculate the maximum number of alternations (total entries‐ 2) and the spontaneous alternation rate (number of alternations / maximum number) × 100%. For spatial memory, different colored and shaped stickers were placed at the ends as cues. This part had two sub‐phases, 2 h apart. In Part 1, one arm was closed, and mice explored two open arms for 10 min. In Part 2, all arms were open, and mice explored for 5 min. The percentage of entries into the familiar arm was calculated as the number of entries into the familiar arm/(the number of entries into the familiar arm + the number of entries into the novel arm), and the percentage of entries into the novel arm was calculated as the number of entries into the novel arm/(the number of entries into the familiar arm + the number of entries into the novel arm).

### 2.21 Morris water maze test

The Morris water maze test was carried out in a 120 cm diameter circular pool filled with 40 cm deep water. Titanium dioxide was added to the water, and its temperature was maintained at 25°C. The room walls had distinct cues for spatial recognition. The pool was divided into four equal quadrants. During the 1‐ to 6‐day training, an 8 cm diameter, 30 cm high escape platform was set in the southwest (target) quadrant. It was 1–2 cm above water on Day 1 and 0.5 cm submerged from Days 2–6. Daily, mice were placed into the maze from four directions. The time they took to find the platform within 90 s, and their movement trajectories were recorded. If a mouse failed to find the platform in 90 s, it was guided and left on the platform for 20 s. On Day 7, the platform was removed for the probe test. Mice were placed in the northeast quadrant, and relevant data were recorded. Morris water maze software analyzed swimming speed, time in the target quadrant, platform‐crossing number, and other parameters for each group of mice.

### 2.22 Immunofluorescence staining of frozen tissues

Mice were anesthetized with 1.25% 2,2,2‐tribromoethanol (200 µL/10 g, i.p.) and sacrificed by transcardial perfusion. After being fixed on foam boards, their thoraxes were dissected. A perfusion needle is inserted into the left ventricle, the right atrial appendage is cut, and 4% PFA is injected until the liver turned pale and limbs became rigid. Brain tissues were removed, washed, soaked in 4% PFA overnight, dehydrated in sucrose, embedded, sliced, and incubated at –80°C. The 20‐µm slices were treated and then incubated with primary antibodies against anti‐ABI3 (Biorbyt, Orb221333‐100, 1:50), anti‐Iba1 (Wako, 019‐19741, 1:200), and anti‐Aβ (Abcam, ab11132, 1:200) at 4°C for over 18 h. The next day, sections were washed, incubated with secondary antibodies in the dark, washed again, and sealed. The morphological changes in microglia in the brain were evaluated, and the number of ABI3‐positive microglia and Aβ‐positive microglia in brain tissues from the different groups was evaluated by Image‐Pro‐Plus 6.0.

For cell type–specific ABI3 quantification, brain sections from four mice per group were analyzed. For each mouse, 10 hippocampal fields (CA1, CA3, dentate gyrus) were imaged. ABI3 mean fluorescence intensity was quantified in Iba1^+^ (microglia), NeuN^+^ (neurons), and GFAP^+^ (astrocytes) cells with DAPI^+^ nuclei by two blinded observers.

For all in vivo experiments, the mouse served as the biological replicate. For each mouse, 5–6 coronal sections (20 µm) spanning the hippocampus were selected at regular intervals (every 240 µm). Within each section, 3–5 fields were acquired in hippocampal subregions. Values were averaged across all fields and sections to generate a single per‐mouse average, which was used for statistical comparisons. Individual cells or fields were not treated as independent replicates. Sample sizes (*n*) represent the number of mice per group and are indicated in figure legends.

To assess cell‐type specificity of AAV‐mediated knockdown, brain sections were co‐stained with antibodies against ABI3 and cell type–specific markers: Iba1 for microglia, NeuN for neurons, and GFAP for astrocytes. Knockdown efficiency and cell‐type specificity were evaluated by quantifying ABI3 fluorescence intensity within each marker‐positive cell population.

### 2.23 Western blotting

Cold PBS was prepared in advance, and the mice were anesthetized with 1.25% 2,2,2‐tribromoethanol (200 µL/10 g, i.p.). Their limbs were fixed on a foam board, and the chest was opened to expose the heart. A needle was inserted at the heart apex, and sterilized saline was injected to clear blood. Then the hippocampus and cortex were quickly removed and placed in a 2 mL tube with radio‑immunoprecipitation assay (RIPA) lysis buffer supplemented with protease and phosphatase inhibitor cocktail. After homogenization and 20‐min ice incubation, the sample was centrifuged. The supernatant was collected, and protein concentration was quantified by bicinchoninic acid (BCA) protein‑quantification assay for western blotting. Membranes were incubated with primary antibodies: anti‐ABI3 (Abcam, ab214318, 1:1000) and anti‐GAPDH (Cell Signaling Technology, 5174, 1:5000) at 4°C overnight, and secondary antibody at room temperature for 1 h to analyze target protein expression.

### 2.24 Reverse‑transcription quantitative polymerase chain reaction (RT‐qPCR)

The mouse hippocampus was rapidly stripped and placed in 1 mL of TRIzol® reagent in a grinder to extract RNA, which was then reverse‐transcribed into cDNA. The reverse transcription reaction system was set up in a 0.2 mL PCR tube. After denaturation and cooling, a reagent mixture was added for cDNA synthesis. The reverse‑transcription quantitative polymerase chain reaction (RT‐qPCR) system was prepared with specific components. The qPCR conditions included an initial 95°C for 10 min, followed by 40 cycles of 95°C for 15 s and 60°C for 1 min. The primer sequences for Mus‐ *Abi3* and Mus‐*Gapdh* are provided for the experiment.

*Abi3*: F5′‐CTACTGCGAGGATAACTACTTGC‐3′,R5′‐CAGGTTACCCACTTGGTAGGC‐3′,*Gapdh*: F5′‐AAGAGGGATGCTGCCCTTAC‐3′,R5′‐TACGGCCAAATCCGTTCACA‐3′.

### 2.25 Primary microglia isolation and culture

Primary microglia were isolated from postnatal days 1–3 C57BL/6J mouse pups as described previously with modifications.^36^ Briefly, whole brains were removed, stripped of meninges, and mechanically dissociated. The cell suspension was passed through a 70 µm cell strainer and cultured in Dulbecco’s Modified Eagle Medium/Nutrient Mixture F‑12 (DMEM/F12) supplemented with 10% fetal bovine serum (FBS) and 1% penicillin/streptomycin in T75 flasks pre‐coated with poly‐d‐lysine (50 µg/mL). After 10–14 days, microglia were detached by orbital shaking on an orbital shaker (200 rpm for 2 h at 37°C), collected, and re‐plated for experiments. Purity was assessed by immunostaining for Iba1 and was consistently >95%.

For lentivirus transduction, primary microglia were seeded in 24‐well plates at 1×10^5^ cells/well and transduced with lentivirus (multiplicity of infection [MOI] = 1×10^5^ v.g./cell) for 72 h prior to functional assays.

### 2.26 Single‐cell RNA‐seq and spatial transcriptomics in APP/PS1 mice

scRNA‐seq and spatial transcriptomics were performed as described previously.^37^, ^38^ Brain tissues were dissected from 8‐month‐old APP/PS1 transgenic mice. Single‐cell suspensions were prepared via mechanical and enzymatic dissociation (papain/DNase I) in oxygenated DMEM, followed by Percoll gradient centrifugation to remove debris and FACS to enrich viable cells, and then loaded onto a 10×Genomics Chromium Controller. scRNA‐seq libraries were constructed with 10×Genomics Chromium Next GEM Single Cell 3' Kit and sequenced on Illumina NovaSeq for paired‐end reads. Raw data were processed via Cell Ranger (v7.0, aligned to mm10 reference genome) and analyzed with Seurat in R: low‐quality cells/doublets were filtered, followed by PCA, Louvain clustering, and UMAP visualization. Cell types were annotated using canonical markers (*Cx3cr1/Ctss* for microglia, *Gfap* for astrocytes, *Mbp* for oligodendrocytes, *Rbfox3* for neurons) and validated with SingleR; microglia were subset for re‐clustering. For spatial transcriptomics, 10 µm coronal brain sections from APP/PS1 mice were placed on Visium slides, fixed, hematoxylin and eosin (H&E) stained, imaged, and then permeabilized for reverse transcription and library construction. Data processing and spatial alignment were performed via Space Ranger.

### 2.27 Statistical analysis

For all in vivo quantitative analyses, individual mice were used as the independent biological replicates (*n* = number of mice per group). For each mouse, measurements were obtained from multiple tissue sections (5–6 sections per animal) and multiple fields of view (3–5 fields per section). These values were first averaged within each animal to generate a single per‐mouse value, which was then used for all between‐group statistical comparisons.

For in vitro cell‐based assays, cytoplasmic droplet aggregates were quantified in at least 300 cells per experiment across at least three independent biological replicates. Values from 3–5 fields of view were averaged per cell, and these per‐cell averages were compared between experimental groups. For recombinant protein liquid–liquid phase separation (LLPS) assays, data were derived from at least three independent experiments, with the mean value from 3–5 fields of view used as a single data point per protein sample group.

Data are presented as mean ± standard error of the mean (SEM) using GraphPad Prism 10.1.2. Normality was assessed using Shapiro–Wilk tests. For multiple comparisons, one‐way analysis of variance (ANOVA) with Tukey's post hoc test was applied. Two‐group comparisons used Student's *t*‐test (equal variance) and Welch's *t*‐test (unequal variance).

## 3 RESULTS

### 3.1 ABI3 is preferentially expressed in microglia and up‑regulated in AD mouse models

**FIGURE 1:**
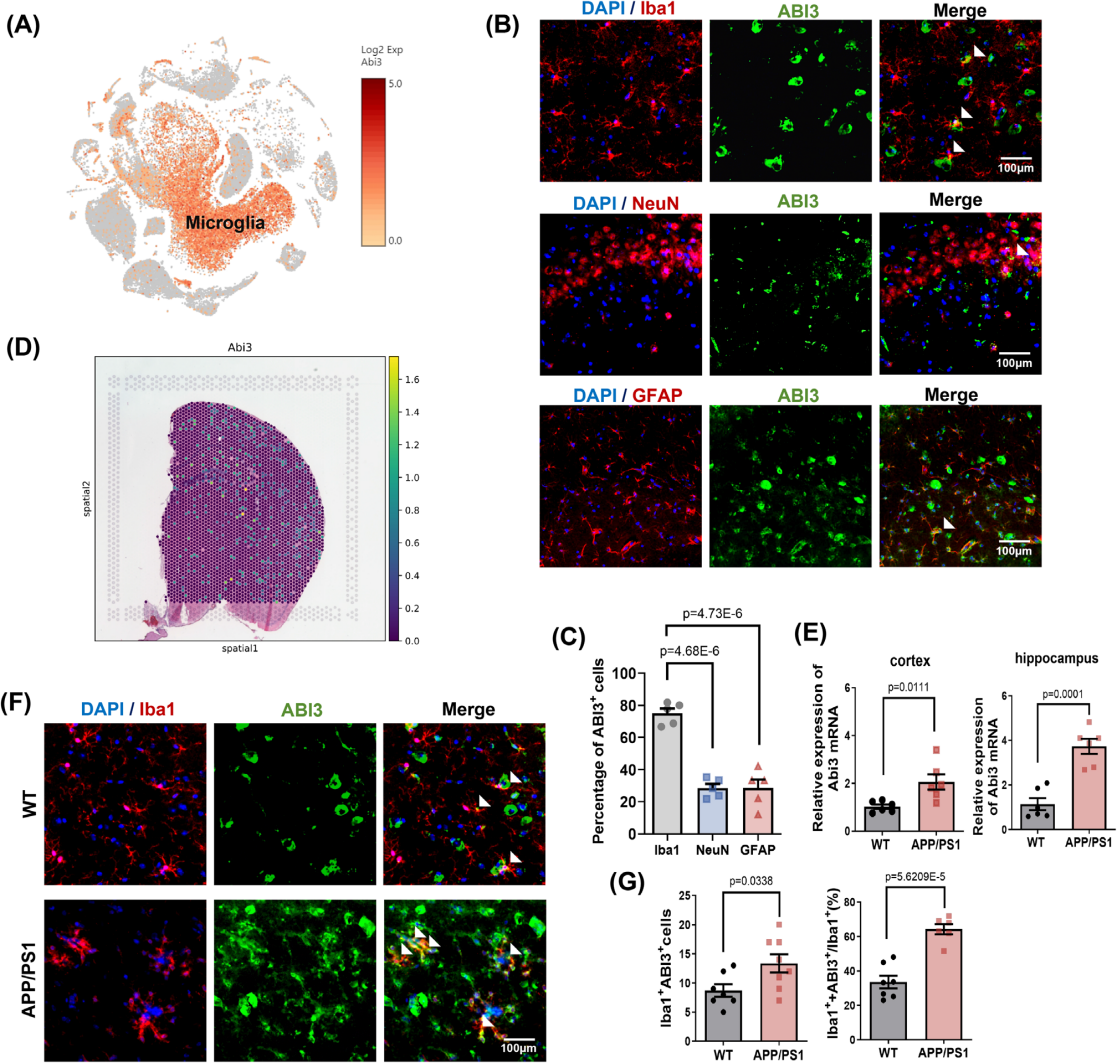
Microglial‐specific upregulation of ABI3 in AD mice. (A) single‑cell RNA‑sequencing (scRNA‐seq) reveals ABI3‐specific expression in microglia. (B) ABI3 co‐localizes with microglia (ionized calcium‑binding adapter molecule 1^+^[Iba1^+^]), neurons (neuronal nuclei^+^[NeuN^+^]), and astrocytes (glial fibrillary acidic protein^+^ [GFAP^+^]) in 8‐month‐old C57BL/6 mice. White arrows denote Iba1^+^ABI3^+^, NeuN^+^ABI3^+^, and GFAP^+^ABI3^+^ cells. Scale bar: 100 µm. (C) Statistical plots revealed that ABI3 is predominantly highly expressed in microglia (*n* = 5/group). (D) Spatial transcriptomics reveals that *Abi3* is expressed in the cerebral cortex and hippocampus. (E) APP/PS1 transgenic (Tg) mice showed higher *Abi3* mRNA levels in the cortex (left) and hippocampus (right) versus C57BL/6 mice (*n* = 6/group). (F) Representative images of ABI3 expression in hippocampal microglia of 8‐month‐old C57BL/6 mice, with enhanced expression in APP/PS1 Tg mice. White arrows indicate Iba1^+^ABI3^+^ cells. Scale bar: 100 µm. (G) APP/PS1 Tg mice (n = 6 or 8/group) showed a marked increase in hippocampal Iba1^+^ABI3^+^ cells (left) and their ratio (right) versus C57BL/6 mice (*n* = 7/group). Data are presented as mean ± SEM. Statistical analyses were performed using an unpaired Student's *t*‐test (E, G) and one‐way analysis of variance (ANOVA) (C).

To elucidate the expression of ABI3 in the brains of APP/PS1 transgenic (Tg) mice, we performed single‐cell sorting followed by 10×Genomics transcriptome sequencing (single‐cell RNA sequencing, scRNA‐seq) on brain tissue from 8‐month‐old male APP/PS1 Tg mice. Our scRNA‐seq data confirmed that *Abi3* was expressed predominantly in microglia (Figure 1A, Figure S1A,B). This microglial enrichment was further validated by immunofluorescence staining, which showed strong ABI3 expression in Iba1^+^ microglia with minimal signal in neurons or astrocytes (Figure 1B,C). Consistent with previous scRNA‐seq studies, ABI3 is expressed predominantly in microglia and highly abundant in both human AD brains and APP/PS1 mice.^39^, ^40^ These cross‐species findings provided the basis for further characterization of ABI3 expression in APP/PS1 mice.

Spatial transcriptome profiling further revealed the expression of *Abi3* in the hippocampus and temporal cortex of APP/PS1 Tg mice (Figure 1D). RT‐qPCR analysis demonstrated significantly elevated *Abi3* mRNA levels in the cerebral cortex and hippocampus of APP/PS1 Tg mice compared with wild‑type (WT) littermates (Figure 1E). Immunofluorescence also confirmed that ABI3 was expressed in the hippocampal and cortical regions in WT littermates (Figure S1C).

Previous studies have reported increased ABI3 protein expression in the post‑mortem brain tissues from AD patients^18^, ^41^ To characterize the expression properties of ABI3 in the AD model, we compared 8‐month‐old WT and APP/PS1 Tg mice. Immunofluorescence staining showed significantly higher ABI3 expression in the microglia of APP/PS1 mice relative to WT controls (Figure 1F), as indicated by an increased number of Iba1^+^ABI3^+^ microglia and a higher proportion of Iba1^+^ABI3^+^/Iba1^+^ cells, consistent with earlier findings (Figure 1G).^17^, ^18^ Similarly, microglial immunostaining in 5×FAD mice also showed robust upregulation of ABI3 relative to WT mice (Figure S1D), confirming the upregulation of ABI3 in microglia across two AD mouse models.

Collectively, these results demonstrate that ABI3 is preferentially expressed in microglia, expressed in hippocampal and cortical regions, and significantly upregulated in multiple AD mouse models, suggesting a potential role for ABI3 in AD pathogenesis.

### 3.2 Microglia‐specific *ABI3* knockdown exacerbates amyloid pathology and impairs microglial migration and phagocytosis

Given that ABI3 is highly expressed in microglia and upregulated in AD models, we next examined its role in microglial function and amyloid pathology. We generated hippocampus‐targeted microglial ABI3‐knockdown (KD) models by delivering EGFP‐*Abi3*‐RNAi or EGFP‐control AAVs into 6‐month‐old APP/PS1 Tg and C57BL/6J WT control mice (Figure S2A). Quantification of GFP^+^/Iba1^+^ cells revealed comparable transduction between control and KD groups (Figure S2B). Immunofluorescence showed selective ABI3 reduction in microglia, but not in neurons or astrocytes, with regional and single‐cell confirmation (Figure S2C–E). Hippocampal mRNA and protein levels were also reduced (Figure S3A,B), collectively demonstrating efficient microglia‐specific *ABI3* KD. At 12 months of age, ABI3 depletion markedly aggravated amyloid pathology in APP/PS1 mice. Confocal imaging revealed increased Aβ plaque number, larger mean plaque diameter, and greater plaque area in AD+ABI3 KD mice compared to AD+Ctrl mice (Figure S3C,D). Microglia in AD+ABI3 KD mice exhibited reduced clustering around plaques, as indicated by diminished colocalization with Aβ deposits (Figure S3E,F). Morphometric analyses demonstrated profound defects in plaque‐associated microglia following ABI3 knockdown. Compared with controls, AD+ABI3 KD microglia displayed decreased cell diameter, shorter total branch and mean branch length, reduced maximal branch extension, and lower euclidean distance. The number of microglial branches and branching points was also significantly reduced in AD+ABI3 KD mice. (Figure S3E,G). Moreover, we evaluated metabolic parameters in our model. Body weight and hippocampal insulin receptor/CD36 levels were unchanged, indicating that microglial ABI3 knockdown does not replicate germline knockout metabolic phenotypes (Figure S4). Together, these findings demonstrated that microglial‐specific ABI3 depletion exacerbates amyloid plaque burden and disrupts microglial recruitment and phagocytic capacity, highlighting a critical role for ABI3 in maintaining microglial response to Aβ pathology.

### 3.3 ABI3 overexpression mitigates amyloid pathology in 5×FAD mice, and this effect is abolished by the *S209F* mutation

**FIGURE 2:**
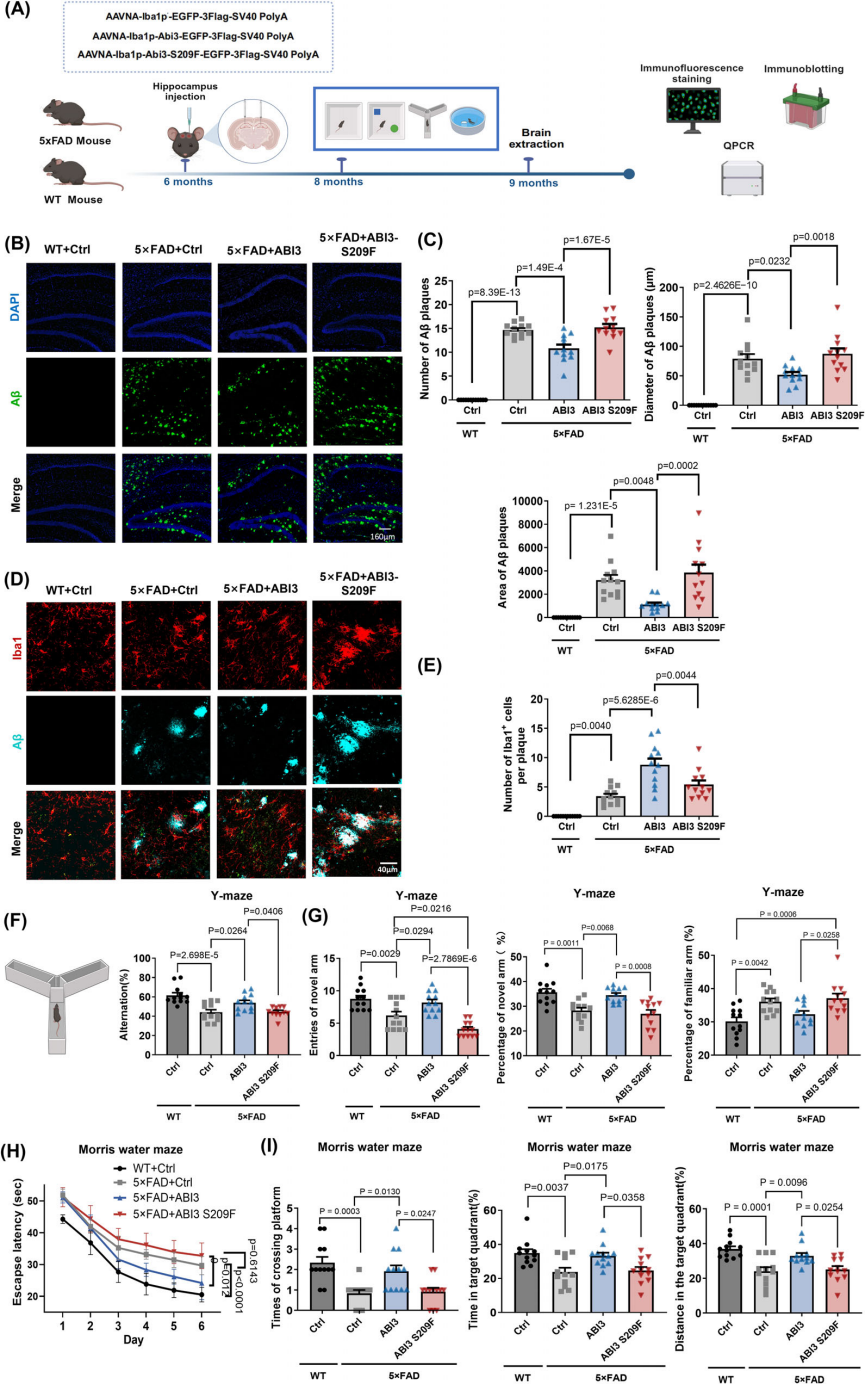
Microglia‐specific ABI3 overexpression mitigates amyloid pathology and improves spatial learning and memory, whereas the *S209F* variant fails to rescue disease progression in 5×FAD mice. (A) Timeline of the experimental procedures for C57BL/6 and 5×FAD mice. (B) Representative images of Aβ plaque deposition in the hippocampus of WT+Ctrl, 5×FAD+Ctrl, 5×FAD+ABI3, and 5×FAD+ *ABI3 S209F* mice. Scale bar: 160 µm. (C) Quantification of Aβ plaque number, Aβ plaque diameter, and Aβ plaque area (*n* = 12/group). (D) Morphology of microglia surrounding Aβ plaques in the hippocampus of all groups. Scale bar: 40 µm. (E) Quantification of microglia number surrounding Aβ plaques in the hippocampus of all groups (*n* = 12/group). (F) Spontaneous alternation rate measured in Y‐maze assay (*n* = 12/group). (G) Number and percentage of entries into the novel and familiar arm assessed in Y‐maze assay (*n* = 12/group). (H) Quantification of escape latency on various days in the Morris water maze assay (*n* = 12/group). (I) Number of platform crossings (left), percentage of time spent in the target quadrant (middle), and percentage of distance traveled in the target quadrant (right) in Morris water maze assay (*n* = 12/group). Data are presented as mean ± SEM. Statistical analyses were performed using one‐way ANOVA (C, E, F, G, I) and two‐way ANOVA (H).

Because microglial ABI3 depletion worsens amyloid pathology in AD models, we next asked whether ABI3 overexpression exerts protective effects and examined whether these effects are inhibited by the S209F mutation. To address this, we stereotaxically injected microglial‐specific EGFP‐ABI3, EGFP‐*ABI3 S209F*, or EGFP‐control AAVs into the hippocampus region of 6‐month‐old 5×FAD and WT mice (Figure 2A). Efficient overexpression was confirmed at 8 months by RT‐qPCR and western blot, and immunofluorescence validated hippocampal microglial‐specific targeting of ABI3 and *ABI3 S209F* with Iba1 staining (Figure S5A–C). Confocal imaging of Aβ‐stained hippocampal sections revealed that ABI3 overexpression reduced Aβ burden, with significantly fewer plaque numbers, shorter mean diameter, and smaller plaque area compared with 5×FAD controls. In contrast, expression of *ABI3 S209F* failed to attenuate Aβ plaque deposition in 5×FAD mice (Figure 2B,C). Notably, quantification of Aβ‐associated microglia clustering demonstrated enhanced microglial recruitment of Iba1^+^ cells to plaques in 5×FAD+ABI3 mice, whereas *ABI3 S209F* overexpression impaired this microglial migration (Figure 2D,E). Altogether, these findings revealed that microglial ABI3 overexpression mitigates amyloid pathology by promoting microglial recruitment to plaques under AD pathology, whereas the AD‐associated *ABI3 S209F* variant abolished these protective effects, failing to alleviate plaque burden or restore microglial responses.

### 3.4 ABI3 overexpression improves spatial learning and memory in 5×FAD mice, and this effect is abolished by the *S209F* mutation

We next assessed whether hippocampal overexpression of ABI3 or *ABI3 S209F* influences cognitive function in 5×FAD mice. In the open field test, no group differences were observed in locomotor activity or anxiety‐related parameters, indicating preserved basal motor function (Figure S6A–D). In the Y‐maze, 5×FAD+Ctrl mice exhibited reduced spontaneous alternation compared with WT+Ctrl, consistent with impaired working memory. ABI3 overexpression partially rescued this defect, whereas *ABI3 S209F* further decreased alternation rate (Figure 2F). In the novel arm exploration test, 5×FAD+Ctrl mice showed fewer novel arm entries and lower exploration percentage relative to WT+Ctrl. ABI3 overexpression enhanced novel arm exploration and reduced familiar arm preference, whereas *ABI3 S209F* produced the opposite effect (Figure 2G). In novel object recognition, no significant difference was observed in the percentage of recognition index (Figure S6E). In the Morris water maze, swimming speed was comparable across all groups (Figure S6F). During training, 5×FAD+ABI3 mice displayed faster acquisition, with shorter escape latencies and path lengths (Figure 2H, Figure S6G). In probe trials, these mice showed increased target quadrant occupancy and distance traveled within the target zone compared with 5×FAD+Ctrl. By contrast, 5×FAD+ *ABI3 S209F* mice exhibited impaired recall, with fewer platform crossings, reduced target quadrant dwell time, and shorter travel distance. Notably, direct comparison with 5×FAD+ABI3 confirmed that the *S209F* variant significantly diminished target quadrant time and travel distance (Figure 2I). Together, these results indicated that microglial ABI3 overexpression ameliorates spatial learning and memory defects in 5×FAD mice, whereas the *S209F* variant fails to improve cognitive dysfunction. These findings indicate that the *S209F* mutation abolishes the protective, microglia‐dependent cognitive benefits of ABI3 in AD.

### 3.5 ABI3 overexpression enhances microglial migration and phagocytosis, which are impaired by the *S209F* mutation

**FIGURE 3:**
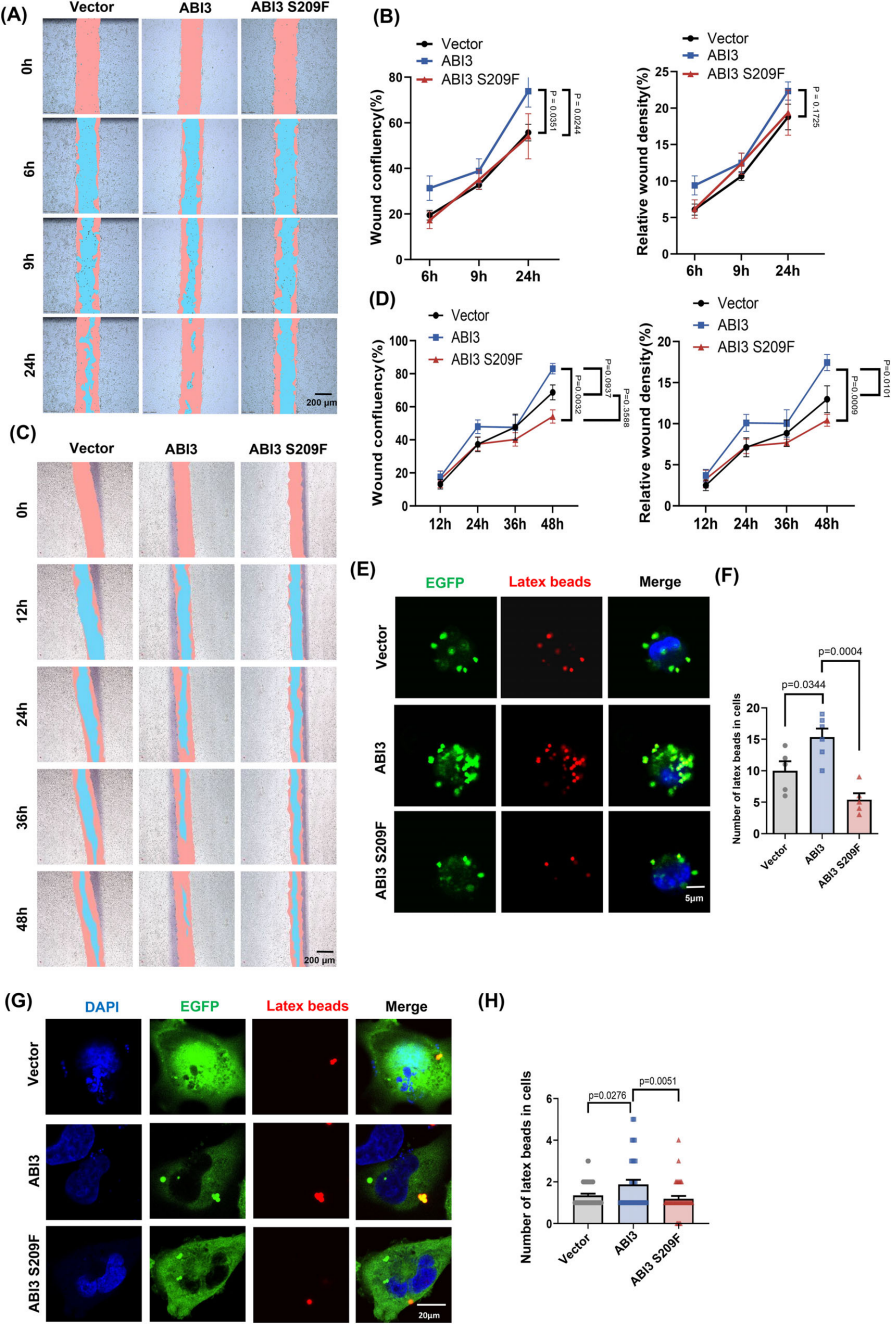
ABI3 enhances cell migration and phagocytosis, while *ABI3 S209F* inhibits these processes. (A) Representative images of scratch areas in HMC3 cells expressing ABI3 or *ABI3 S209F* at 6, 9, and 24 h. Scale bar: 200 µm. (B) Quantification of wound confluency and relative wound density in scratch areas from scratch assays in HMC3 cells. (C) Representative images of scratch areas in primary microglia expressing ABI3 or *ABI3 S209F* at 12, 24, 36, and 48 h. Scale bar: 200 µm. (D) Quantification of wound confluency and relative wound density in scratch areas from scratch assays in primary microglia. (E) ABI3‐overexpressing primary microglia displayed greater uptake of red fluorescent latex beads compared to *ABI3 S209F*. Scale bar: 5 µm. (F) Statistical graph showing the number of red fluorescent latex beads phagocytosed by primary microglia in each group. (G) ABI3‐overexpressing HMC3 cells displayed greater uptake of red fluorescent latex beads compared to controls. *ABI3 S209F* exhibited reduced uptake of red latex beads relative to ABI3. Scale bar: 20 µm. (H) Statistical graph showing the number of red fluorescent latex beads phagocytosed by HMC3 cells in each group. Data are presented as mean ± SEM. Statistical analyses were performed using one‐way ANOVA (F, H) and two‐way ANOVA (B, D).

To investigate how ABI3 and the *S209F* mutation influence AD pathogenesis through microglial function, we first examined the impact of the *ABI3 S209F* mutation on microglial migration, building upon previous reports that ABI3 regulates this process.^17^ Wound healing assays were conducted using HMC3 cells stably transduced with lentivirus expressing ABI3 or *ABI3 S209F*. Migratory capacities were assessed at 6, 9, and 24 h post‐scratch injury (Figure 3A), with wound healing rates and relative wound densities quantified (Figure 3B). The ABI3 overexpression group showed a time‐dependent enhancement of wound closure compared to vector controls, whereas the *ABI3 S209F* overexpression group exhibited significantly reduced wound healing rates versus ABI3 overexpression (Figure 3B). No significant differences in relative wound density were observed across groups over time. Following 100 nM Aβ stimulation, the ABI3 group displayed a marked increase in wound healing rate and relative wound density over time compared to vector controls, whereas the *ABI3 S209F* group showed a significant time‐dependent decrease in wound closure versus ABI3 (Figure S7A,B). To validate these findings, we performed migration assays in primary mouse microglia. Compared with the control group, primary microglia overexpressing WT ABI3 exhibited the strongest migration capacity, whereas those expressing *ABI3 S209F* mutant showed the weakest migration ability (Figure 3C,D). We next assessed phagocytic capacity in primary microglia that were transiently transfected with WT ABI3 or the *S209F* mutant. Twenty‐four hours post‐transfection, cells overexpressing WT ABI3 exhibited significantly higher phagocytic capacity compared with those expressing the *S209F* variant (Figures 3E,F). Consistent with these results, similar phagocytosis assays in HMC3 cells yielded comparable findings. HMC3 cells were transfected with ABI3, *ABI3 S209F*, or control plasmids, followed by incubation with 1.0 µm red fluorescent latex beads 24 h post‐transfection. Confocal microscopy revealed that ABI3‐overexpressing cells engulfed significantly more beads than controls, whereas *ABI3 S209F*‐overexpressing cells showed reduced bead uptake versus ABI3 overexpression in HMC3 cells (Figure 3G,H). Knockdown of *ABI3* via shRNA in HMC3 cells inhibited migration and phagocytosis, as confirmed by wound healing and bead uptake assays (Figure S8), consistent with previous studies.^17^ These results confirm that ABI3 promotes both phagocytosis and migration in HMC3 cells, whereas the *ABI3 S209F* mutation impairs these functions, highlighting its role in dysregulated microglial responses in AD.

### 3.6 *ABI3 S209F* mutation impairs its LLPS capacity in live cells

**FIGURE 4:**
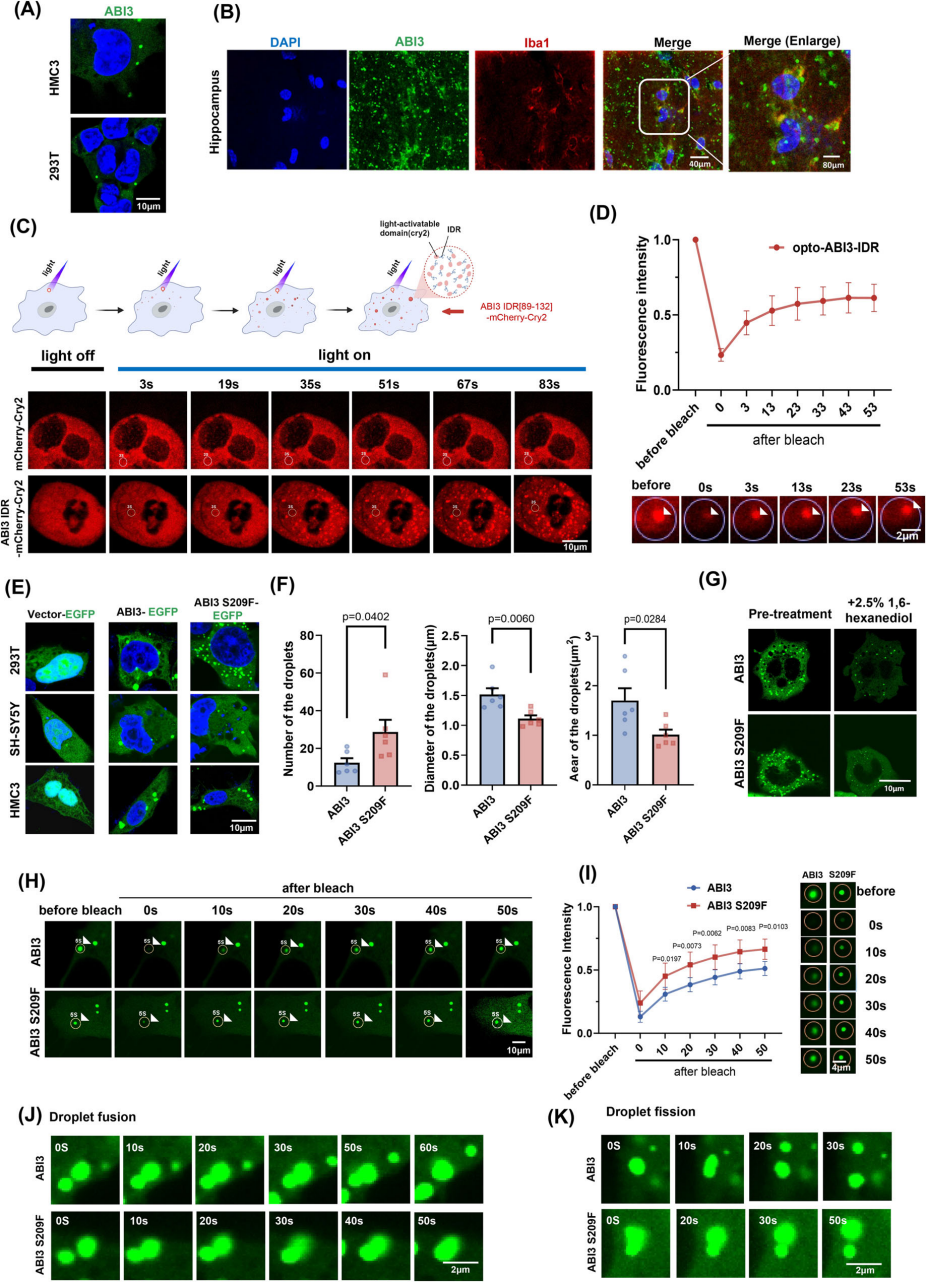
ABI3 and its mutant *ABI3 S209F* form cytoplasmic droplet‐like condensates via LLPS. (A) Endogenous ABI3 forms droplet‐like condensates in the cytoplasm of HEK 293T and HMC3 cells. Scale bar: 10 µm. (B) Endogenous ABI3 forms droplet‐like condensates inside microglia in the hippocampus of 5×FAD mice. Scale bar: 40 µm, 80 µm (enlarge). (C) Blue light irradiation activates optoABI3 IDR[89‐132]‐mCherry‐Cry2, inducing rapid aggregation and biomolecular condensate formation in living cells. Schematic diagram (top) and representative images (bottom). Scale bar: 10 µm. (D) Statistical graph of normalized fluorescence intensities from optoDroplet assays. Scale bar: 2 µm. (E) Droplet‐like condensates in cells. Overexpression of ABI3 and *ABI3 S209F* induces intracellular droplet‐like condensates in HEK 293T cells (top), HMC3 cells (middle), and SH‐SY5Y cells (bottom). Scale bar: 10 µm. (F) Statistical analysis showing fewer numbers (left panel), larger diameters (middle panel), and larger areas (right panel) in ABI3 droplet‐like condensates compared to *ABI3 S209F* in HEK 293T cells. (G) 1,6‐Hexanediol (1,6‐HD) disrupts the formation of ABI3 and *ABI3* *S209F* droplet‐like condensates. Scale bar: 10 µm. (H) In Fluorescence recovery after photobleaching (FRAP) assays, ABI3‐EGFP and *ABI3 S209F*‐EGFP droplets show fluorescence recovery. Scale bar: 10 µm. (I) Statistical graph of normalized fluorescence intensities from FRAP assays. *ABI3 S209F*‐EGFP droplets recover faster in HEK 293T cells. Scale bar: 4 µm. (J–K) Both ABI3‐EGFP and *ABI3 S209F*‐EGFP droplets undergo fusion (J) and fission (K) in HEK 293T cells. Scale bar: 2 µm. Data are presented as mean ± SEM. Statistical analyses were performed using unpaired Student's *t*‐test (F) and two‐way ANOVA (I).

Given that the *S209F* mutation impairs ABI3‐mediated microglial migration and phagocytosis, we next asked whether this mutation directly alters the biophysical properties of the ABI3 protein. Because phase separation has been reported to regulate the cytoskeleton,^26^ which is closely linked to cell migration, we first examined whether ABI3 exhibits phase separation properties. We observed that endogenous ABI3 formed distinct cytoplasmic droplet‐like condensates in both HEK293T and HMC3 cells (Figure 4A). Furthermore, similar droplet‐like condensates of ABI3 were also detected in the hippocampal region of mouse brain tissue (Figure 4B). These findings suggest that ABI3 is likely capable of undergoing phase separation. Using the optoDroplet system, we fused the ABI3 intrinsically disordered region (IDR, 89‐132 aa) to mCherry‐Cry2 (ABI3 IDR[89–132]‐mCherry‐Cry2). Blue light irradiation induced time‐dependent droplet formation in optoABI3 IDR‐expressing cells; control mCherry‐Cry2 cells showed no droplet aggregation, validating IDR‐mediated LLPS of ABI3 (Figure 4C,D). Next, we asked whether the S209F mutation affects the phase separation properties of ABI3. We overexpressed ABI3‐EGFP and *ABI3 S209F*‐EGFP in HEK293T, HMC3, and SH‐SY5Y cells, respectively, to examine the differences in condensate formation between the two proteins (Figure 4E). Quantitative analysis revealed significant differences in condensate properties: ABI3 yielded fewer condensates, but with larger diameter and area compared to *ABI3* *S209F* (Figure 4F). To rule out membrane‐bound organelle contributions, co‐localization studies with organelle markers confirmed that ABI3‐EGFP and *ABI3 S209F*‐EGFP condensates were not associated with membrane‐bound organelles (Figure S9A–F). Treatment with 1,6‐hexanediol (1,6‐HD), a disruptor of hydrophobic interactions, induced gradual dissolution of both ABI3 and *ABI3 S209F* condensates (Figure 4G, Figure S10B), confirming their LLPS dependence. The CCK‐8 assays revealed that cell viability decreased gradually with prolonged treatment time of 1,6‐HD. However, the concentration and time points used in our study exerted negligible effects on cell viability (Figure S10A). FRAP experiments showed dynamic molecular exchange properties of condensates (Figure 4H), with *ABI3 S209F* condensates exhibiting faster recovery kinetics relative to ABI3, indicative of altered molecular exchange rates (Figure 4H,I). Live‐cell imaging revealed rapid movement, fusion (Figure 4J), and fission (Figure 4K) of both ABI3 and *ABI3 S209F* condensates, further supporting their liquid‐like behavior. Molecular dynamics modeling revealed that the *S209F* mutation disrupts hydrogen bonding and key hydrophobic interactions, while inducing new π–π and cation–π interactions that remodel the LLPS‑prone interaction network, thereby weakening condensate stability (Figure S11). Altogether, these findings establish that ABI3 undergoes phase separation and demonstrate that the *S209F* mutation perturbs condensate formation and dynamics, providing a mechanistic link to its role in AD pathogenesis.

### 3.7 *ABI3 S209F* mutation impairs its LLPS capacity in vitro

**FIGURE 5:**
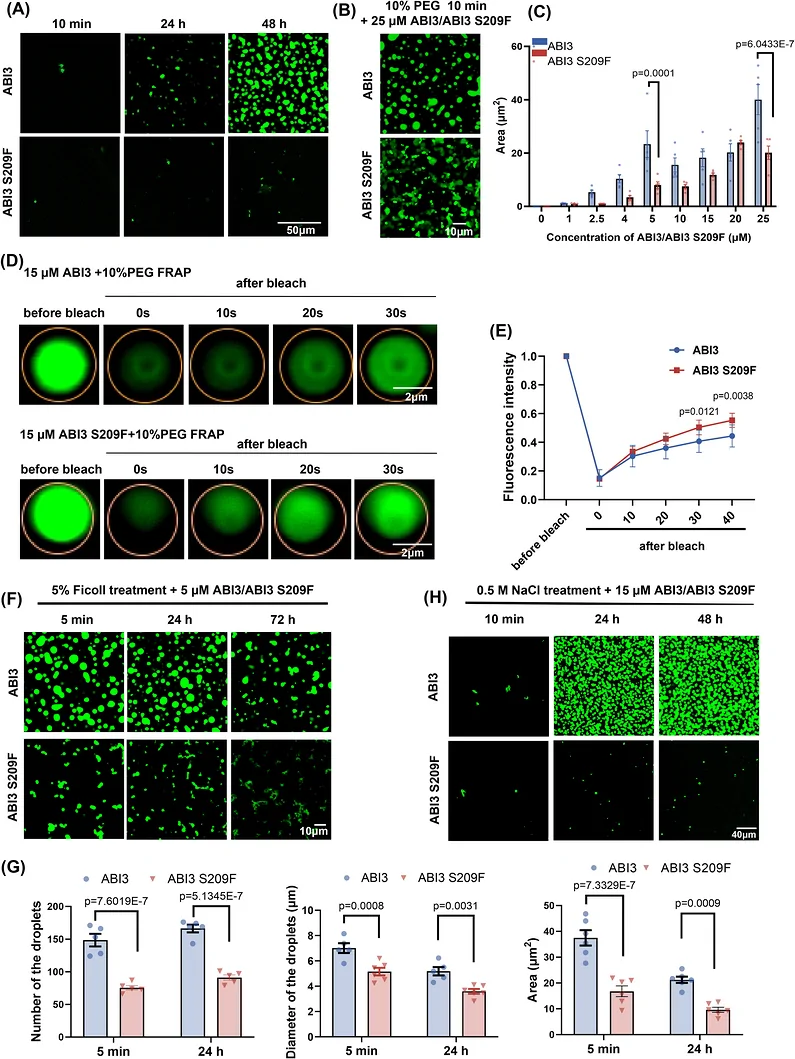
*ABI3 S209F* recombinant protein exhibits weaker LLPS capacity than ABI3 in vitro. (A) ABI3 (10 µM) and *ABI3 S209F* (10 µM) formed droplet‐like aggregates following incubation at room temperature for varying durations; *ABI3 S209F* formed fewer aggregates than ABI3 under identical conditions. Scale bar: 50 µm. (B–C) Condensates were formed by ABI3 or *ABI3 S209F* (high concentration 25 µM) mixed with 10% PEG 8000 for 10 min in vitro. *ABI3 S209F* formed smaller areas and irregular phase‐separated droplets than ABI3. Representative images with 25 µM proteins (B), scale bar: 10 µm; Quantification of condensate areas with various protein concentrations (C). (D–E) Fluorescence of droplet‐like aggregates recovered rapidly in FRAP assays. Representative images (D), Scale bar: 2 µm. Quantification of fluorescence intensity (E). (F–G), Condensates were formed by ABI3 or *ABI3 S209F* (5 µM) mixed with 5% Ficoll 400 for different times in vitro. Representative images (F), scale bar: 10 µm. Statistical analysis of cytoplasmic droplet aggregate counts (left panel), diameters (middle panel), and areas (right panel) (G). (H) Representative fluorescence images of LLPS in vitro after mixing 15 µM ABI3 or *ABI3 S209F* with 0.5 M NaCl for different times. Scale bar: 40 µm. Data are presented as mean ± SEM. Statistical analyses were performed using two‐way ANOVA (C, E, G).

To directly assess the intrinsic phase separation capacity of ABI3 and exclude intracellular confounding factors, we performed in vitro LLPS assays using purified GFP‐tagged recombinant ABI3 and *ABI3 S209F* proteins (Figure S12A,B). At 10 µM protein incubation, both proteins formed droplet‐like condensates in a time‐dependent manner, with ABI3 yielding larger and more abundant condensates than *ABI3* *S209F* (Figure 5A). In addition to the macromolecular crowding agent polyethylene glycol (PEG 8000, 10%), we observed that 25 µM ABI3 or *ABI3* *S209F* induced turbidification, a hallmark of LLPS (Figure S12C). Turbidity assays at *λ* = 600 nm confirmed that these changes occurred in both time‐ and concentration‐dependent manners (Figure S12D). In addition, treatment with 5% 1,6‐HD dissolved pre‐formed condensates, confirming their dependency on LLPS (Figure S12E). Moreover, at 15 µM protein incubation with PEG 8000, both ABI3 and *ABI3* *S209F* condensates showed rapid movement, fusion (Figure S12F), and fission (Figure S12G), further supporting their liquid‐like behavior in vitro. With PEG 8000, higher protein concentration increased the number and size of ABI3 condensates, whereas the *ABI3* *S209F* variant formed hydrogel‐like structures at high concentrations. ABI3 condensates were more abundant, larger, and more regular in shape compared with those formed by *ABI3* *S209F* (Figure 5B,C, Figure S13). Consistent with cellular observations, FRAP analysis demonstrated that both proteins maintain liquid‐like properties, but *ABI3* *S209F* condensates recovered fluorescence more rapidly than ABI3, suggesting altered molecular dynamics in vitro (Figure 5D,E).

Using Ficoll 400 as an alternative crowding agent, ABI3 maintained droplet structures with increasing concentration, whereas *ABI3 S209F* transitioned to rosary‐like assemblies (Figure S14). Under 5% Ficoll 400 treatment, ABI3 formed more numerous and larger condensates than *ABI3 S209F* (Figure 5F,G). Notably, *ABI3 S209F* developed irregular hydrogel networks at high concentrations and underwent transitions from droplets to fibrous structures over time (Figure 5F, Figure S14–S15). FRAP also revealed faster recovery kinetics in *ABI3 S209F* condensates, consistent with its altered dynamics (Figure S15F). Phase separation properties were also modulated by ionic strength. Across both low (50 mM) and high (0.5 M) NaCl conditions, ABI3 consistently formed more abundant and larger condensates than *ABI3 S209F*, suggesting the higher stability of ABI3‐forming condensates under high‐salt environments (Figure 5H, Figure S16–S17). Collectively, these in vitro results demonstrated that ABI3 possesses stronger intrinsic LLPS capacity, whereas the *S209F* mutation disrupts condensate formation and phase separation dynamics, promoting aberrant phase transitions under diverse crowding and ionic environments.

### 3.8 Ser209 phosphorylation and IDR regulate ABI3 phase separation

**FIGURE 6:**
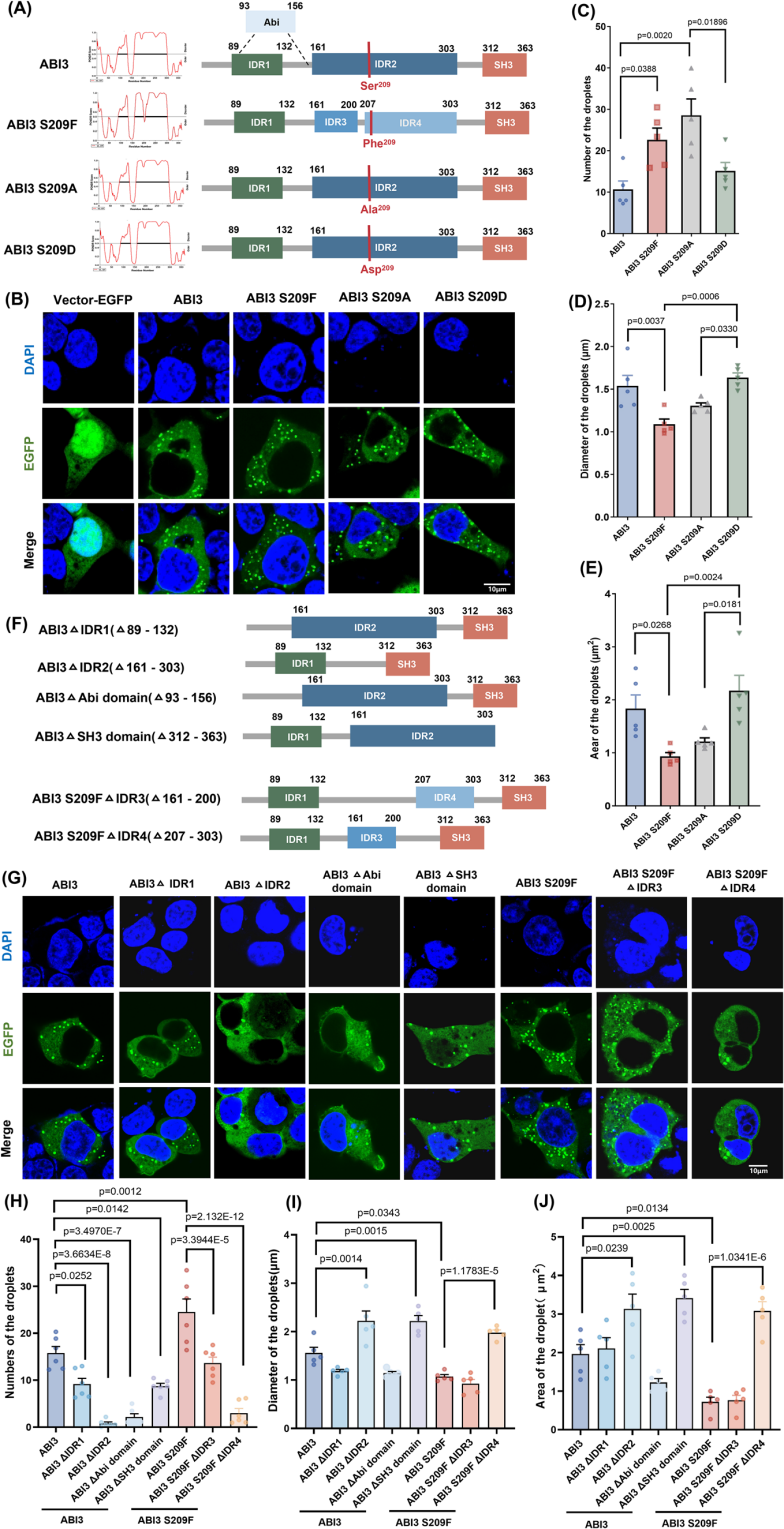
*ABI3 S209D* mutation promotes ABI3 phase separation, with intrinsically disordered region 2 (IDR2) (161–303) and SH3 domains (312–363) as key functional regions. (A) Schematic of ABI3 and S209 mutants. Diagram of ABI3 and its S209 mutant variants. (B) Laser confocal imaging of cytoplasmic droplets in HEK 293T cells 24 h after transfection with ABI3‐EGFP, *ABI3 S209F*‐EGFP, *ABI3 S209A*‐EGFP, or *ABI3 S209D*‐EGFP overexpression plasmids. Scale bar: 10 µm. (C–E), Statistical analysis of droplet condensate counts (C), diameters (D), and areas (E) in ABI3‐EGFP, *ABI3 S209F*‐EGFP, *ABI3 S209A*‐EGFP, and *ABI3 S209D*‐EGFP groups. (F) Diagram of ABI3 structural domains and IDR deletion variants of ABI3 and *ABI3 S209F*. (G) Laser confocal imaging of droplets in HEK 293T cells 24 h after transfection with ABI3‐EGFP, *ABI3 S209F*‐EGFP, or their domain deletion variants (ΔIDR1, ΔIDR2, ΔIDR3, ΔIDR4, ΔAbi, ΔSH3). Scale bar: 10 µm. (H–J), Statistical analysis of cytoplasmic droplet aggregate counts (H), diameters (I), and areas (J) in HEK 293T cells overexpressing EGFP fused with ABI3 or its domain deletion variants. Data are presented as mean ± SEM. Statistical analyses were performed using one‐way ANOVA (C, D, E, H, I, J).

Given the distinct LLPS properties of ABI3 and *ABI3 S209F*, we next interrogated structural determinants of this behavior. LLPS kinetics are often regulated by PTMs of intrinsically disordered proteins (IDPs), which modulate electrostatic and hydrophobic interactions. Ser209 of ABI3 is a known phosphorylation site, and the *S209F* mutation impairs its phosphorylation. To probe its functional relevance, we generated phosphomimetic (*S209D*) and non‐phosphorylatable (*S209A*) mutants to assess PTM effects on LLPS. HEK 293T cells were transfected with ABI3‐EGFP, *ABI3 S209F*‐EGFP, *ABI3 S209A*‐EGFP, or *ABI3 S209D*‐EGFP plasmids (Figure 6A,B). Confocal imaging revealed that the *S209A* mutation increased droplet number but reduced condensate diameter and area, whereas the *S209D* mutation decreased droplet number while promoting larger condensates (Figure 6C–E). Molecular dynamics modeling showed that the *S209A* variant exhibits RMSD fluctuations and hydrophobic interactions comparable to those of *S209F*. In contrast, the *S209D* mutant increases side‐chain polarity and interacts with the nearby positively charged Arg201, forming a stable electrostatic structure (Figure S11). These trends parallel the differences observed between WT ABI3 and *ABI3 S209F*, suggesting that phosphorylation at Ser209—blocked by the *S209F* mutation—may enhance the LLPS capacity of ABI3.

To further dissect structural contributions, we examined IDRs that represent a key determinant of LLPS. PONDR analysis revealed that WT ABI3 contains two IDRs: IDR1 (89‐132) and IDR2 (161‐303), and the *S209F* mutation splits IDR2 (161‐303) into two discrete regions: IDR3 (161‐200) and IDR4 (207‐303) (Figure 6A). We generated a series of truncated mutants of both WT ABI3 and the *ABI3 S209F* variant, each harboring deletions of individual IDRs or the SH3 domain (Figure 6F). For WT ABI3, deletion of IDR2 or the SH3 domain significantly altered condensate properties, producing fewer but larger droplets, with IDR2 loss exerting the most pronounced effect. In the *S209F* background deletion of IDR4 substantially disrupted droplet formation, affecting number, diameter, and area (Figure 6G–J). Together, these findings identify Ser209 phosphorylation and the IDR2 (161‐303) and IDR4 (207‐303) as critical regulators of ABI3 phase separation, when the SH3 domain (312‐363) functions as a key structural regulator of this process. These results provide mechanistic insight into how the *S209F* mutation perturbs ABI3 condensate dynamics through combined effects on phosphorylation and IDR architecture.

### 3.9 FUS IDR rescues LLPS defect and restores migration and phagocytosis in HMC3 cells expressing the *ABI3 S209F* mutant

**FIGURE 7:**
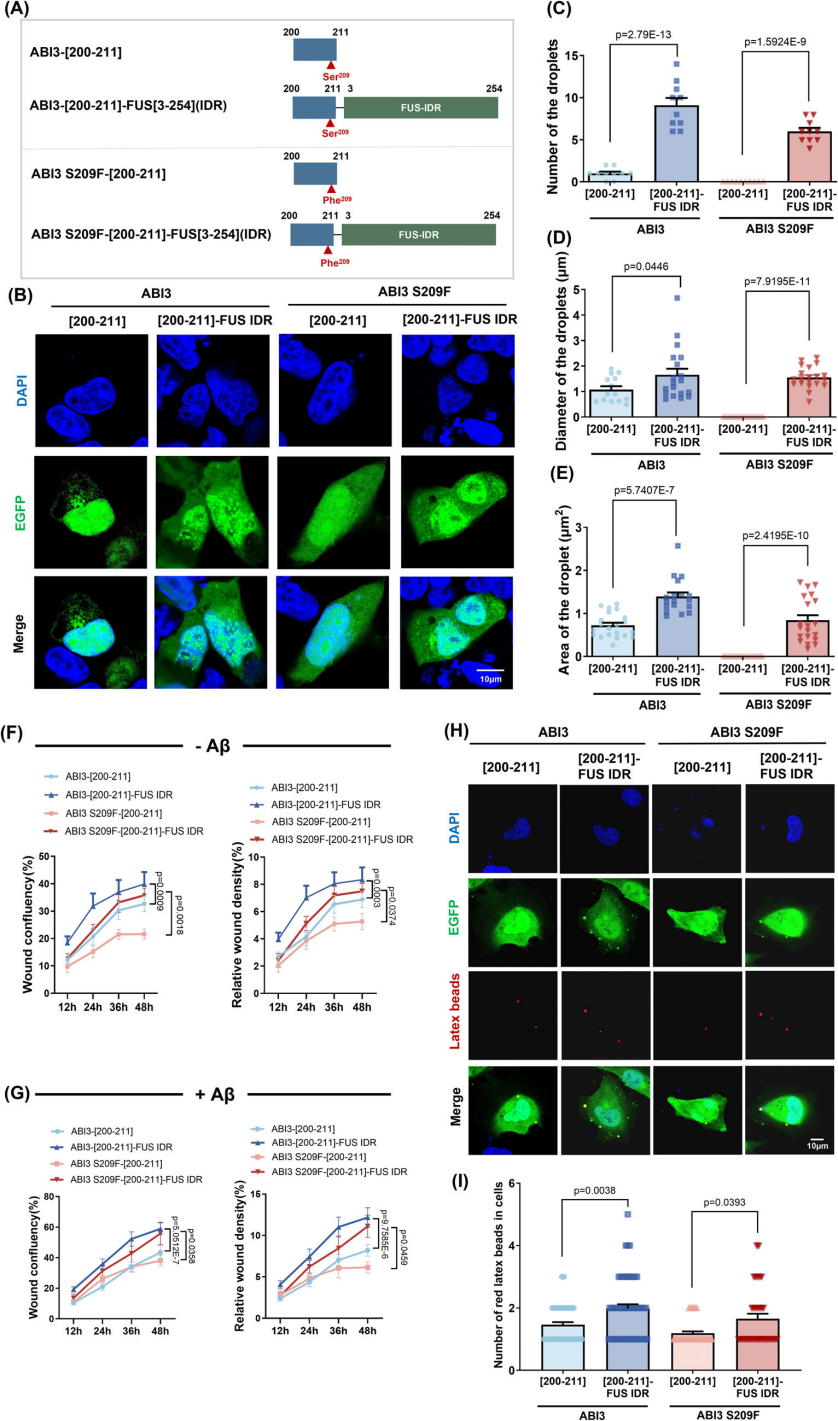
Impairment of phase separation and cell migration/phagocytosis in cells expressing ABI3‐F209‐[200–211], and reversal of these defects by FUS IDR fusion. (A) Schematic of ABI3 variants and FUS IDR fusions. (B) Confocal imaging of cytoplasmic droplet aggregates in HEK 293T cells 24 h after transfection with ABI3 variants and FUS IDR fusion plasmids. ABI3‐F209‐[200–211] lacks droplet‐forming capacity. Scale bar: 10 µm. Analysis of droplet aggregate counts (C), diameters (D), and areas (E) in HEK 293T cells 24 h after transfection with the indicated plasmids. (F–G), Statistical analysis of wound healing rates and relative wound density in HMC3 cells expressing the four ABI3 variants at 12, 24, 36, and 48 h without (F) or with (G) 100 nM Aβ stimulation, via scratch assay. (H–I), Fusion with FUS IDR improves HMC3 cell uptake of red fluorescent latex beads. Representative images (H), scale bar: 10 µm. Quantification of the number of red fluorescent latex inside cells (I). Data are presented as mean ± SEM. Statistical analyses were performed using two‐way ANOVA (F–G) and one‐way ANOVA (C, D, E, I).

To further interrogate the role of IDR in ABI3 phase separation, we examined whether FUS IDR can compensate for ABI3 LLPS defects. We generated fusion constructs in which FUS IDR[3–254] and ABI3 mutants, including *ABI3 S209F*‐FUS IDR, ABI3‐ΔSH3 domain‐FUS IDR, ABI3‐ΔIDR2‐FUS IDR, and *ABI3 S209F* ΔIDR4‐FUS IDR (Figure S18A). Transfection with these plasmids in HEK293T cells, FUS IDR significantly enhanced condensate formation in *ABI3 S209F*‐FUS IDR compared with *S209F* alone, increasing droplet number, diameter, and area (Figure S18B–E). Similar rescue effects were observed in ABI3 ΔSH3‐FUS IDR and *ABI3 S209F* ΔIDR4‐FUS IDR relative to their parental mutants. By contrast, ABI3 ΔIDR2‐FUS IDR and *ABI3 S209F* ΔIDR4‐FUS IDR showed increased droplet number but with reduced diameter and area compared with ABI3 ΔIDR2 alone, indicating context‐dependent effects of FUS IDR on ABI3 LLPS properties (Figure S18B–E). To focus on the region surrounding residue 209, we constructed ABI3‐S209‐[200–211] and ABI3‐F209‐[200–211] fragments fused to EGFP (Figure 7A). Confocal imaging revealed that ABI3‐F209‐[200–211] failed to produce detectable condensates, whereas ABI3‐S209‐[200–211] formed small condensates (Figure 7B). Strikingly, fusion with FUS IDR restored condensate formation in both fragments, leading to robust droplet formation with increased number, diameter, and area (Figure 7C–E). Together, these results demonstrate that the FUS IDR can partially rescue LLPS deficits caused by the *ABI3 S209F* mutation or key domain deletions, with the residue 209‐containing peptide fragment [200–211] playing a critical role in mediating phase separation defects. The ability of FUS IDR to restore condensate formation in the critical 200–211 region highlights the critical role of multivalent interactions in regulating ABI3 LLPS dynamics.

To validate the importance of ABI3's LLPS capacity for microglial function, we next examined whether FUS IDR could also rescue the migration and phagocytosis defects in HMC3 cells expressing the *S209F* mutant. The wound healing assays were conducted in HMC3 cells expressing ABI3‐S209‐[200–211], ABI3‐F209‐[200–211], or their FUS IDR fusion constructs (Figure S19). Migratory capacity was assessed at 12, 24, 36, and 48 h post‐scratch injury, with wound healing rates and relative wound densities quantified. Fusion of FUS IDR significantly enhanced the wound healing rate over time and relative wound density (Figure 7F). Upon Aβ stimulation, fusion with FUS IDR further enhanced wound healing rate and relative wound density in both ABI3‐F209‐[200–211] and ABI3‐S209‐[200–211] (Figure 7G). For phagocytosis assays, HMC3 cells were transfected with ABI3‐S209‐[200–211], ABI3‐F209‐[200–211], or their FUS IDR fusions, followed by incubation with 1.0 µm red fluorescent latex beads (Figure 7H). Confocal imaging showed that fusion with FUS IDR significantly increased bead uptake (Figure 7I). These results confirmed that the IDR integrating strategy can significantly improve migration and phagocytosis in HMC3 cells, thereby indicating that the enhancement of ABI3 phase separation can mitigate AD pathogenesis via improved microglial response.

### 3.10 Phase separation by ABI3 and *ABI3 S209F* differentially regulates microglial functions

To directly test whether LLPS is required for ABI3‐mediated microglial function, we treated ABI3‐ and *ABI3 S209F*‐overexpressing HMC3 or BV2 cells with 1,6‐HD, a compound disrupting LLPS via weak hydrophobic interactions (Figure S20–S23). Treatment with 1,6‐HD phenocopied the *S209F* mutation, significantly impairing both migration (HMC3: Figure S20A–S21B; BV2: Figure S22A–S23B) and phagocytosis of red fluorescent latex beads (HMC3: Figure S20C–21D; BV2: Figure S22C–23D) to levels comparable to those observed in S209F‐expressing cells.

To further evaluate the role of IDR in ABI3 LLPS, we generated an *ABI3 S209F*‐ABI3 IDR2 fusion construct. In wound‐healing assays, fusion with IDR2 significantly enhanced migration rate and relative wound density in both HMC3 (Figure S24,B) and BV2 cells (Figure S25A,B). Phagocytosis assays similarly showed that IDR2 fusion markedly increased latex bead uptake in HMC3 (Figure S24C,D) and BV2 cells (Figure S25C,D), demonstrating that the ABI3 IDR2 improves migration and phagocytosis.

Finally, optogenetic experiments using optoABI3 IDR[89–132]‐mCherry‐Cry2 confirmed the direct role of ABI3 LLPS in microglial activity. Blue light stimulation induced robust increases in both phagocytosis (HMC3: Figures S26A,B; BV2: Figures S27A,B) and migration (HMC3: Figures S26C–E; BV2: Figures S27C–E) selectively in cells expressing the optoABI3 IDR construct, whereas control cells showed no response.

Together, these complementary loss‐of‐function (1,6‐HD) and gain‐of‐function (IDR fusion and optogenetic activation) experiments establish a causal link between impaired LLPS and the microglial dysfunction induced by the *ABI3 S209F* mutation.

## 4 DISCUSSION

**FIGURE 8:**
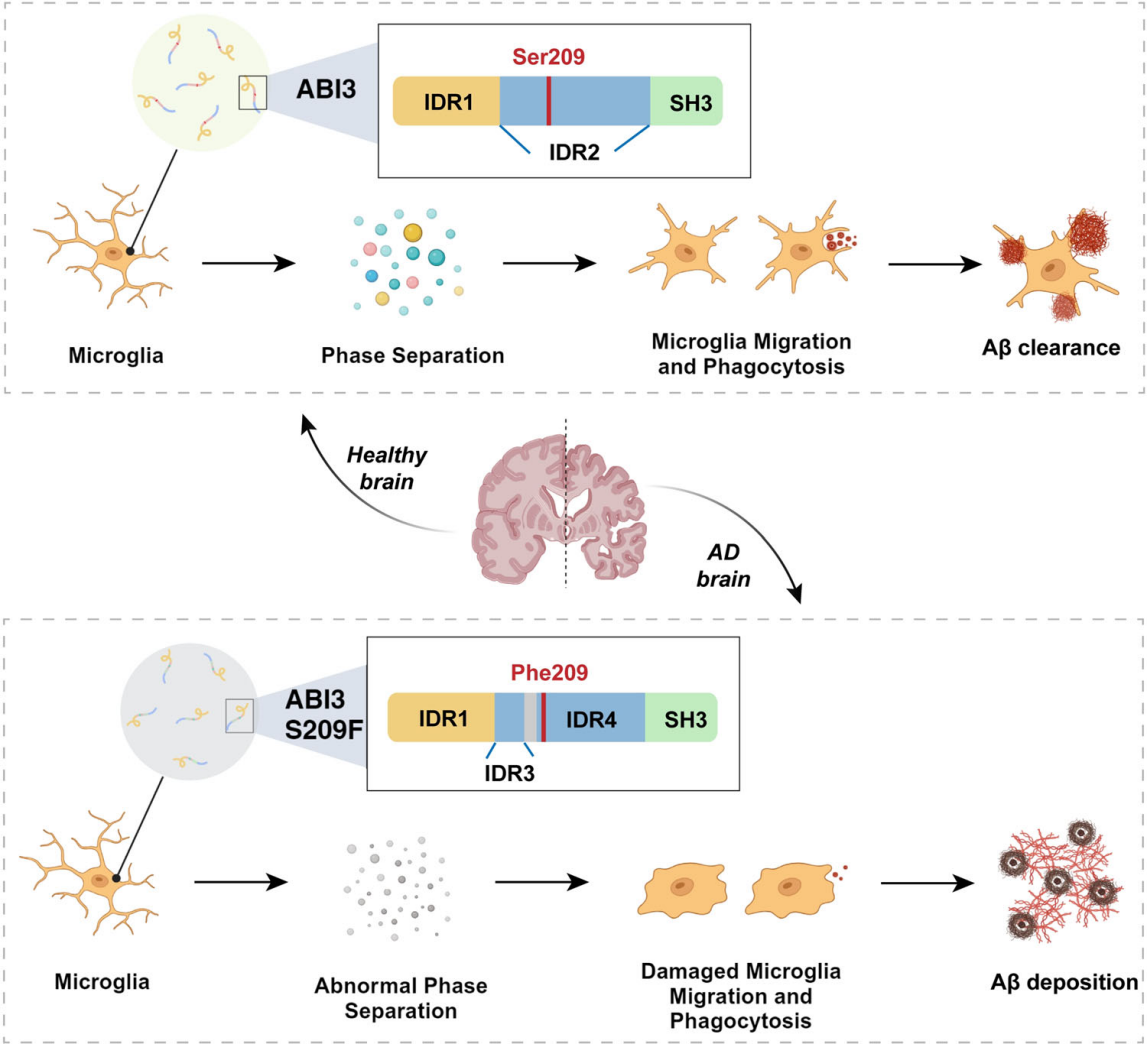
Mechanistic model: *ABI3 S209F* increases AD risk by disrupting phase separation and subsequently inhibiting microglial migration, phagocytosis, and clearance of Aβ plaques.

Late‐onset Alzheimer's disease (or LOAD) accounts for the majority of AD cases, making it critical to elucidate the functional mechanisms of LOAD susceptibility genes. Although many LOAD risk genes are preferentially expressed in microglia and have emerged as central players in AD pathogenesis, their precise functions remain largely unclear. Notably, the rare *ABI3 S209F* variant has recently been genetically associated with increased AD risk, with heritability comparable to that of well‐known LOAD susceptibility genes such as *TREM2*.^15^ Elevated ABI3 expression has been reported in the brains of patients with AD and in amyloid/tau mouse models,^18^, ^41^ yet the functional significance of ABI3 and the mechanism by which *ABI3 S209F* elevates AD risk remain unclear. To address these gaps, this study reveals for the first time a novel mechanism by which ABI3 regulates microglial function through liquid–liquid phase separation (or LLPS)—a mechanism not previously implicated in any microglial AD risk gene. We demonstrate that WT ABI3 undergoes LLPS, facilitating microglial clustering around amyloid plaques, enhancing phagocytosis, and promoting Aβ clearance; in contrast, the *S209F* mutation disrupts this phase separation capability, leading to impaired microglial migration and compromised neuroprotective functions (Figure 8). Using bidirectional functional manipulations (including acute LLPS disruption with 1,6‐HD and rescue of LLPS by fusing with the FUS‐IDR),^30^, ^42^ we establish that LLPS disruption is the causal mechanism underlying the *S209F* phenotype, not merely an epiphenomenon. These findings identify ABI3 as a previously unrecognized phase‐separating neuroprotective protein and reveal a non‐aggregative LLPS mechanism linking genetic variation to microglial dysfunction and AD pathogenesis.

Consistent with recent genetic analyses showing that most LOAD risk genes are microglia‐enriched (e.g., *APOE*, *TREM2*, and *PICALM*),^9^, ^10^, ^11^, ^12^ our single‐cell and spatial transcriptomics data confirm that *Abi3* is highly and specifically expressed in microglia surrounding hippocampal plaques (Figure 1A–D), consistent with previous reports of *Abi3* upregulation in AD mouse models.^17^, ^18^ At the functional level, prior studies showed that ABI3 deficiency exacerbates AD pathology.^1^ ^7^ Here, we extend these findings by demonstrating that the *S209F* mutation similarly impairs microglial migration and phagocytosis. Gain‐ and loss‐of‐function experiments further reveal that WT ABI3 enhances microglial Aβ clearance, whereas the *S209F* variant lacks this protective effect, resulting in increased amyloid accumulation.

To dissect the specific impact of the *S209F* mutation on disease progression, we performed microglia‐specific knockdown and overexpression experiments in two AD mouse models (APP/PS1 and 5×FAD). Knockdown of endogenous Abi3 in APP/PS1 mice exacerbated amyloid plaque pathology; overexpression of WT ABI3 in 5×FAD mice significantly rescued cognitive deficits, reduced Aβ burden, and enhanced microglial aggregation around plaques. In contrast, overexpression of the *S209F* mutant completely abolished these neuroprotective effects. Other studies using germline Abi3 knockout mice have yielded partially conflicting results: one reported increased Aβ aggregation and impaired phagocytosis,^17^ whereas another observed reduced Aβ deposition but exacerbated tau pathology,^18^ and neither assessed cognitive deficits. These discrepancies may arise from differences in models, sex, or pathological background. The advantage of our microglia‐specific manipulation is that it directly dissects cell‐autonomous functions. We acknowledge that the exclusive use of male mice constitutes a limitation of this study, limiting the generalizability of our conclusions to female animals and human populations. That said, Karahan et al. reported that *Abi3* deletion similarly exacerbates Aβ pathology in female mice,^17^ suggesting that the core mechanism may not be fundamentally sex dependent. Nevertheless, direct experimental validation in both sexes is still required to confirm whether the microglial phenotypes identified here are fully conserved across sexes. We noted distinct metabolic outcomes between our model and germline *Abi3* knockout mice.^19^ Our AAV‐mediated, hippocampus‐specific microglial ABI3 knockdown avoids global gene deletion and hypothalamic impairment. No altered body weight or metabolic gene expression was detected, confirming that our localized manipulation does not induce systemic metabolic dysfunction. We acknowledge that flow cytometry would provide an ideal complementary approach for quantifying cell type–specific knockdown at the population level. However, despite extensive testing of multiple commercially available anti‐ABI3 antibodies, we were unable to identify one that produces specific and reliable signal for flow cytometric detection of endogenous mouse *Abi3*. This technical limitation prevents us from providing flow cytometry data at this time. Moreover, although the APP/PS1 and 5×FAD models are valuable for studying amyloid‐driven mechanisms,^43^, ^44^, ^45^ they have limitations in recapitulating the slow, age‐dependent progression of human AD. Given the extremely low allele frequency of *ABI3 S209F* (minor allele frequency [MAF] ≈0.008),^15^, ^20^ obtaining endogenous human samples is challenging. Therefore, we plan to generate *S209F* knock‐in mice to assess long‐term functional consequences under the endogenous promoter and better recapitulate physiological conditions. Meanwhile, future studies using human induced pluripotent stem cell (iPSC)–derived microglia in chimeric models will further validate the generalizability of our results to low‐pathology, sporadic AD‐like conditions in humans.

Phase separation (liquid‑liquid phase‑separation, LLPS) plays an important role in the pathological mechanisms of neurodegenerative diseases. Classical phase‐separating proteins such as FUS,^46^ TDP‐43,^47^ tau,^25^, ^29^ and α‐synuclein^48^ exhibit abnormal accumulation and aggregation that are closely linked to neuronal dysfunction. However, most current LLPS studies have focused on these aggregation‐prone proteins, which tend to form excessive condensates under pathological conditions and subsequently undergo a liquid‐to‐solid transition, thereby exacerbating disease progression.^24^ In addition, LLPS plays key roles in fundamental biological processes such as cytoskeletal regulation,^26^ synaptic signaling,^49^ and stress granule formation.^50^, ^51^ For example, cytoskeletal proteins such as neural Wiskott‑Aldrich syndrome protein (N‐WASP) undergo phase separation to regulate actin dynamics. Although ABI3 is a component of the N‐WASP complex,^14^ its LLPS properties have never been reported. This study demonstrates for the first time that ABI3 forms cytoplasmic condensates when overexpressed in multiple cell types. Using FRAP, droplet fusion, photoactivated optoDroplet experiments in live cells, and in vitro reconstitution, we confirm that ABI3 indeed possesses LLPS capability. Of note, WT ABI3 and the *S209F* mutant exhibit distinct LLPS properties: in live cells, WT ABI3 forms fewer but larger condensates with slower FRAP recovery; in vitro, WT ABI3 forms condensates more robustly than the *S209F* mutant under identical conditions. Therefore, the *S209F* mutation impairs microglial migration and neuroprotective function by disrupting ABI3 LLPS. This finding introduces the LLPS mechanism into the functional regulation of a microglial AD risk gene and reveals a paradigm distinct from classical aggregative proteins, namely that loss of LLPS capacity, rather than excessive aggregation, increases disease risk. Consistent with the in vivo findings of Butler et al., who reported that *Abi3 S212F* mutation impairs plaque compaction and disrupts microglial homeostasis, our data further suggest that the *S209F* variant exerts its pathogenic effects through altered phosphorylation and disrupted LLPS, providing a mechanistic basis for the observed microglial dysfunction.^52^

Post‐translational modifications (PTMs) are key regulators of LLPS, and phosphorylation, in particular, has been widely reported to affect the phase separation of various neurodegenerative disease‐related proteins.^29^, ^53^ Serine 209 (Ser 209) of ABI3 is a phosphorylatable residue; mutation to phenylalanine (Phe) abolishes this phosphorylation capacity.^18^ Previous studies hypothesized that *S209F* might affect the overall phosphorylation status of ABI3, thereby influencing its function.^18^ To exclude the possibility that the *S209F* mutation simply reduces ABI3 protein stability, we compared the expression levels of WT and *ABI3* *S209F* in HEK293T cells. As shown in Figure S28, the mutant protein was expressed at levels comparable to that of WT, ruling out a simple loss‐of‐expression mechanism. Using AlphaFold modeling and molecular dynamics simulations, we found that the *S209F* mutation reduces the overall structural stability of ABI3, increases flexibility, enhances hydrophobic and π–π interactions, and decreases the hydrogen bond network—all changes that can affect LLPS.^54^ We further generated a phosphomimetic mutation (*S209D*) and a control mutation (*S209A*) and found that *S209D* promotes phase separation, whereas *S209A* inhibits it, confirming the critical regulatory role of phosphorylation status on LLPS. At the domain level, ABI3 contains two intrinsic disordered regions (IDR1[89‐132] and IDR2[161‐303]), whereas the *S209F* mutation causes IDR2 to break into two shorter fragments (IDR3[161‐200], and IDR4[207‐303]). Domain deletion experiments demonstrate that IDR2 and IDR4 are essential for ABI3 phase separation, suggesting that the LLPS defect of *S209F* originates from the shortening of its IDRs. Notably, fusing the FUS‐IDR to *ABI3* *S209F* effectively rescues condensate formation as well as microglial migration and phagocytosis, providing proof‐of‐concept for future gene therapy strategies that enhance ABI3 LLPS capacity by integrating an IDR.

In summary, this study elucidates from a structural biology perspective the mechanism by which *ABI3 S209F* causes LLPS dysfunction and reveals the unique role of ABI3 as a non‐aggregative phase‐separating protein in AD neuroprotection. Although the current work demonstrates the potential of ABI3 LLPS in suppressing AD progression, the underlying fine molecular regulatory networks (e.g., interacting proteins, upstream signals) remain to be explored. In the future, we will identify ABI3‐interacting proteins, investigate how they cooperatively regulate microglial migration, and develop small‐molecule strategies targeting these interactors to enhance ABI3 LLPS capacity, ultimately providing a new avenue for AD therapy.

## CONFLICT OF INTEREST STATEMENT

All authors declare no competing interests. Author disclosures are available in the Supporting Information.

## CONSENT STATEMENT

Consent was not necessary as human subjects were not used in this study.

## Supporting information



Supporting Information

Supporting Information

Supporting Information

Supporting Information

Supporting Information

Supporting Information

Supporting Information

Supporting Information

Supporting Information

Supporting Information

Supporting Information

Supporting Information

Supporting Information

Supporting Information

Supporting Information

Supporting Information

Supporting Information

Supporting Information

Supporting Information

Supporting Information

Supporting Information

Supporting Information

Supporting Information

Supporting Information

Supporting Information

Supporting Information

Supporting Information

Supporting Information

Supporting Information

Supporting Information

## References

[1] 2025 Alzheimer's disease facts and figures. Alzheimers Dement. 2025;21:1‐119. doi:10.1038/alz.70235

[2] Zhang J , Zhang Y , Wang J , Xia Y , Zhang J , Chen L . Recent advances in Alzheimer's disease: mechanisms, clinical trials and new drug development strategies. Signal Transduct Target Ther. 2024;9(1):211. doi:10.1038/s41392-024-01911-3 PMC11344989 39174535

[3] Long JM , Holtzman DM . Alzheimer disease: an update on pathobiology and treatment strategies. Cell. 2019;179(2):312‐339. doi:10.1016/j.cell.2019.09.001 PMC6778042 31564456

[4] Hunter S , Brayne C . Understanding the roles of mutations in the amyloid precursor protein in Alzheimer disease. Mol Psychiatry. 2018;23(1):81‐93. doi:10.1038/mp.2017.218 29112196

[5] Kelleher RJ 3rd, Shen J. Presenilin‐1 mutations and Alzheimer's disease. Proc Natl Acad Sci U S A. 2017;114(4):629‐631. doi:10.1073/pnas.1619574114 PMC5278466 28082723

[6] Bellenguez C , Küçükali F , Jansen IE , et al. New insights into the genetic etiology of Alzheimer's disease and related dementias. Nat Genet. 2022;54(4):412‐436. doi:10.1038/s41588-022-01024-z PMC9005347 35379992

[7] Wightman DP , Jansen IE , Savage JE , et al. A genome‐wide association study with 1,126,563 individuals identifies new risk loci for Alzheimer's disease. Nat Genet. 2021;53(9):1276‐1282. doi:10.1038/s41588-021-00921-z PMC10243600 34493870

[8] Leng F , Edison P . Neuroinflammation and microglial activation in Alzheimer disease: where do we go from here?. Nat Rev Neurol. 2021;17(3):157‐172. doi:10.1038/s41582-020-00435-y 33318676

[9] Victor MB , Leary N , Luna X , et al. Lipid accumulation induced by APOE4 impairs microglial surveillance of neuronal‐network activity. Cell Stem Cell. 2022;29(8):1197‐1212.e8. doi:10.1016/j.stem.2022.07.005 PMC9623845 35931030

[10] Haney MS , Pálovics R , Munson CN , et al. APOE4/4 is linked to damaging lipid droplets in Alzheimer's disease microglia. Nature. 2024;628(8006):154‐161. doi:10.1038/s41586-024-07185-7 PMC10990924 38480892

[11] Ulland TK , Song WM , Huang SC , et al. TREM2 maintains microglial metabolic fitness in Alzheimer's disease. Cell. 2017;170(4):649‐663.e13. doi:10.1016/j.cell.2017.07.023 PMC5573224 28802038

[12] Kozlova A , Zhang S , Sudwarts A , et al. PICALM Alzheimer's risk allele causes aberrant lipid droplets in microglia. Nature. 2025;646(8087):1178‐1186. doi:10.1038/s41586-025-09486-x PMC12571902 40903578

[13] Gomez AR , Byun HR , Wu S , et al. Boosting angiotensin‐converting enzyme (ACE) in microglia protects against Alzheimer's disease in 5×FAD mice. Nat Aging. 2025;5(7):1280‐1294. doi:10.1038/s43587-025-00879-1 40490625

[14] Kim HG , Berdasco C , Nairn AC , Kim Y . The WAVE complex in developmental and adulthood brain disorders. Exp Mol Med. 2025;57(1):13‐29. doi:10.1038/s12276-024-01386-w PMC11799376 39774290

[15] Sims R , van der Lee SJ , et al. Rare coding variants in PLCG2, ABI3, and TREM2 implicate microglial‐mediated innate immunity in Alzheimer's disease. Nat Genet. 2017;49(9):1373‐1384. doi:10.1038/ng.3916 PMC5669039 28714976

[16] Conway OJ , Carrasquillo MM , Wang X , et al. ABI3 and PLCG2 missense variants as risk factors for neurodegenerative diseases in Caucasians and African Americans. Mol Neurodegener. 2018;13(1):53. doi:10.1186/s13024-018-0289-x PMC6190665 30326945

[17] Karahan H , Smith DC , Kim B , et al. Deletion of Abi3 gene locus exacerbates neuropathological features of Alzheimer's disease in a mouse model of Aβ amyloidosis. Sci Adv. 2021;7(45):eabe3954. doi:10.1126/sciadv.abe3954 PMC8565913 34731000

[18] Ibanez KR , McFarland KN , Phillips J , et al. Deletion of Abi3/Gngt2 influences age‐progressive amyloid β and tau pathologies in distinctive ways. Alzheimers Res Ther. 2022;14(1):104. doi:10.1186/s13195-022-01044-1 PMC9327202 35897046

[19] Smith DC , Karahan H , Wijeratne HRS , et al. Deletion of the Alzheimer's disease risk gene Abi3 locus results in obesity and systemic metabolic disruption in mice. Front Aging Neurosci. 2022;14:1035572. doi:10.3389/fnagi.2022.1035572 PMC9813750 36620768

[20] Koivumäki M , Martiskainen H , Takalo M , et al. Genetic validation of ABI3 p.Ser209Phe variant and its effects on early brain pathology in asymptomatic elderly individuals. Alzheimers Res Ther. 2026;18(1):67. doi:10.1186/s13195-026-01984-y PMC13023201 41709289

[21] Ahmed R , Forman‐Kay JD . Aberrant phase separation: linking IDR mutations to disease. Cell Res. 2023;33(8):583‐584. doi:10.1038/s41422-023-00804-4 PMC10397348 37016021

[22] Shimobayashi SF , Ronceray P , Sanders DW , Haataja MP , Brangwynne CP . Nucleation landscape of biomolecular condensates. Nature. 2021;599(7885):503‐506. doi:10.1038/s41586-021-03905-5 34552246

[23] Xie F , Zhou X , Ran Y , et al. Targeting FOXM1 condensates reduces breast tumour growth and metastasis. Nature. 2025;638(8052):1112‐1121. doi:10.1038/s41586-024-08421-w 39814884

[24] Zbinden A , Pérez‐Berlanga M , De Rossi P , Polymenidou M . Phase separation and neurodegenerative diseases: a disturbance in the force. Dev Cell. 2020;55(1):45‐68. doi:10.1016/j.devcel.2020.09.014 33049211

[25] Boyko S , Surewicz WK . Tau liquid‐liquid phase separation in neurodegenerative diseases. Trends Cell Biol. 2022;32(7):611‐623. doi:10.1016/j.tcb.2022.01.011 PMC9189016 35181198

[26] Cheng X , Case LB . Condensates control the actin cytoskeleton. Dev Cell. 2025;60(11):1519‐1520. doi:10.1016/j.devcel.2025.04.015 40494278

[27] Han KA , Ko J . Orchestration of synaptic functions by WAVE regulatory complex‐mediated actin reorganization. Exp Mol Med. 2023;55(6):1065‐1075. doi:10.1038/s12276-023-01004-1 PMC10318009 37258575

[28] Li Y , Liu Y , Yu XY , et al. Membraneless organelles in health and disease: exploring the molecular basis, physiological roles and pathological implications. Signal Transduct Target Ther. 2024;9(1):305. doi:10.1038/s41392-024-02013-w PMC11570651 39551864

[29] Wegmann S , Eftekharzadeh B , Tepper K , et al. Tau protein liquid‐liquid phase separation can initiate tau aggregation. EMBO J. 2018;37(7):e98049. doi:10.15252/embj.201798049 PMC5881631 29472250

[30] Liu X , Jiang S , Ma L , et al. Time‐dependent effect of 1,6‐hexanediol on biomolecular condensates and 3D chromatin organization. Genome Biol. 2021;22(1):230. doi:10.1038/s13059-021-02455-3 PMC8369800 34404453

[31] Shin Y , Berry J , Pannucci N , et al. Spatiotemporal control of intracellular phase transitions using light‐activated optoDroplets. Cell. 2017;168(1‐2):159‐171. doi:10.1016/j.cell.2016.11.054 PMC5562165 28041848

[32] Liang C , Zou T , Zhang M , et al. MicroRNA‐146a switches microglial phenotypes to resist the pathological processes and cognitive degradation of Alzheimer's disease. Theranostics. 2021;11(9):4103‐4121. doi:10.7150/thno.53418 PMC7977456 33754051

[33] Serrano C , Cananzi S , Shen T , et al. Simple and highly specific targeting of resident microglia with adeno‐associated virus. iScience. 2024;27(9):110706. doi:10.1016/j.isci.2024.110706 PMC11407971 39297168

[34] Okada Y , Hosoi N , Matsuzaki Y , et al. Development of microglia‐targeting adeno‐associated viral vectors as tools to study microglial behavior in vivo. Commun Biol. 2022;5(1):1224. doi:10.1038/s42003-022-04200-3 PMC9652230 36369525

[35] Aoki R , Konno A , Hosoi N , et al. AAV vectors for specific and efficient gene expression in microglia. Cell Rep Methods. 2025;5(8):101116. doi:10.1016/j.crmeth.2025.101116 PMC12461629 40744011

[36] Saura J , Tusell JM , Serratosa J . High‐yield isolation of murine microglia by mild trypsinization. Glia. 2003;44(3):183‐189. doi:10.1002/glia.10274 14603460

[37] Frazel PW , Labib D , Fisher T , et al. Longitudinal scRNA‐seq analysis in mouse and human informs optimization of rapid mouse astrocyte differentiation protocols. Nat Neurosci. 2023;26(10):1726‐1738. doi:10.1038/s41593-023-01424-2 PMC10763608 37697111

[38] Zhang Z , Sun X , Liu Y , et al. Spatial transcriptome‐wide profiling of small cell lung cancer reveals intra‐tumoral molecular and subtype heterogeneity. Adv Sci (Weinh). 2024;11(31):e2402716. doi:10.1002/advs.202402716 PMC11336901 38896789

[39] Olah M , Menon V , Habib N , et al. Single cell RNA sequencing of human microglia uncovers a subset associated with Alzheimer's disease. Nat Commun. 2020;11(1):6129. doi:10.1038/s41467-020-19737-2 PMC7704703 33257666

[40] Turner AK , Shaw BC , Simpson JF , Estus S . Identification and quantitation of novel ABI3 isoforms relative to Alzheimer's disease genetics and neuropathology. Genes (Basel). 2022;13(9):1607. doi:10.3390/genes13091607 PMC9498898 36140776

[41] Satoh JI , Kino Y , Yanaizu M , et al. Microglia express ABI3 in the brains of Alzheimer's disease and Nasu‐Hakola disease. Intractable Rare Dis Res. 2017;6(4):262‐268. doi:10.5582/irdr.2017.01073 PMC5735279 29259854

[42] Xu M , Jiang SY , Tang S , et al. Nuclear SREBP2 condensates regulate the transcriptional activation of lipogenic genes and cholesterol homeostasis. Nat Metab. 2025;7(5):1034‐1051. doi:10.1038/s42255-025-01291-0 40394324

[43] Pilat DJ , Le H , Prokopenko D , et al. TREM2 R47H impairs microglial phase separation and amyloid plaque clearance in mice. Neuron. 2026;114(1):46‐66.e13. doi:10.1016/j.neuron.2025.09.032

[44] Takalo M , Jeskanen H , Rolova T , et al. The protective PLCγ2‐P522R variant mitigates Alzheimer's disease‐associated pathologies by enhancing beneficial microglial functions. J Neuroinflammation. 2025;22(1):64. doi:10.1186/s12974-025-03387-6 PMC11881468 40038760

[45] Butler CA , O'Gara K , Mendoza‐Arvilla A , et al. 5×FAD/Abi3S209F mice display distinct age‐dependent effects on amyloid beta pathology and microglia dynamics. Alzheimers Dement. 2025;20(Suppl 1):e089879. doi:10.1002/alz.089879

[46] Murthy AC , Dignon GL , Kan Y , et al. Molecular interactions underlying liquid‐liquid phase separation of the FUS low‐complexity domain. Nat Struct Mol Biol. 2019;26(7):637‐648. doi:10.1038/s41594-019-0250-x PMC6613800 31270472

[47] Gruijs da Silva LA, Simonetti F , Hutten S , et al. Disease‐linked TDP‐43 hyperphosphorylation suppresses TDP‐43 condensation and aggregation. EMBO J. 2022;41(8):e108443. doi:10.15252/embj.2021108443 PMC9016352 35112738

[48] Ray S , Singh N , Kumar R , et al. α‐Synuclein aggregation nucleates through liquid‐liquid phase separation. Nat Chem. 2020;12(8):705‐716. doi:10.1038/s41557-020-0465-9 32514159

[49] Chen X , Wu X , Wu H , Zhang M . Phase separation at the synapse. Nat Neurosci. 2020;23(3):301‐310. doi:10.1038/s41593-019-0579-9 32015539

[50] Meng F , Li H , Huang Y , et al. RIOK1 phase separation restricts PTEN translation via stress granules activating tumor growth in hepatocellular carcinoma. Nat Cancer. 2025;6(7):1223‐1241. doi:10.1038/s43018-025-00984-5 40467995

[51] Yang P , Mathieu C , Kolaitis RM , et al. G3BP1 is a tunable switch that triggers phase separation to assemble stress granules. Cell. 2020;181(2):325‐345.e28. doi:10.1016/j.cell.2020.03.046 PMC7448383 32302571

[52] Butler CA , Gee MS , O'Gara K , et al. Abi3S212F Alzheimer's disease variant alters plaque structure and disrupts microglia. Alzheimers Dement. 2026;22(5):e71452. doi:10.1002/alz.71452 PMC13149227 42092345

[53] Ren Q , Li L , Liu L , et al. The molecular mechanism of temperature‐dependent phase separation of heat shock factor 1. Nat Chem Biol. 2025;21(6):831‐842. doi:10.1038/s41589-024-01806-y 39794489

[54] Gabryelczyk B , Cai H , Shi X , et al. Hydrogen bond guidance and aromatic stacking drive liquid‐liquid phase separation of intrinsically disordered histidine‐rich peptides. Nat Commun. 2019;10(1):5465. doi:10.1038/s41469-019-13469-8 PMC6884462 31784535

