# Supplementary material for: Aberrant phase separation from a rare ABI3 mutation drives microglial dysfunction and Alzheimer's risk
Source: Alzheimers Dement. 2026 Sep 24;22(9):e71812. doi: 10.1002/alz.71812 (PMC13602175; doi:10.1002/alz.71812)
Supplement: Supplementary file 1 [file ALZ-22-e71812-s001.pdf]

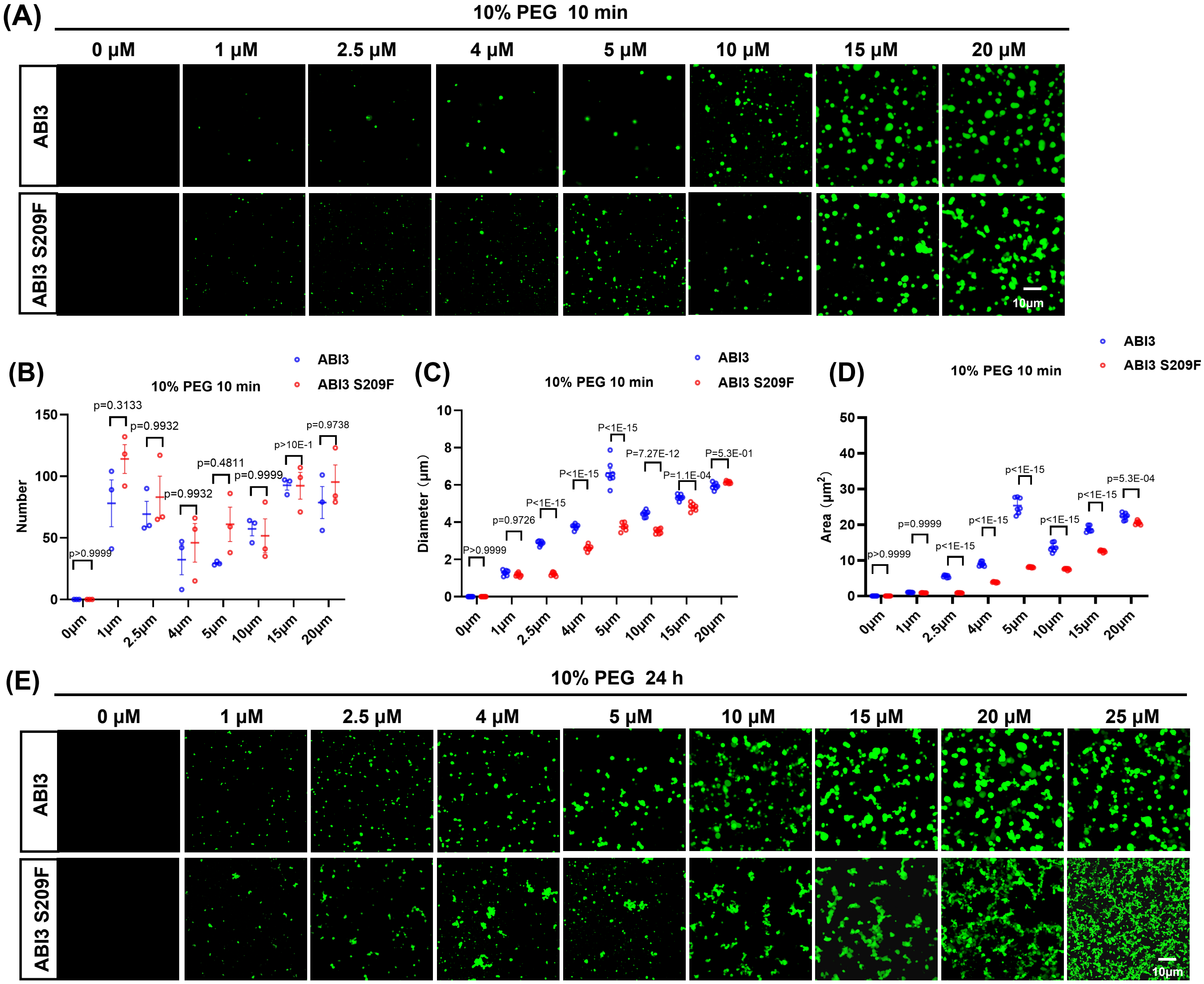


**SUPPLEMENTARY FIGURE 13 ABI3 and *ABI3 S209F* exhibit distinct phase separation properties in the presence of 10% PEG 8000.**

**(A)** Representative LLPS fluorescence images. Fluorescence microscopy of LLPS in vitro after 10-minute incubation of ABI3 or *ABI3 S209F* with 10% PEG 8000 at different protein concentrations. Scale bar: 10 μm. **(B)** Statistical analysis of droplet counts formed by ABI3 or *ABI3 S209F* mixed with 10% PEG 8000 for 10 minutes in vitro (varying protein concentrations). **(C)** Quantification of droplet diameters after mixing ABI3 or *ABI3 S209F* with 10% PEG 8000 for 10 minutes in vitro (varying concentrations). *ABI3 S209F* droplets were smaller than ABI3 droplets at matching concentrations. **(D)** Quantification of droplet areas after mixing ABI3 or *ABI3 S209F* with 10% PEG 8000 for 10 minutes in vitro (varying concentrations). **(E)** Representative fluorescence images of ABI3 and *ABI3 S209F* mixed with 10% PEG 8000 in vitro for 24 hours (varying concentrations). *ABI3 S209F* was more prone to forming fibrous structures at high protein concentrations (20 μM, 25 μM). Scale bar: 10 μm. Data presentation: Data are presented as mean ± SEM. Statistical analyses were performed using two-way ANOVA **(B-D)**.
