# Supplementary material for: Aberrant phase separation from a rare ABI3 mutation drives microglial dysfunction and Alzheimer's risk
Source: Alzheimers Dement. 2026 Sep 24;22(9):e71812. doi: 10.1002/alz.71812 (PMC13602175; doi:10.1002/alz.71812)
Supplement: Supplementary file 2 [file ALZ-22-e71812-s002.pdf]

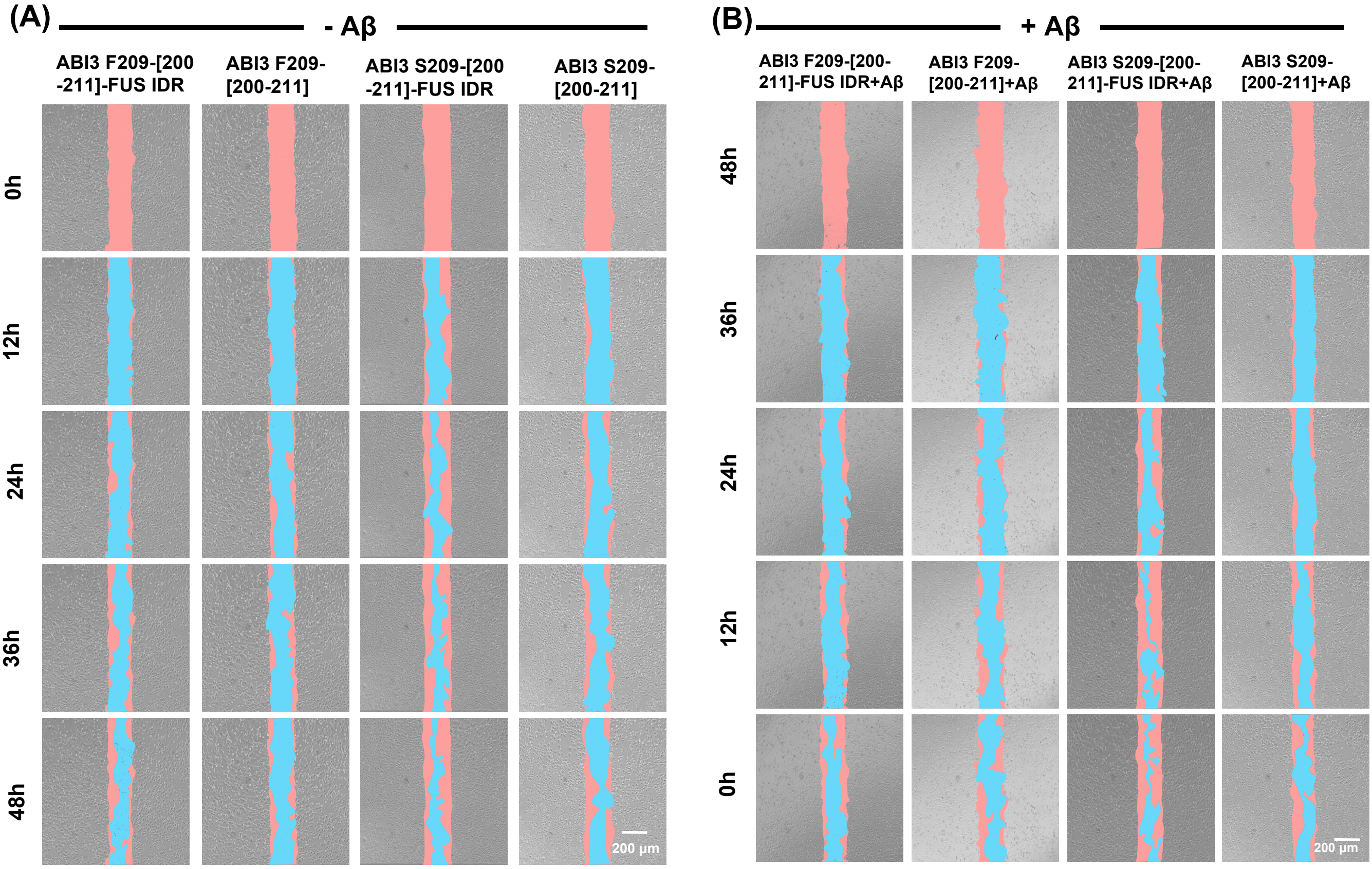

SUPPLEMENTARY FIGURE 19 ABI3-F209-[200-211] inhibits HMC3 cell migration, reversed by ABI3-F209-[200-211]-FUS IDR[3-254].

Representative scratch area images of HMC3 cells expressing ABI3-S209-[200-211], ABI3-F209-[200-211], ABI3-S209-[200-211]-FUS IDR[3-254], or ABI3-F209-[200-211]-FUS IDR[3-254] at 12 h, 24 h, 36 h, and 48 h with (**B**) or without (**A**) Aβ stimulation. Scale bar: 200 μm.
