# Supplementary material for: Aberrant phase separation from a rare ABI3 mutation drives microglial dysfunction and Alzheimer's risk
Source: Alzheimers Dement. 2026 Sep 24;22(9):e71812. doi: 10.1002/alz.71812 (PMC13602175; doi:10.1002/alz.71812)
Supplement: Supplementary file 4 [file ALZ-22-e71812-s004.pdf]

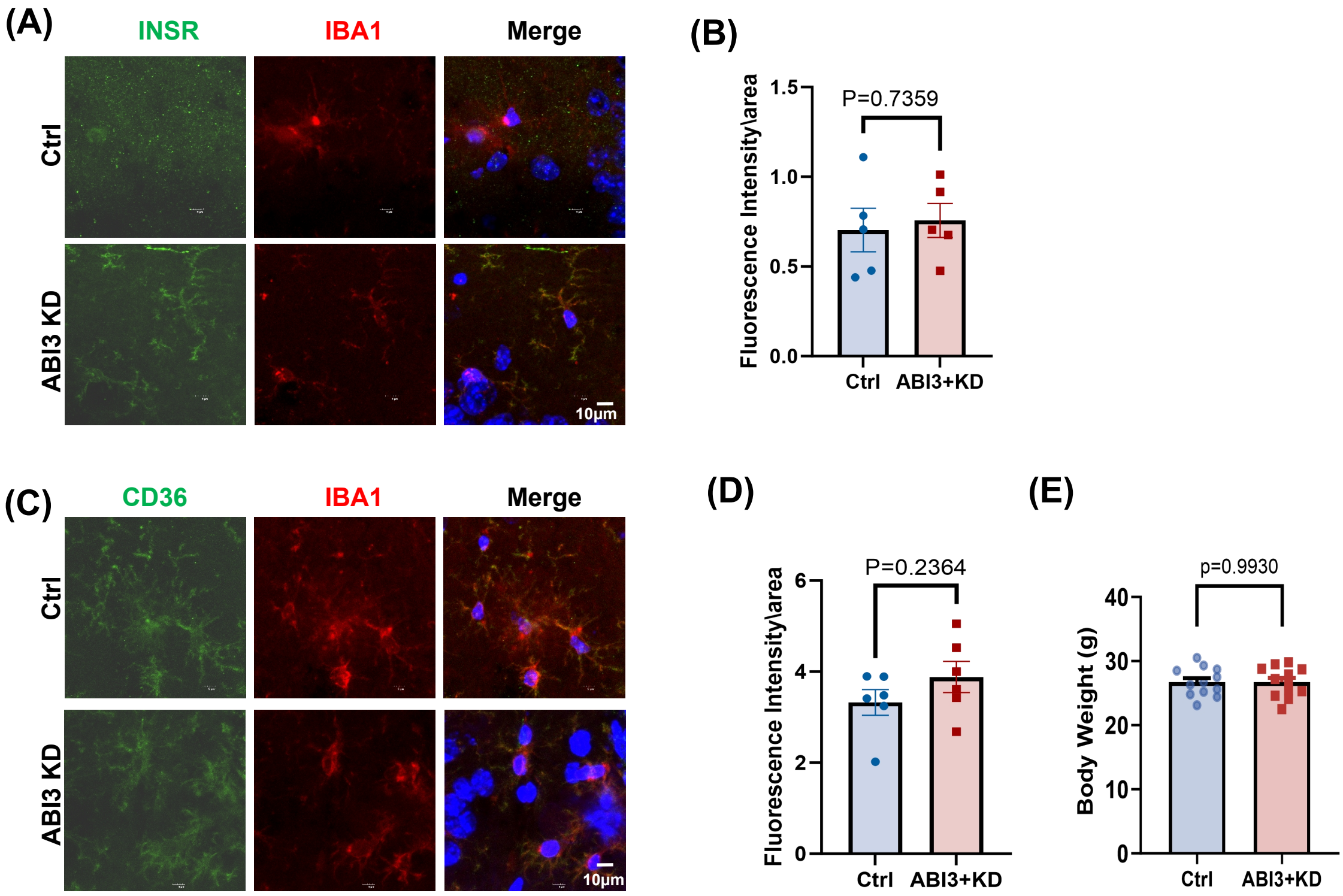

SUPPLEMENTARY FIGURE 4 ABI3 knockdown does not affect metabolic function in APP/PS1 mice.

Representative confocal images **(A)** and quantification of insulin receptors (InsR) **(B)**. Scale bar: 10 μm. (n = 5). Representative confocal images **(C)** and quantification of CD36 **(D)** Scale bar: 10 μm. (n = 5). **(E)** Body weight was measured in 12-month-old male AD+Ctrl (n = 10), and AD+ABI3 KD mice (n = 10). Data are presented as mean ± SEM. Statistical analyses were performed using unpaired Student's t-test **(B, D, E).**
