# Supplementary material for: Aberrant phase separation from a rare ABI3 mutation drives microglial dysfunction and Alzheimer's risk
Source: Alzheimers Dement. 2026 Sep 24;22(9):e71812. doi: 10.1002/alz.71812 (PMC13602175; doi:10.1002/alz.71812)
Supplement: Supplementary file 5 [file ALZ-22-e71812-s005.pdf]

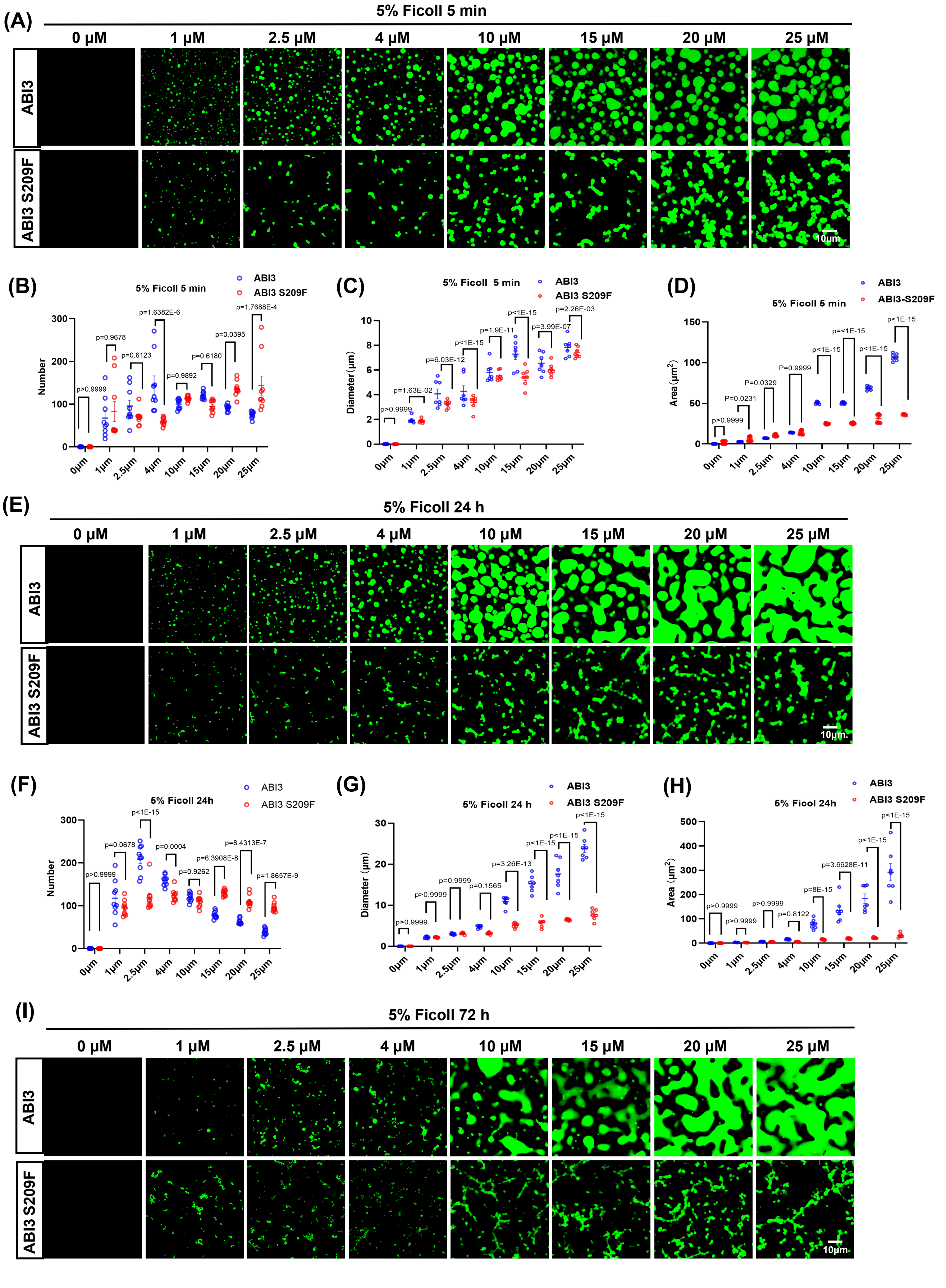
(A)
5% Ficoll 5 min
0 μM
1 μM
2.5 μM
4 μM
10 μM
15 μM
20 μM
25 μM
ABI3
ABI3 S209F
10μm
(B)
5% Ficoll 5 min
ABI3
ABI3 S209F
Number
p>0.9999
p=0.9678
p=0.6123
p=1.6382E-6
p=0.9892
p=0.6180
p=0.0395
p=1.7688E-4
0μm
1μm
2.5μm
4μm
10μm
15μm
20μm
25μm
(C)
5% Ficoll 5 min
ABI3
ABI3 S209F
Diameter (μm)
p>0.9999
p=1.63E-02
p=6.03E-12
p<1E-15
p=1.9E-11
p<1E-15
p=3.99E-07
p=2.26E-03
(D)
5% Ficoll 5 min
ABI3
ABI3-S209F
Area (μm^2)
p>0.9999
P=0.0231
P=0.0329
P=0.9999
p<1E-15
p=<1E-15
p<1E-15
p<1E-15
(E)
5% Ficoll 24 h
0 μM
1 μM
2.5 μM
4 μM
10 μM
15 μM
20 μM
25 μM
ABI3
ABI3 S209F
10μm
(F)
5% Ficoll 24h
ABI3
ABI3 S209F
Number
p>0.9999
p=0.0678
p<1E-15
p=0.0004
p=0.9262
p=6.3908E-8
p=8.4313E-7
p=1.8657E-9
(G)
5% Ficoll 24 h
ABI3
ABI3 S209F
Diameter (μm)
p>0.9999
p>0.9999
p=0.9999
p=0.1565
p=3.26E-13
p<1E-15
p<1E-15
p<1E-15
(H)
5% Ficol 24h
ABI3
ABI3 S209F
Area (μm^2)
p>0.9999
p>0.9999
p>0.9999
p=0.8122
p=8E-15
p=3.6628E-11
p<1E-15
p<1E-15
(I)
5% Ficoll 72 h
0 μM
1 μM
2.5 μM
4 μM
10 μM
15 μM
20 μM
25 μM
ABI3
ABI3 S209F
10μm

SUPPLEMENTARY FIGURE 14 ABI3 and *ABI3 S209F* exhibit distinct LLPS properties in vitro with 5% high-molecular-weight Ficoll 400; *ABI3 S209F* transitions from droplets to fibrous structures over time.

(A) Representative fluorescence images of LLPS in vitro after mixing different concentrations of ABI3 and *ABI3 S209F* with 5% Ficoll 400 for 5 min. Scale bar: 10 μm. **(B)** Analysis of phase-separated droplet counts after mixing different concentrations of ABI3 and *ABI3 S209F* with 5% Ficoll 400 for 5 min in vitro. *ABI3 S209F* formed more droplets than ABI3 at high protein concentrations (20 μM, 25 μM). **(C)** Measurement of phase-separated droplet diameters after mixing different concentrations of ABI3 and *ABI3 S209F* with 5% Ficoll 400 for 5 min in vitro. *ABI3 S209F* formed smaller droplets than ABI3. **(D)** Analysis of phase-separated droplet areas after mixing different concentrations of ABI3 and *ABI3 S209F* with 5% Ficoll 400 for 5 min in vitro. At matching concentrations, *ABI3 S209F* formed smaller droplet areas than ABI3. **(E)** Representative fluorescence images of LLPS in vitro after mixing different concentrations of ABI3 and *ABI3 S209F* with 5% Ficoll 400 for 24 h. Scale bar: 10 μm. **(F)** Analysis of phase-separated droplet counts after mixing different concentrations of ABI3 and *ABI3 S209F* with 5% Ficoll 400 for 24 h in vitro. *ABI3 S209F* formed more droplets than ABI3 at high protein concentrations (15 μM, 20 μM, 25 μM). **(G)** Measurement of phase-separated droplet diameters after mixing different concentrations of ABI3 and *ABI3 S209F* with 5% Ficoll 400 for 24 h in vitro. At matching concentrations, *ABI3 S209F* formed smaller droplets than ABI3. **(H)** Analysis of phase-separated droplet areas after mixing different concentrations of ABI3 and *ABI3 S209F* with 5% Ficoll 400 for 24 h in vitro. At matching concentrations, *ABI3 S209F* formed smaller droplet areas than ABI3. **(I)** Representative fluorescence images of LLPS in vitro after mixing different concentrations of ABI3 and *ABI3 S209F* with 5% Ficoll 400 for 72 h. With increasing protein concentration, ABI3 transitions from droplets to a gel-like structure, while *ABI3 S209F* transitions from droplets to a fibrous structure. Scale bar: 10 μm. Data presentation: Data are presented as mean ± SEM. Statistical analyses were performed using two-way ANOVA **(B–D, F-H)**.
