# Supplementary material for: Aberrant phase separation from a rare ABI3 mutation drives microglial dysfunction and Alzheimer's risk
Source: Alzheimers Dement. 2026 Sep 24;22(9):e71812. doi: 10.1002/alz.71812 (PMC13602175; doi:10.1002/alz.71812)
Supplement: Supplementary file 6 [file ALZ-22-e71812-s006.pdf]

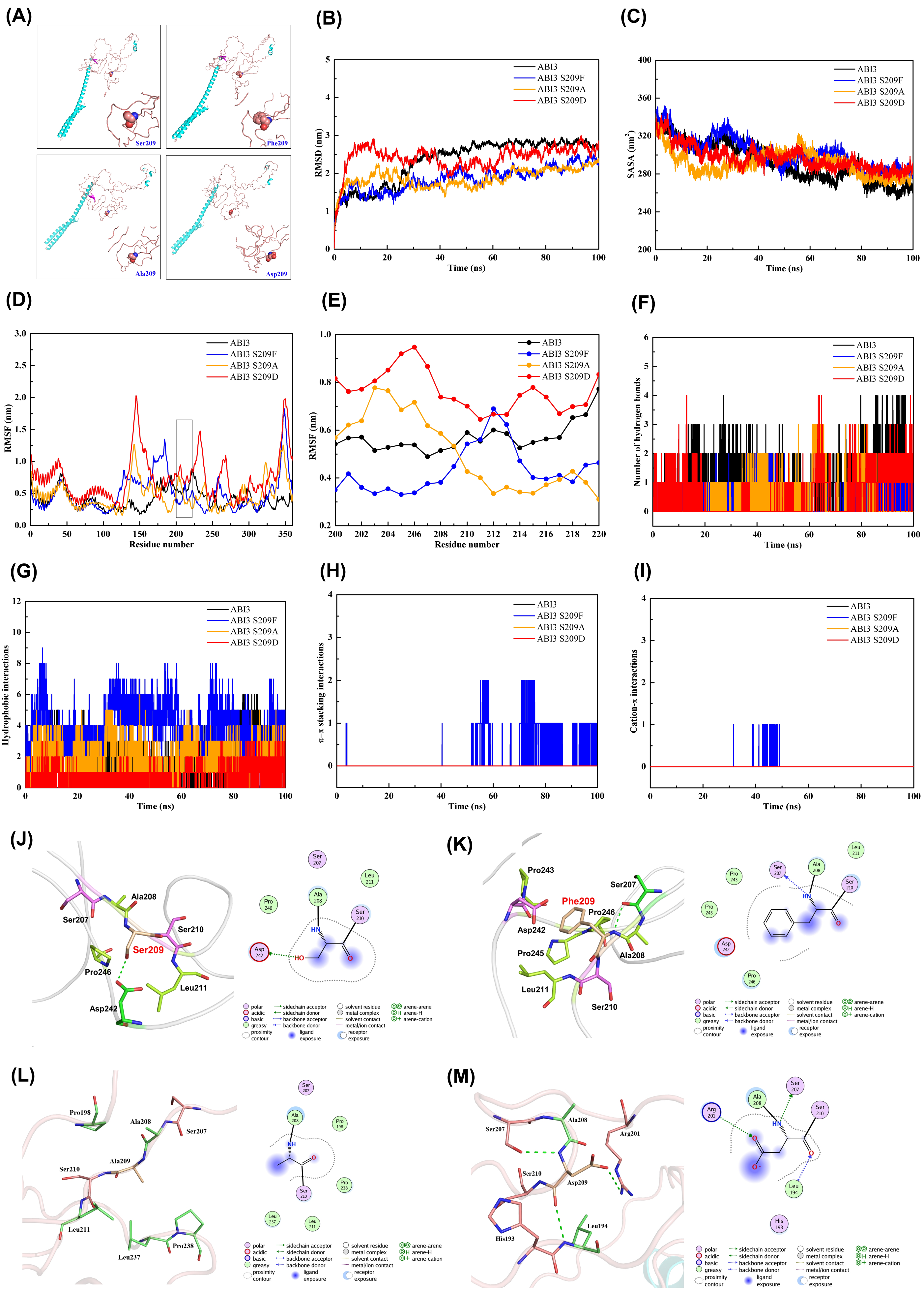

(A)
Ser209
Phe209
Ala209
Asp209
(B)
RMSD (nm)
Time (ns)
ABI3
ABI3 S209F
ABI3 S209A
ABI3 S209D
(C)
SASA (nm^2)
Time (ns)
(D)
RMSF (nm)
Residue number
(E)
RMSF (nm)
Residue number
(F)
Number of hydrogen bonds
Time (ns)
(G)
Hydrophobic interactions
Time (ns)
(H)
π-π stacking interactions
Time (ns)
(I)
Cation-π interactions
Time (ns)
(J)
Ser207
Ala208
Ser209
Ser210
Pro246
Leu211
Asp242
(K)
Pro243
Ser207
Phe209
Pro246
Asp242
Pro245
Ala208
Leu211
Ser210
(L)
Pro198
Ala208
Ser207
Ala209
Ser210
Leu211
Leu237
Pro238
(M)
Ser207
Ala208
Arg201
Ser210
Asp209
His193
Leu194
polar
acidic
basic
greasy
proximity contour
sidechain acceptor
sidechain donor
backbone acceptor
backbone donor
ligand exposure
solvent residue
metal complex
solvent contact
metal/ion contact
receptor exposure
arene-arene
arene-H
arene-cation

SUPPLEMENTARY FIGURE 11 Molecular dynamics simulations predict the impact of *ABI3 S209F* mutation on protein structure.

(A) Structural models of ABI3*, ABI3 S209F*, *ABI3 S209A*, and *ABI3 S209D* variants. **(B)** The variation of RMSD values with simulation time in ABI3*, ABI3 S209F*, *ABI3 S209A*, and *ABI3 S209D* systems. **(C)** The variation of SASA values with simulation time in ABI3*, ABI3 S209F*, *ABI3 S209A*, and *ABI3 S209D* systems. **(D)** Distribution of RMSF of protein amino acids in ABI3*, ABI3 S209F*, *ABI3 S209A*, and *ABI3 S209D* systems. **(E)** Magnified RMSF distribution of protein amino acids in ABI3*, ABI3 S209F*, *ABI3 S209A*, and *ABI3 S209D* systems. **(F)** Hydrogen bonding between residue 209 and surrounding amino acids is increased in *ABI3 S209D*. **(G)** Hydrophobic interactions between residue 209 and surrounding amino acids are increased in *ABI3 S209F* and *ABI3 S209A*. **(H)** Residue 209 forms π-π interactions with surrounding amino acids in *ABI3 S209F*. **(I)** Residue 209 forms cation-π interactions with surrounding amino acids in *ABI3 S209F*. **(J)** 3D and 2D binding patterns of Ser209 with surrounding amino acids in the ABI3 system. **(K)** 3D and 2D binding patterns of Phe209 with surrounding amino acids in the *ABI3 S209F* system. **(L)** 3D and 2D binding patterns of Ala209 with surrounding amino acids in the ABI3 *S209A* system. **(M)** 3D and 2D binding patterns of Asp209 with surrounding amino acids in the ABI3 *S209D* system.
