# Supplementary material for: Aberrant phase separation from a rare ABI3 mutation drives microglial dysfunction and Alzheimer's risk
Source: Alzheimers Dement. 2026 Sep 24;22(9):e71812. doi: 10.1002/alz.71812 (PMC13602175; doi:10.1002/alz.71812)
Supplement: Supplementary file 8 [file ALZ-22-e71812-s008.pdf]

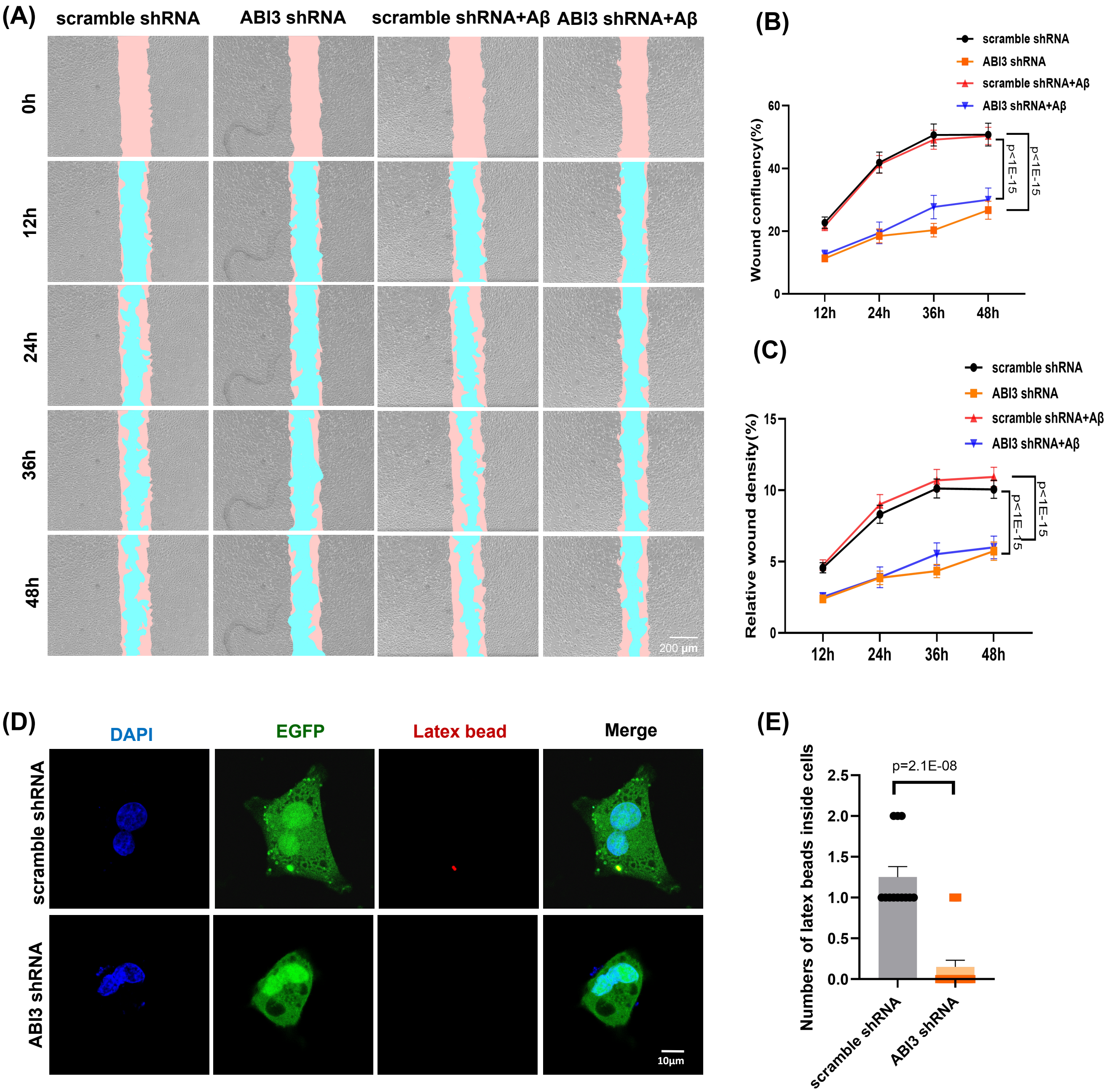

SUPPLEMENTARY FIGURE 8 ***ABI3*** **knockdown inhibits the migration and phagocytosis of HMC3 cells.**

(A) Representative images of scratch areas in HMC3 cells overexpressing *ABI3* shRNA or scramble shRNA, with or without 100 nM Aβ stimulation, at 12 h, 24 h, 36 h, and 48 h. Scale bar: 200 μm. **(B)-(C)** Statistical analysis of wound healing rates (**B**) and relative wound density (**C**) in HMC3 cells overexpressing *ABI3* shRNA or scramble shRNA, with or without 100 nM Aβ stimulation, at 12 h, 24 h, 36 h, and 48 h. *ABI3* knockdown significantly reduced wound healing rates under both unstimulated and 100 nM Aβ-stimulated conditions. **(D)** *ABI3* shRNA cells exhibited a reduced uptake of red fluorescent latex beads compared to scramble shRNA control cells. Scale bar: 10 μm. **(E)** Statistical graph showing the number of fluorescent red latex beads phagocytosed by HMC3 cells in each group. Data are presented as mean±SEM. Statistical analyses were performed using two-way ANOVA **(B, C)** or unpaired Student's t-test **(E).**
