# Supplementary material for: Aberrant phase separation from a rare ABI3 mutation drives microglial dysfunction and Alzheimer's risk
Source: Alzheimers Dement. 2026 Sep 24;22(9):e71812. doi: 10.1002/alz.71812 (PMC13602175; doi:10.1002/alz.71812)
Supplement: Supplementary file 9 [file ALZ-22-e71812-s009.pdf]

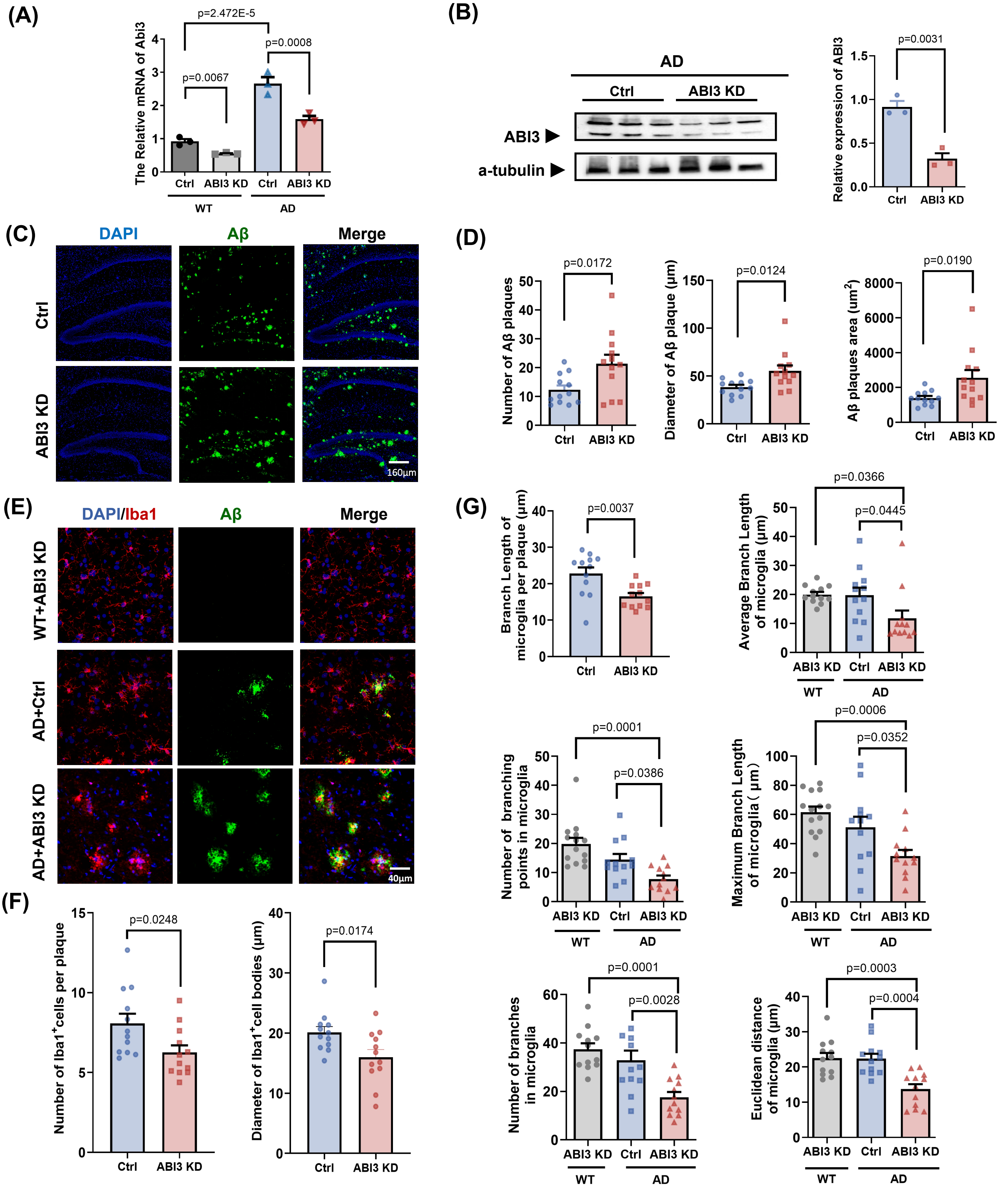

(A)
p=2.472E-5
p=0.0008
p=0.0067
The Relative mRNA of Abi3
Ctrl
ABI3 KD
Ctrl
ABI3 KD
WT
AD
(B)
AD
Ctrl
ABI3 KD
ABI3
a-tubulin
p=0.0031
Relative expression of ABI3
Ctrl
ABI3 KD
(C)
DAPI
Aβ
Merge
Ctrl
ABI3 KD
160μm
(D)
p=0.0172
Number of Aβ plaques
p=0.0124
Diameter of Aβ plaque (μm)
p=0.0190
Aβ plaques area (um²)
Ctrl
ABI3 KD
(E)
DAPI/Iba1
Aβ
Merge
WT+ABI3 KD
AD+Ctrl
AD+ABI3 KD
40μm
(F)
p=0.0248
Number of Iba1⁺cells per plaque
p=0.0174
Diameter of Iba1⁺cell bodies (μm)
Ctrl
ABI3 KD
(G)
p=0.0037
Branch Length of microglia per plaque (μm)
p=0.0366
p=0.0445
Average Branch Length of microglia (μm)
p=0.0001
p=0.0386
Number of branching points in microglia
p=0.0006
p=0.0352
Maximum Branch Length of microglia (μm)
p=0.0001
p=0.0028
Number of branches in microglia
p=0.0003
p=0.0004
Euclidean distance of microglia (μm)
ABI3 KD
Ctrl
ABI3 KD
WT
AD

SUPPLEMENTARY FIGURE 3 Microglia-specific ABI3 knockdown accelerates amyloid pathology in AD mice.

(A) *Abi3* mRNA expression levels in WT+Ctrl, WT+ABI3 KD, AD+Ctrl, and AD+ABI3 KD. (n=3/group). Compared with the AD+Ctrl group. **(B)** ABI3 protein expression levels in AD+Ctrl, and AD+ABI3 KD (n=3/group). Compared with the AD+Ctrl group. (n=3/group). **(C-D)** Aβ plaque deposition in the hippocampus of AD+Ctrl and AD+ABI3 KD mice. Representative confocal images (**C**) and quantification of Aβ deposition number, diameter, and area (**D**). Scale bar: 160 μm. (n=12/group). **(E)-(G)** Morphology of microglia surrounding Aβ plaques in WT+ABI3 KD, AD+Ctrl, and AD+ABI3 KD mice.(n=12/group). Representative confocal images (**E**), Quantification of microglial number and diameter. Scale bar: 40 μm (**F**); Quantification of branch length and number of plaque microglia (**G**). (n=12/group). Data are presented as mean±SEM. Statistical analyses were performed using unpaired Student's t-test **(B, D, F, G)** and one-way ANOVA **(A, G)**.
