# Supplementary material for: Aberrant phase separation from a rare ABI3 mutation drives microglial dysfunction and Alzheimer's risk
Source: Alzheimers Dement. 2026 Sep 24;22(9):e71812. doi: 10.1002/alz.71812 (PMC13602175; doi:10.1002/alz.71812)
Supplement: Supplementary file 10 [file ALZ-22-e71812-s010.pdf]

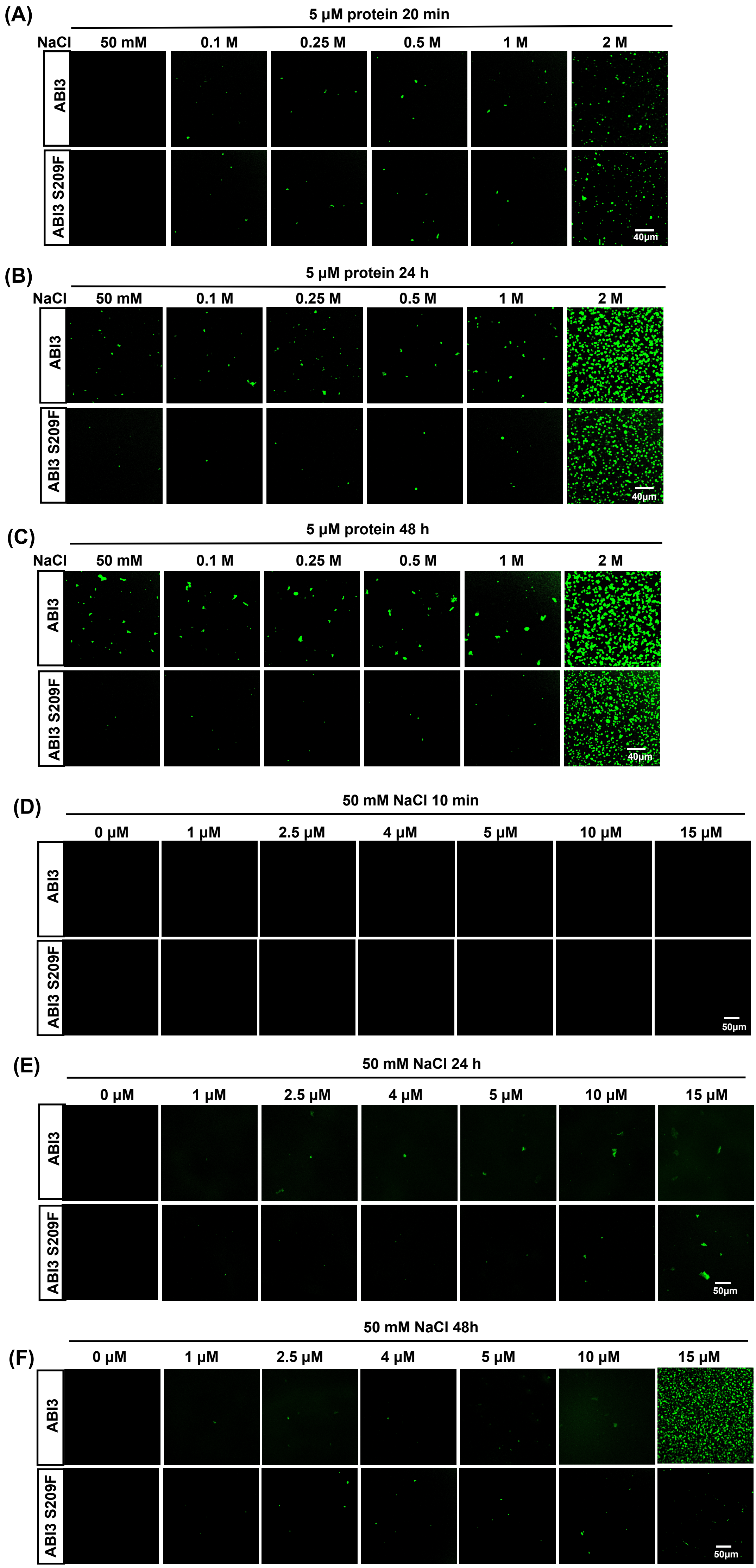
(A)
5 μM protein 20 min
NaCl
50 mM
0.1 M
0.25 M
0.5 M
1 M
2 M
ABI3
ABI3 S209F
40μm
(B)
5 μM protein 24 h
NaCl
50 mM
0.1 M
0.25 M
0.5 M
1 M
2 M
ABI3
ABI3 S209F
40μm
(C)
5 μM protein 48 h
NaCl
50 mM
0.1 M
0.25 M
0.5 M
1 M
2 M
ABI3
ABI3 S209F
40μm
(D)
50 mM NaCl 10 min
0 μM
1 μM
2.5 μM
4 μM
5 μM
10 μM
15 μM
ABI3
ABI3 S209F
50μm
(E)
50 mM NaCl 24 h
0 μM
1 μM
2.5 μM
4 μM
5 μM
10 μM
15 μM
ABI3
ABI3 S209F
50μm
(F)
50 mM NaCl 48h
0 μM
1 μM
2.5 μM
4 μM
5 μM
10 μM
15 μM
ABI3
ABI3 S209F
50μm

SUPPLEMENTARY FIGURE 16 ***ABI3 S209F*** **forms smaller and fewer droplets than ABI3 when incubated with NaCl under identical conditions.**

(A) Representative fluorescence images of liquid-liquid phase separation (LLPS) in vitro after mixing 5 μM ABI3 or *ABI3 S209F* with varying NaCl concentrations for 20 min. Scale bar: 40 μm. **(B)** Representative fluorescence images of LLPS in vitro after mixing 5 μM ABI3 or *ABI3 S209F* with varying NaCl concentrations for 24 h. Scale bar: 40 μm. **(C)** Representative fluorescence images of LLPS in vitro after mixing 5 μM ABI3 or *ABI3 S209F* with varying NaCl concentrations for 48 h. Scale bar: 40 μm. **(D)** Representative fluorescence images showing no phase-separated droplets formed in vitro after mixing different concentrations of ABI3 or *ABI3 S209F* with 50 mM NaCl for 10 min. Scale bar: 50 μm. **(E)** Representative fluorescence images of LLPS droplets formed in vitro after mixing different concentrations of ABI3 or *ABI3 S209F* with 50 mM NaCl for 24 h. Scale bar: 50 μm. **(F)** Representative fluorescence images of LLPS droplets formed in vitro after mixing different concentrations of ABI3 or *ABI3 S209F* with 50 mM NaCl for 48 h. Scale bar: 50 μm.
