# Supplementary material for: Aberrant phase separation from a rare ABI3 mutation drives microglial dysfunction and Alzheimer's risk
Source: Alzheimers Dement. 2026 Sep 24;22(9):e71812. doi: 10.1002/alz.71812 (PMC13602175; doi:10.1002/alz.71812)
Supplement: Supplementary file 11 [file ALZ-22-e71812-s011.pdf]

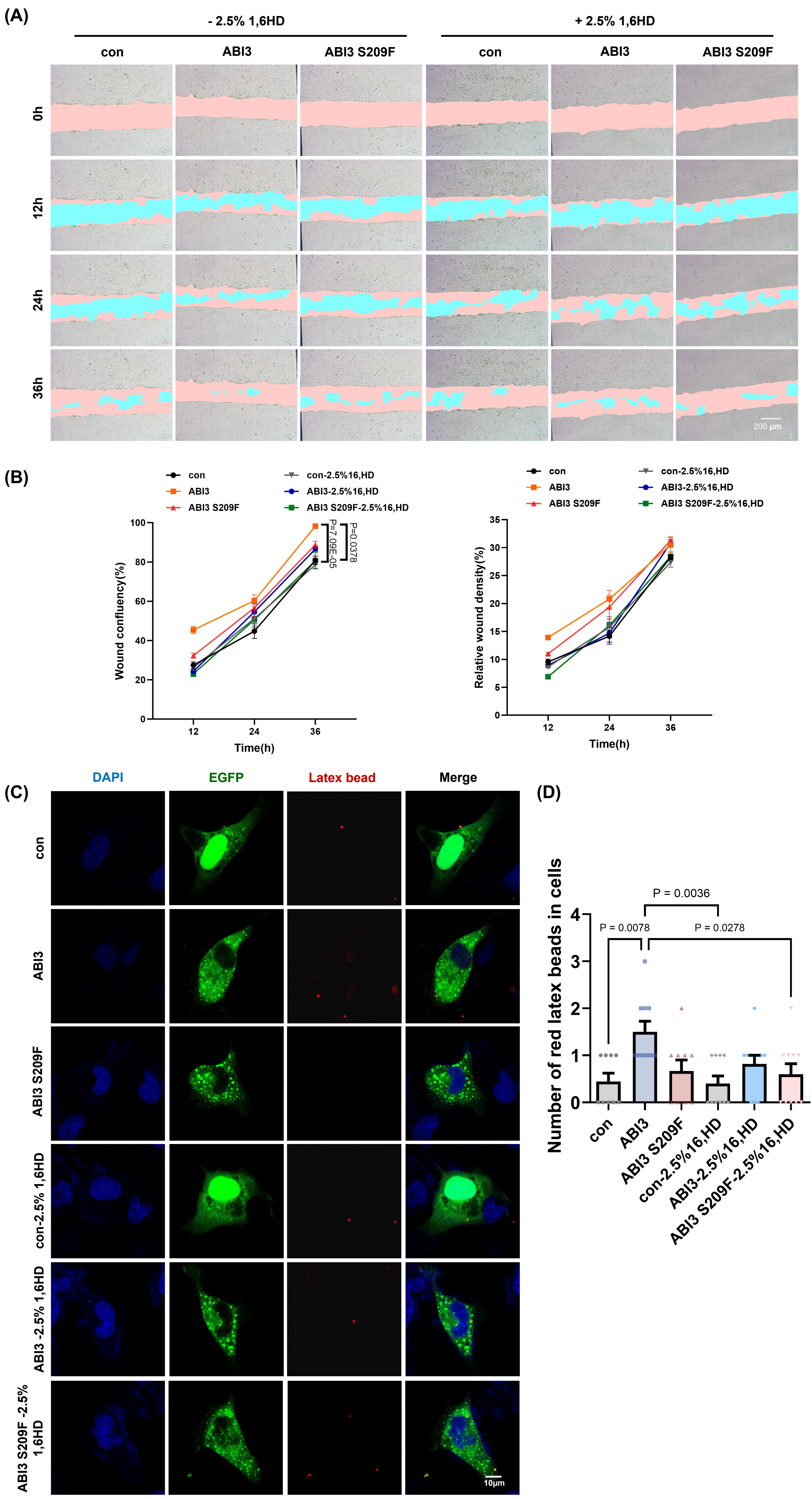

(A)
- 2.5% 1,6HD
+ 2.5% 1,6HD
con
ABI3
ABI3 S209F
con
ABI3
ABI3 S209F
0h
12h
24h
36h
200 μm
(B)
con
ABI3
ABI3 S209F
con-2.5%16,HD
ABI3-2.5%16,HD
ABI3 S209F-2.5%16,HD
Wound confluency(%)
Time(h)
P=0.0378
P=7.09E-05
Relative wound density(%)
Time(h)
(C)
DAPI
EGFP
Latex bead
Merge
con
ABI3
ABI3 S209F
con-2.5% 1,6HD
ABI3 -2.5% 1,6HD
ABI3 S209F -2.5% 1,6HD
10μm
(D)
Number of red latex beads in cells
P = 0.0036
P = 0.0078
P = 0.0278
con
ABI3
ABI3 S209F
con-2.5%16,HD
ABI3-2.5%16,HD
ABI3 S209F-2.5%16,HD

SUPPLEMENTARY FIGURE 20 2.5% 1,6-HD inhibits ABI3-mediated migration and phagocytosis in HMC3 cells by disrupting the phase separation ability of ABI3.

(**A**) Representative images of scratch areas in the ABI3 or *ABI3 S209F* group with or without 2.5% 1,6-HD treatment of HMC3 cells at 12 h, 24 h, and 36 h. Scale bar: 200 μm. **(B)** Quantification of wound confluency and relative wound density in scratch areas from scratch assays in HMC3 cells. (**C**) Representative images of phagocytosis in the ABI3 or *ABI3 S209F* group with or without 2.5% 1,6-HD treatment of HMC3 cells at 37 ° C for 5 min prior to the assay. Scale bar: 10 μm. **(D)** Statistical graph showing the number of fluorescent red latex beads phagocytosed by HMC3 cells in each group. Data are presented as mean ± SEM. Statistical analyses were performed using one-way ANOVA (**D**) and two-way ANOVA **(B)**.
