# Supplementary material for: Aberrant phase separation from a rare ABI3 mutation drives microglial dysfunction and Alzheimer's risk
Source: Alzheimers Dement. 2026 Sep 24;22(9):e71812. doi: 10.1002/alz.71812 (PMC13602175; doi:10.1002/alz.71812)
Supplement: Supplementary file 12 [file ALZ-22-e71812-s012.pdf]

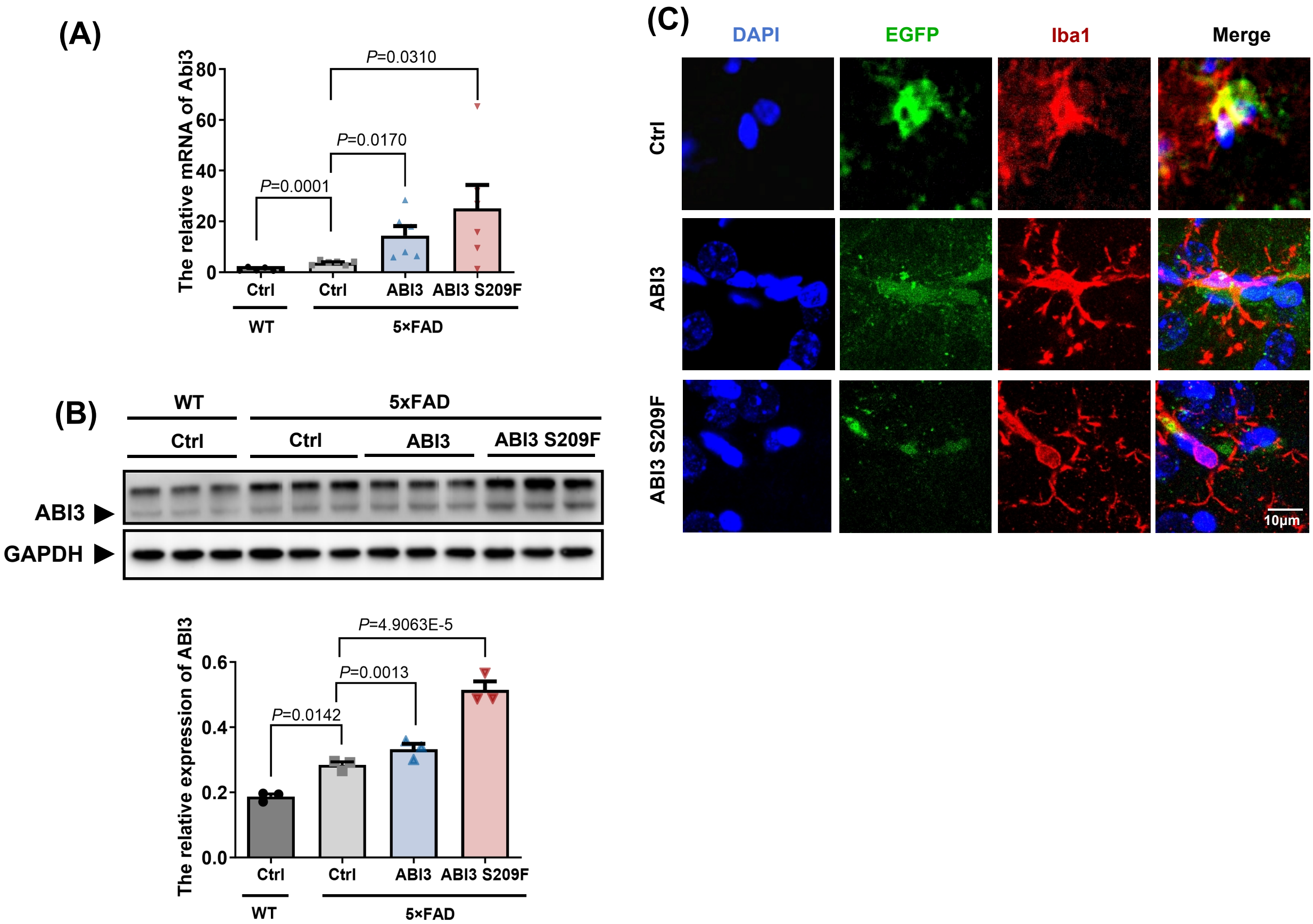

SUPPLEMENTARY FIGURE 5 Overexpression of EGFP-fusion proteins in 5×FAD mice was confirmed.

(A) QPCR assay examined *Abi3* mRNA levels in WT+Ctrl (n = 6), 5×FAD+Ctrl (n = 6), 5×FAD+ABI3 (n = 6), and 5×FAD+*ABI3-S209F* (n = 6) groups. **(B)** Western blot examined ABI3 protein expression in WT+Ctrl (n=3), 5×FAD+Ctrl (n=3), 5×FAD+ABI3 (n=3), and 5×FAD+*ABI3-S209F* (n=3) groups. **(C)** Confocal imaging examined the construction of microglia-specific ABI3 and *ABI3-S209F* overexpression in the hippocampus of 5×FAD mice. Scale bar: 10 μm. Data is presented as mean ± SEM. Statistical analysis was performed using one-way ANOVA **(A-B)**.
