# Supplementary material for: Aberrant phase separation from a rare ABI3 mutation drives microglial dysfunction and Alzheimer's risk
Source: Alzheimers Dement. 2026 Sep 24;22(9):e71812. doi: 10.1002/alz.71812 (PMC13602175; doi:10.1002/alz.71812)
Supplement: Supplementary file 15 [file ALZ-22-e71812-s015.pdf]

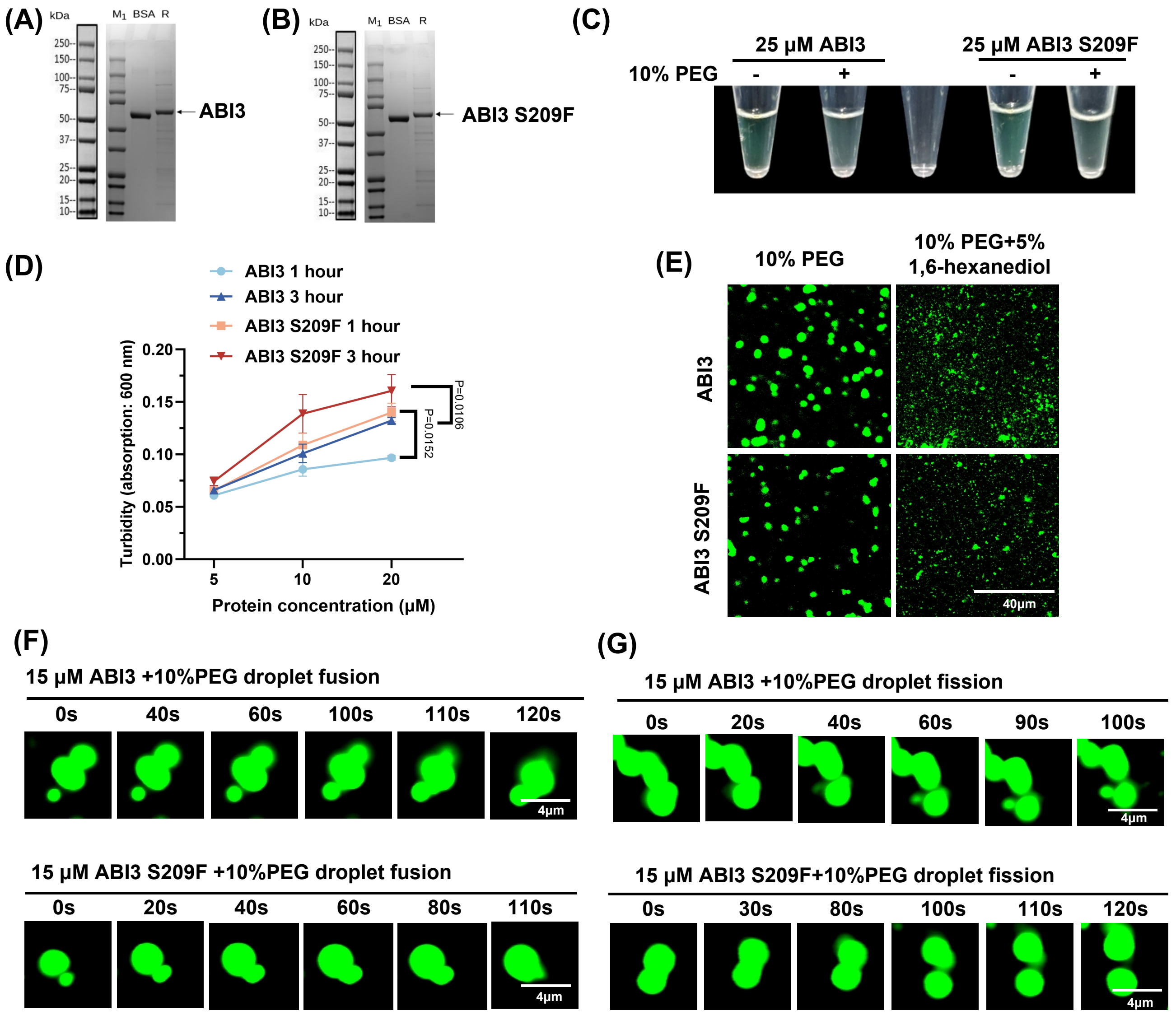

SUPPLEMENTARY FIGURE 12 Validation of ABI3 and *ABI3 S209F* phase separation properties in vitro.

(A) Purified recombinant ABI3 protein. Successfully isolated in vitro-purified recombinant ABI3 protein. **(B)** Successfully isolated in vitro-purified recombinant *ABI3 S209F* protein. **(C)** Mixtures of 25 μM ABI3 or *ABI3 S209F* with 10% PEG 8000 appeared turbid in vitro. **(D)** Turbidity assay measuring changes in turbidity after incubating varying concentrations of ABI3 or *ABI3 S209F* with 10% PEG 8000 for 1 h or 3 h in vitro. **(E)** Dissolution of droplet-like condensates (formed by mixing 15 μM ABI3 or *ABI3 S209F* with 10% PEG 8000 for 5 min in vitro) was significantly increased after 5% 1,6-HD treatment for 5 min. Scale bar: 40 μm. **(F)-(G)** Droplets formed by 15 μM ABI3 or *ABI3 S209F* (mixed with 10% PEG 8000 for 10 min in vitro) could fuse with adjacent droplets (**F**) and separate from one another (**G**). Scale bar: 4 μm. Data is presented as mean±SEM. Statistical analysis was performed using two-way ANOVA **(D).**
