# Supplementary material for: Aberrant phase separation from a rare ABI3 mutation drives microglial dysfunction and Alzheimer's risk
Source: Alzheimers Dement. 2026 Sep 24;22(9):e71812. doi: 10.1002/alz.71812 (PMC13602175; doi:10.1002/alz.71812)
Supplement: Supplementary file 16 [file ALZ-22-e71812-s016.pdf]

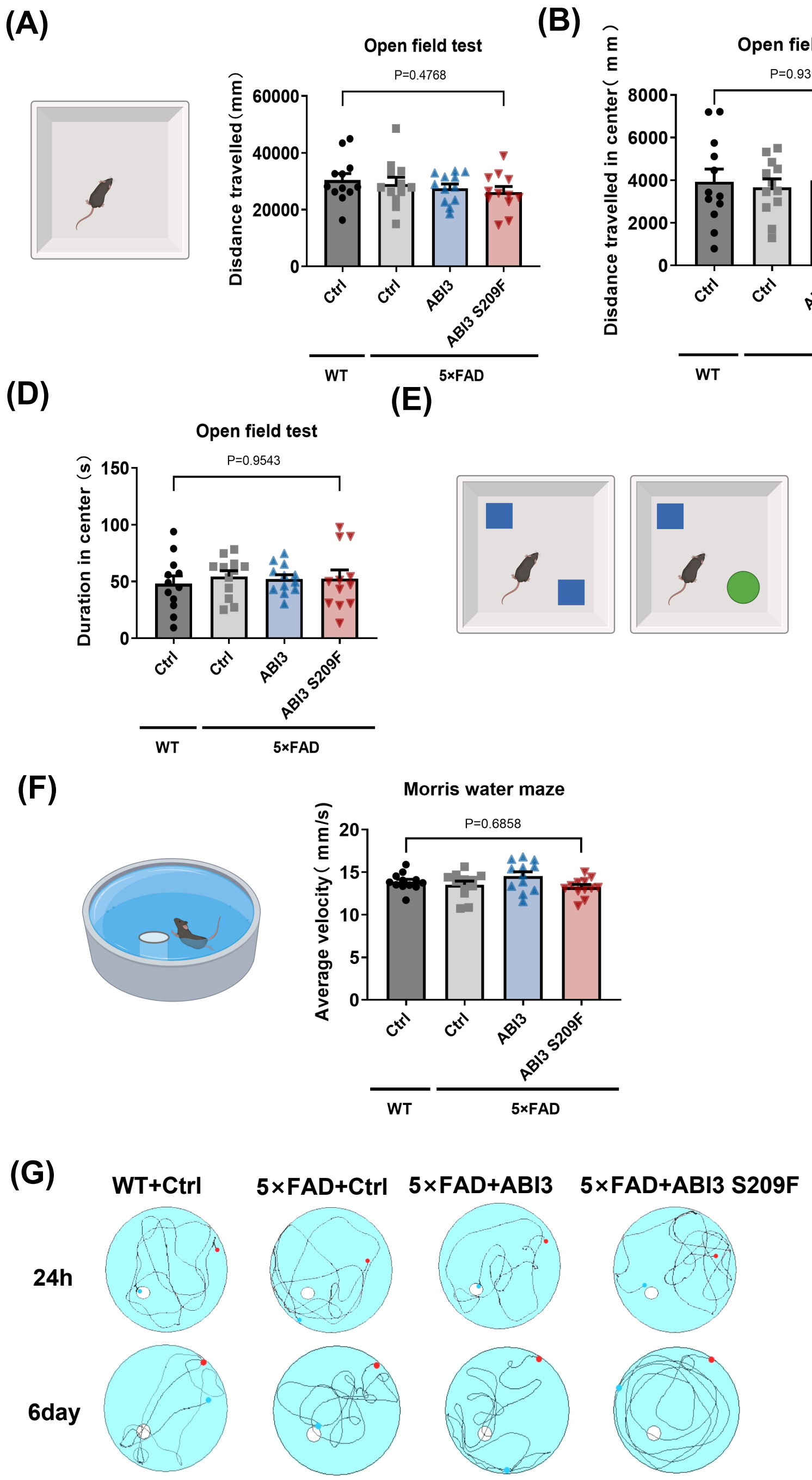

(A)
Open field test
P=0.4768
Disdance travelled (mm)
60000
40000
20000
0
Ctrl
Ctrl
ABI3
ABI3 S209F
WT
5×FAD
(B)
Open field test
P=0.9399
Disdance travelled in center ( m m )
8000
6000
4000
2000
0
Ctrl
Ctrl
ABI3
ABI3 S209F
WT
5×FAD
(C)
Open field test
P=0.5420
Velocity in center ( mm/s )
150
100
50
0
Ctrl
Ctrl
ABI3
ABI3 S209F
WT
5×FAD
(D)
Open field test
P=0.9543
Duration in center (s)
150
100
50
0
Ctrl
Ctrl
ABI3
ABI3 S209F
WT
5×FAD
(E)
Novel Object Recognition
P=0.9001
Recognition index(%)
1.5
1.0
0.5
0.0
Ctrl
Ctrl
ABI3
ABI3 S209F
WT
5×FAD
(F)
Morris water maze
P=0.6858
Average velocity ( mm/s)
20
15
10
5
0
Ctrl
Ctrl
ABI3
ABI3 S209F
WT
5×FAD
(G)
WT+Ctrl
5×FAD+Ctrl
5×FAD+ABI3
5×FAD+ABI3 S209F
24h
6day

**SUPPLEMENTARY FIGURE 6 Behavior assay of 5×FAD mice with microglia-specific ABI3/*ABI3 S209F* overexpression in open field tests, novel object recognition, and Morris water maze.**

**(A)** No significant difference in distance traveled by WT+Ctrl, 5×FAD+Ctrl, 5×FAD+ABI3, and 5×FAD+*ABI3-S209F* mice (n=12/group). **(B)** No significant difference in distance traveled in the center zone by WT+Ctrl, 5×FAD+Ctrl, 5×FAD+ABI3, and 5×FAD+*ABI3-S209F* mice (n=12/group). **(C)** No significant difference in velocity in the center zone of WT+Ctrl, 5×FAD+Ctrl, 5×FAD+ABI3, and 5×FAD+*ABI3-S209F* mice (n=12/group). **(D)** No significant difference in duration spent in the center zone by WT+Ctrl, 5×FAD+Ctrl, 5×FAD+ABI3 , and 5×FAD+*ABI3-S209F* mice (n=12/group). **(E)** No significant difference in the novel object recognition coefficient among WT+Ctrl, 5×FAD+Ctrl, 5×FAD+ABI3, and 5×FAD+*ABI3-S209F* mice (n=12/group). **(F)** Average swimming velocity in all groups, with no significant differences observed (n=12/group). **(G)** Representative trajectories of mice in all groups after 24 h and 6 days of training in the Morris water maze. Group definitions: WT+Ctrl: C57BL/6 mice injected with microglia-specific EGFP-CON AAV. 5×FAD+Ctrl: 5×FAD mice injected with microglia-specific EGFP-CON AAV. 5×FAD+ABI3: 5×FAD mice injected with microglia-specific EGFP-ABI3 AAV. 5×FAD+*ABI3-S209F*: 5×FAD mice injected with microglia-specific EGFP-*ABI3 S209F* AAV. Data are presented as mean±SEM. Statistical analyses were performed using one-way ANOVA **(A-F)**.
