# Supplementary material for: Aberrant phase separation from a rare ABI3 mutation drives microglial dysfunction and Alzheimer's risk
Source: Alzheimers Dement. 2026 Sep 24;22(9):e71812. doi: 10.1002/alz.71812 (PMC13602175; doi:10.1002/alz.71812)
Supplement: Supplementary file 18 [file ALZ-22-e71812-s018.pdf]

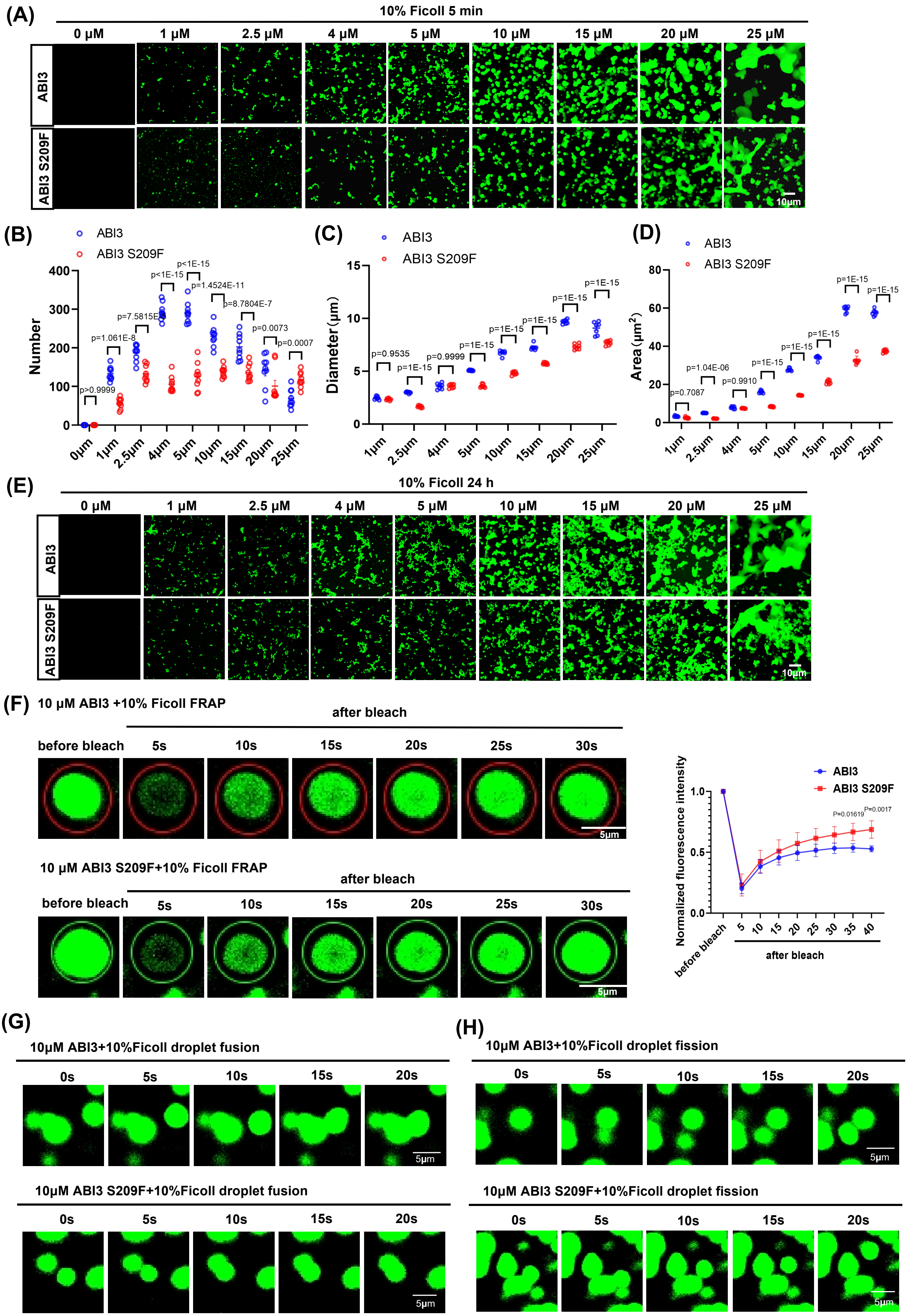

(A)
10% Ficoll 5 min
0 μM
1 μM
2.5 μM
4 μM
5 μM
10 μM
15 μM
20 μM
25 μM
ABI3
ABI3 S209F
10μm
(B)
ABI3
ABI3 S209F
Number
(C)
Diameter (μm)
(D)
Area (μm²)
(E)
10% Ficoll 24 h
(F)
10 μM ABI3 +10% Ficoll FRAP
after bleach
before bleach
5s
10s
15s
20s
25s
30s
5μm
10 μM ABI3 S209F+10% Ficoll FRAP
Normalized fluorescence intensity
P=0.01619
P=0.0017
(G)
10μM ABI3+10%Ficoll droplet fusion
0s
5s
10s
15s
20s
10μM ABI3 S209F+10%Ficoll droplet fusion
(H)
10μM ABI3+10%Ficoll droplet fission
10μM ABI3 S209F+10%Ficoll droplet fission

SUPPLEMENTARY FIGURE 15 ABI3 and *ABI3 S209F* exhibit distinct LLPS properties in vitro with 10% high-molecular-weight Ficoll 400; *ABI3 S209F* transitions from droplets to fibrous structures over time.

(A) Representative fluorescence images of LLPS in vitro after mixing different concentrations of ABI3 and *ABI3 S209F* with 10% Ficoll 400 for 5 min. Scale bar: 10 μm. **(B)-(D)** Measurement of phase-separated droplet counts **(B)**, diameters **(C),** and areas **(D)** after mixing different concentrations of ABI3 and *ABI3 S209F* with 10% Ficoll 400 for 5 min in vitro. At matching concentrations, *ABI3 S209F* formed fewer and smaller droplets than ABI3. **(E)** Representative fluorescence images of LLPS in vitro after mixing different concentrations of ABI3 and *ABI3 S209F* with 10% Ficoll 400 for 24 h. With increasing protein concentration, both ABI3 and *ABI3 S209F* transition from droplets to a fibrous structure. Scale bar: 10 μm. **(F)** The fluorescence intensity of the droplet-like aggregates formed by the mixture of 10 μM ABI3 and 10 μM *ABI3 S209F* proteins with Ficoll 400 in vitro for 5 minutes rapidly recovered in the FRAP experiment. Compared with ABI3 protein, the fluorescence of *ABI3 S209F* protein recovered more quickly after photobleaching. Scale bar: 5 μm. **(G)-(H)** In vitro, the droplets formed by 10 μM ABI3 or 10 μM *ABI3 S209F* mixed with 10% Ficoll 400 for 5 min could fuse with neighboring droplets (**G**) and separate from each other (**H**). Scale bar: 5 μm. Statistical analyses were performed using two-way ANOVA **(B–D, F)**.
