# Supplementary material for: Aberrant phase separation from a rare ABI3 mutation drives microglial dysfunction and Alzheimer's risk
Source: Alzheimers Dement. 2026 Sep 24;22(9):e71812. doi: 10.1002/alz.71812 (PMC13602175; doi:10.1002/alz.71812)
Supplement: Supplementary file 19 [file ALZ-22-e71812-s019.pdf]

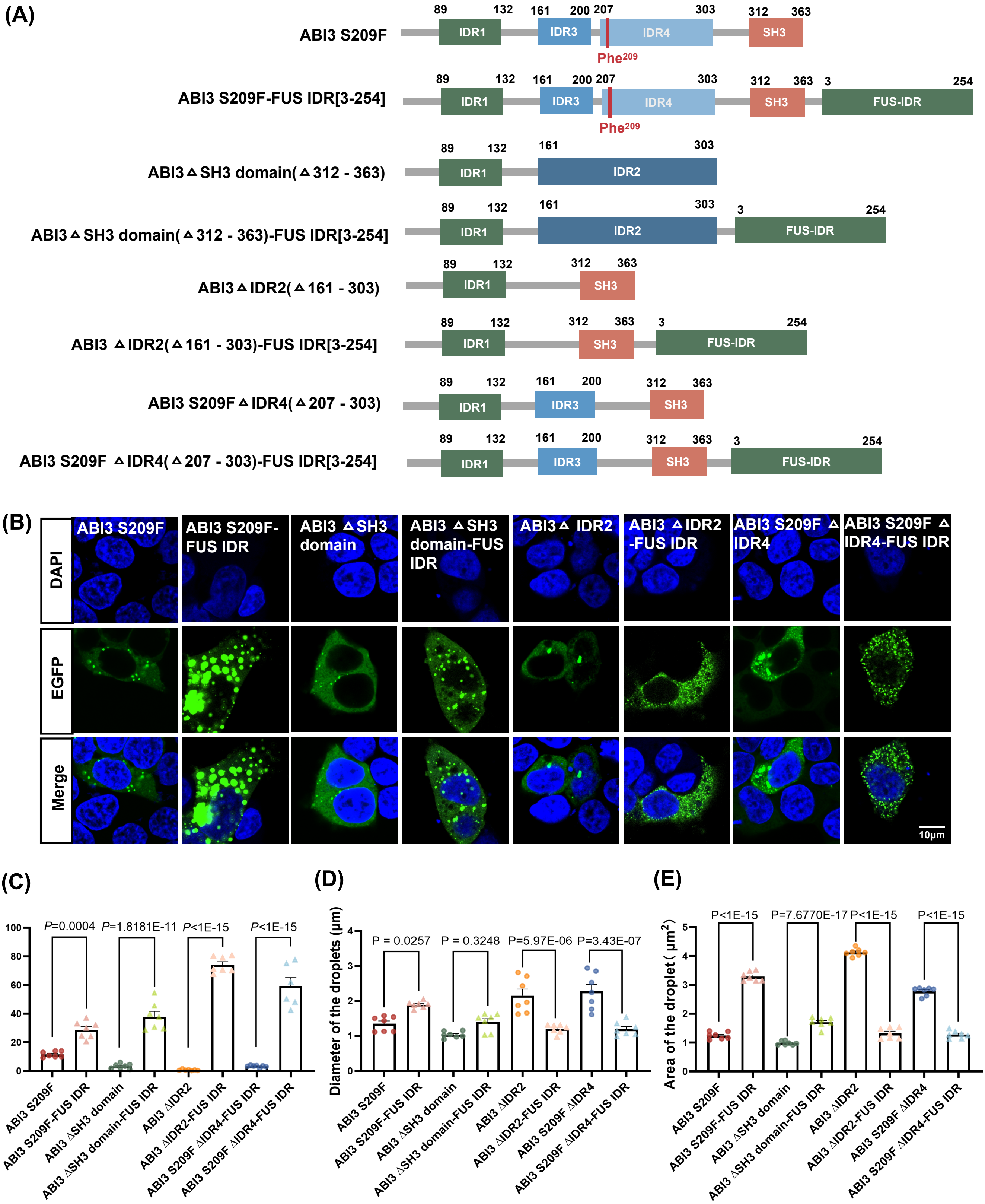

(A)
ABI3 S209F
ABI3 S209F-FUS IDR[3-254]
ABI3 △ SH3 domain(△312 - 363)
ABI3 △ SH3 domain(△312 - 363)-FUS IDR[3-254]
ABI3 △ IDR2(△161 - 303)
ABI3 △ IDR2(△161 - 303)-FUS IDR[3-254]
ABI3 S209F △ IDR4(△207 - 303)
ABI3 S209F △ IDR4(△207 - 303)-FUS IDR[3-254]
IDR1
IDR2
IDR3
IDR4
SH3
FUS-IDR
Phe209
(B)
DAPI
EGFP
Merge
ABI3 S209F
ABI3 S209F-FUS IDR
ABI3 △SH3 domain
ABI3 △SH3 domain-FUS IDR
ABI3 △ IDR2
ABI3 △IDR2 -FUS IDR
ABI3 S209F △ IDR4
ABI3 S209F △ IDR4-FUS IDR
10μm
(C)
Number of the droplets
P=0.0004
P=1.8181E-11
P<1E-15
P<1E-15
(D)
Diameter of the droplets (μm)
P = 0.0257
P = 0.3248
P=5.97E-06
P=3.43E-07
(E)
Area of the droplet (μm²)
P<1E-15
P=7.6770E-17
P<1E-15
P<1E-15
ABI3 S209F
ABI3 S209F-FUS IDR
ABI3 ΔSH3 domain
ABI3 ΔSH3 domain-FUS IDR
ABI3 ΔIDR2
ABI3 ΔIDR2-FUS IDR
ABI3 S209F ΔIDR4
ABI3 S209F ΔIDR4-FUS IDR

SUPPLEMENTARY FIGURE 18 FUS IDR linkage reverses the effects of key IDR/structural domain deletions on LLPS in ABI3 and *ABI3 S209F*.

(A) Schematic of key IDR (intrinsically disordered region) and structural domain deletions in *ABI3 S209F* and ABI3, and their FUS IDR-linked variants. **(B)** Laser confocal imaging of cytoplasmic droplet aggregates in HEK 293T cells 24 h after transfection with the indicated plasmids. Scale bar: 10 μm. **(C)** Statistical analysis of droplet numbers: FUS IDR-expressing variants of *ABI3 S209F* or ABI3 (with key IDR/domain deletions) showed significantly increased intracellular LLPS droplet counts compared to their non-FUS IDR counterparts. **(D)** Statistical analysis of droplet diameters: FUS IDR-expressing variants of *ABI3 S209F* or ABI3 (with key IDR/domain deletions) showed significantly increased intracellular LLPS droplet diameters compared to their non-FUS IDR counterparts except ABI3 ΔIDR2-FUS IDR and *ABI3 S209F* ΔIDR4-FUS IDR. **(E)** Statistical analysis of droplet areas: FUS IDR-expressing variants of *ABI3 S209F* or ABI3 (with key IDR/domain deletions) showed significantly increased intracellular LLPS droplet areas compared to their truncated, non-FUS IDR counterparts except ABI3 ΔIDR2-FUS IDR and *ABI3 S209F* ΔIDR4-FUS IDR. Data presentation: Data are presented as mean ± SEM. Statistical analyses were performed using one-way ANOVA **(C-E)**.
