# Supplementary material for: Aberrant phase separation from a rare ABI3 mutation drives microglial dysfunction and Alzheimer's risk
Source: Alzheimers Dement. 2026 Sep 24;22(9):e71812. doi: 10.1002/alz.71812 (PMC13602175; doi:10.1002/alz.71812)
Supplement: Supplementary file 23 [file ALZ-22-e71812-s023.pdf]

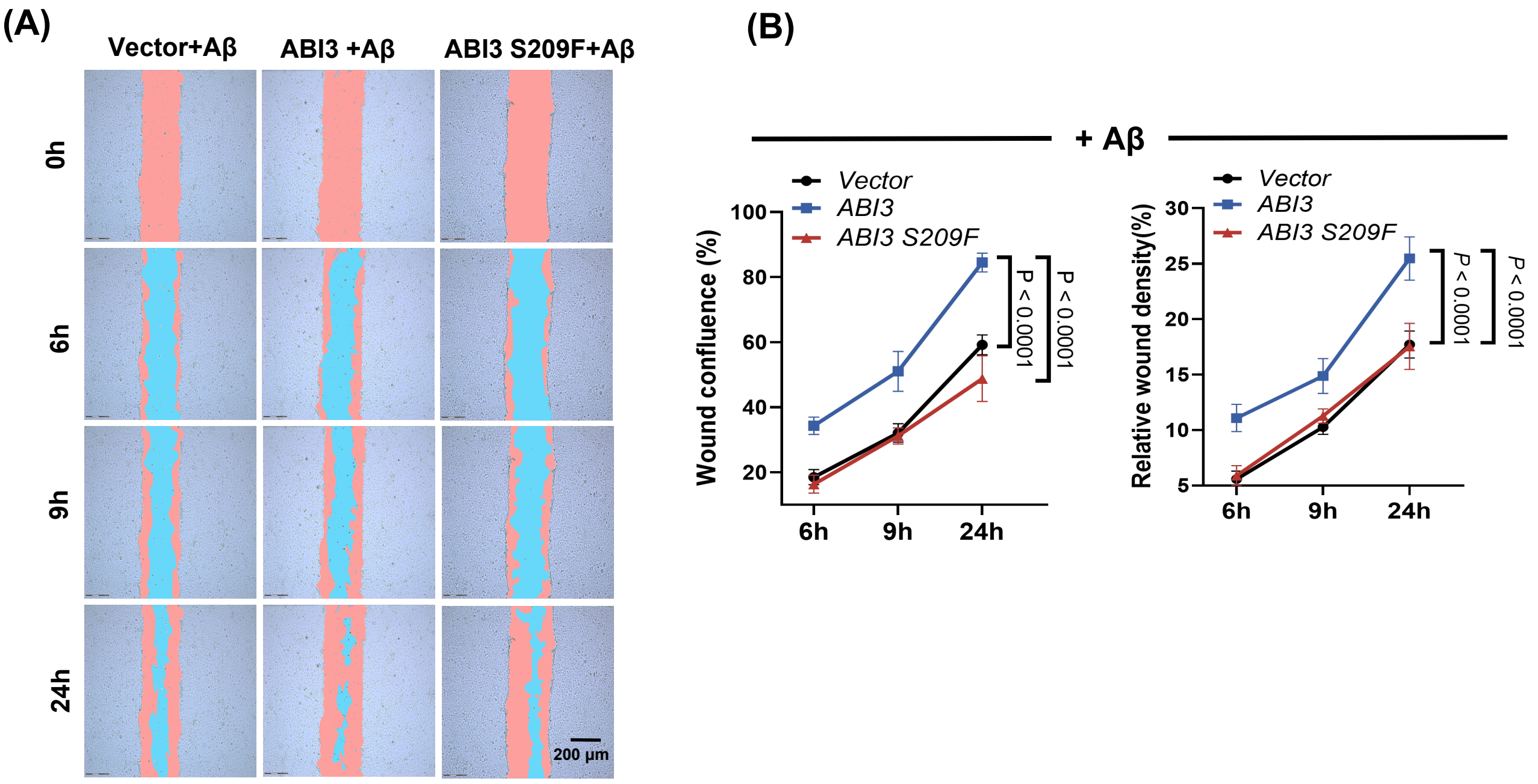

SUPPLEMENTARY FIGURE 7 *S209F* mutation impairs ABI3-enhanced cell migration and phagocytosis.

(A) Representative images of scratch areas in HMC3 cells overexpressing ABI3 or *ABI3 S209F* with 100 nM Aβ stimulation, at 6 h, 12 h and 24 h. Scale bar: 200 μm. **(B)** Statistical analysis of wound healing rates (left) and relative wound density (right) in HMC3 cells overexpressing ABI3 or *ABI3 S209F* with 100 nM Aβ stimulation, at 6 h, 12 h and 24 h. Data is presented as mean±SEM. Statistical analysis was performed using two-way ANOVA **(B)**.
