# Supplementary material for: Aberrant phase separation from a rare ABI3 mutation drives microglial dysfunction and Alzheimer's risk
Source: Alzheimers Dement. 2026 Sep 24;22(9):e71812. doi: 10.1002/alz.71812 (PMC13602175; doi:10.1002/alz.71812)
Supplement: Supplementary file 24 [file ALZ-22-e71812-s024.pdf]

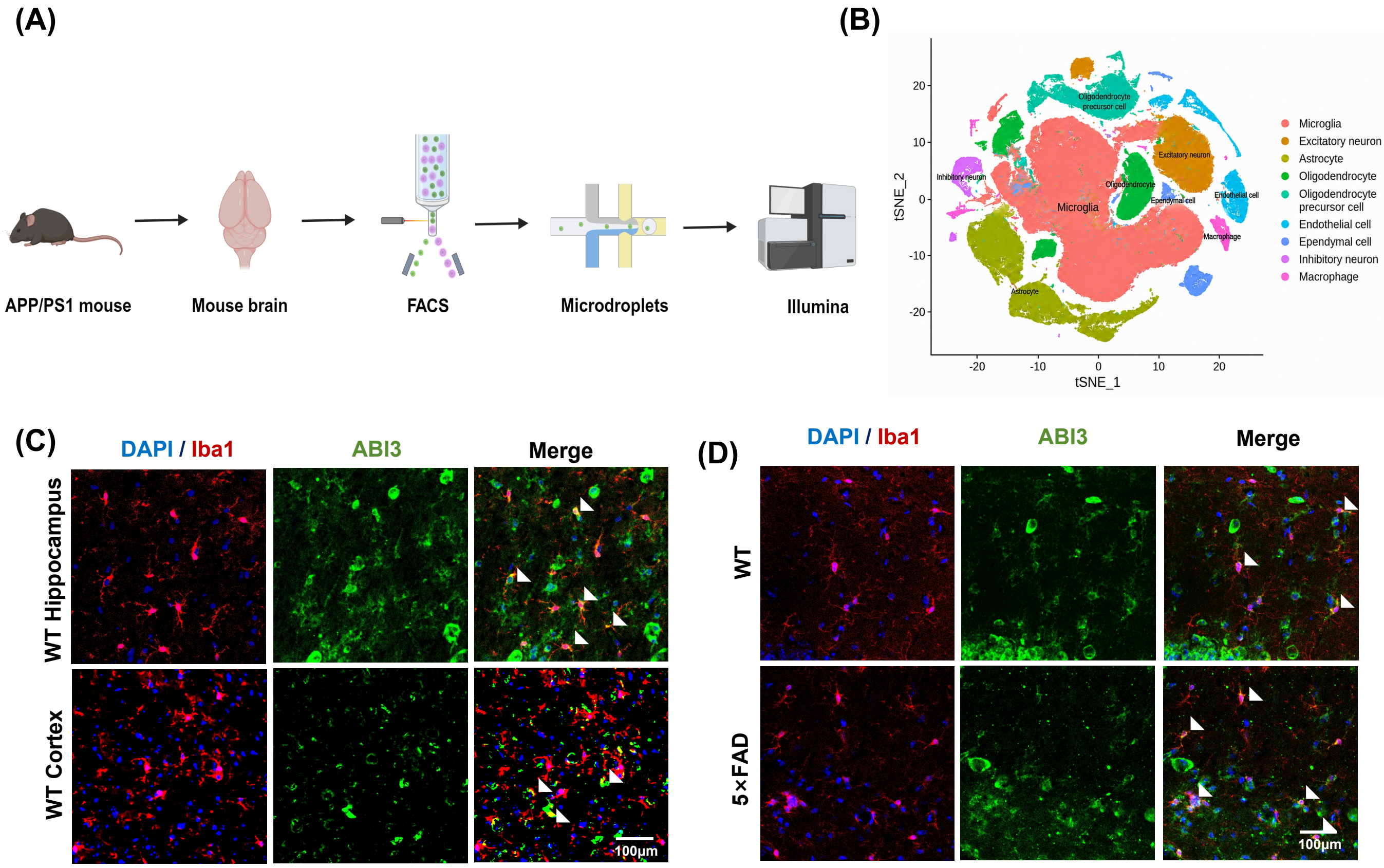

SUPPLEMENTARY FIGURE 1 ABI3 is highly expressed in microglia of APP/PS1 transgenic mice. **(A)** Flowchart of single-cell RNA-seq analysis on brain tissue from APP/PS1 Tg mice. **(B)** Cellular heterogeneity across 55,035 brain cells identified by t-SNE mapping. ScRNA-seq reveals *Abi3* is highly expressed in microglia. (**C**) ABI3 colocalization with microglia in the hippocampus and cortex of 8-month-old C57BL/6 mice. Scale bar: 100 μm. **(D)** ABI3 expression in hippocampal microglia of 8-month-old C57BL/6 mice, with enhanced expression in 5×FAD mice. White arrows indicate Iba1^{+}ABI3^{+} cells. Scale bar: 100 μm.
