# Supplementary material for: Aberrant phase separation from a rare ABI3 mutation drives microglial dysfunction and Alzheimer's risk
Source: Alzheimers Dement. 2026 Sep 24;22(9):e71812. doi: 10.1002/alz.71812 (PMC13602175; doi:10.1002/alz.71812)
Supplement: Supplementary file 25 [file ALZ-22-e71812-s025.pdf]

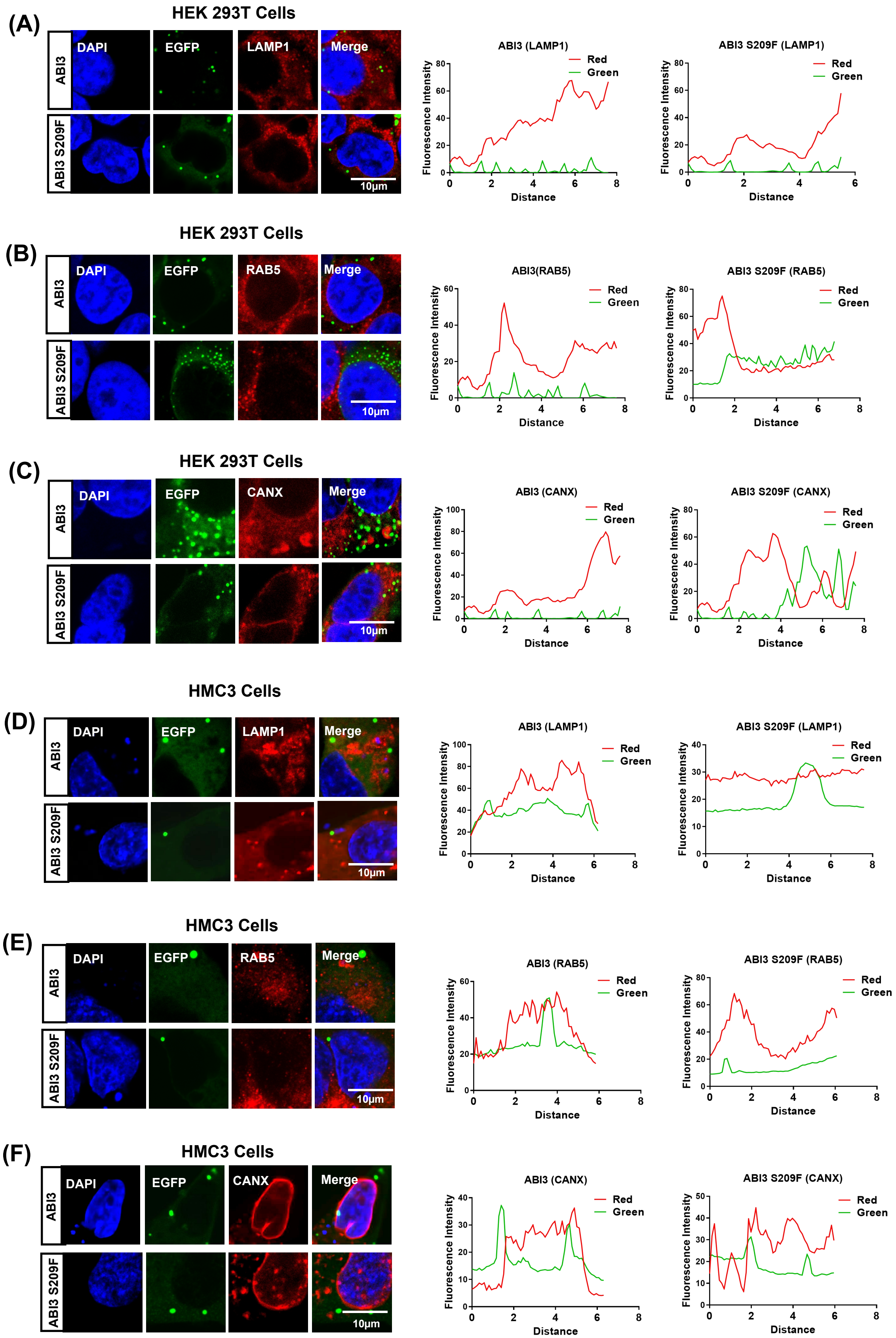
(A)
HEK 293T Cells
DAPI
EGFP
LAMP1
Merge
ABI3
ABI3 S209F
10μm
ABI3 (LAMP1)
ABI3 S209F (LAMP1)
Red
Green
Fluorescence Intensity
Distance
(B)
HEK 293T Cells
DAPI
EGFP
RAB5
Merge
ABI3
ABI3 S209F
10μm
ABI3(RAB5)
ABI3 S209F (RAB5)
Red
Green
Fluorescence Intensity
Distance
(C)
HEK 293T Cells
DAPI
EGFP
CANX
Merge
ABI3
ABI3 S209F
10μm
ABI3 (CANX)
ABI3 S209F (CANX)
Red
Green
Fluorescence Intensity
Distance
(D)
HMC3 Cells
DAPI
EGFP
LAMP1
Merge
ABI3
ABI3 S209F
10μm
ABI3 (LAMP1)
ABI3 S209F (LAMP1)
Red
Green
Fluorescence Intensity
Distance
(E)
HMC3 Cells
DAPI
EGFP
RAB5
Merge
ABI3
ABI3 S209F
10μm
ABI3 (RAB5)
ABI3 S209F (RAB5)
Red
Green
Fluorescence Intensity
Distance
(F)
HMC3 Cells
DAPI
EGFP
CANX
Merge
ABI3
ABI3 S209F
10μm
ABI3 (CANX)
ABI3 S209F (CANX)
Red
Green
Fluorescence Intensity
Distance

SUPPLEMENTARY FIGURE 9 Droplet-like condensates from ABI3 and *ABI3 S209F* overexpression localize outside lysosomes, endosomes, and endoplasmic reticulum in HEK-293T and HMC3 cells.

(A) ABI3-EGFP and *ABI3 S209F*-EGFP droplet-like condensates do not colocalize with lysosomes (LAMP1) in HEK 293T cells. Scale bar: 10 μm. **(B)** ABI3-EGFP and *ABI3 S209F*-EGFP droplet-like condensates do not colocalize with endosomes (RAB5) in HEK 293T cells. Scale bar: 10 μm. **(C)** ABI3-EGFP and *ABI3 S209F*-EGFP droplet-like condensates do not colocalize with the endoplasmic reticulum (CANX) in HEK 293T cells. Scale bar: 10 μm. **(D)** ABI3-EGFP and *ABI3 S209F*-EGFP droplet-like condensates do not colocalize with lysosomes (LAMP1) in HMC3 cells. Scale bar: 10 μm. **(E)** ABI3-EGFP and *ABI3 S209F*-EGFP droplet-like condensates do not colocalize with endosomes (RAB5) in HMC3 cells. Scale bar: 10 μm. **(F)** ABI3-EGFP and *ABI3 S209F*-EGFP droplet-like condensates do not colocalize with the endoplasmic reticulum (CANX) in HMC3 cells. Scale bar: 10 μm.
