# Supplementary material for: Aberrant phase separation from a rare ABI3 mutation drives microglial dysfunction and Alzheimer's risk
Source: Alzheimers Dement. 2026 Sep 24;22(9):e71812. doi: 10.1002/alz.71812 (PMC13602175; doi:10.1002/alz.71812)
Supplement: Supplementary file 27 [file ALZ-22-e71812-s027.pdf]

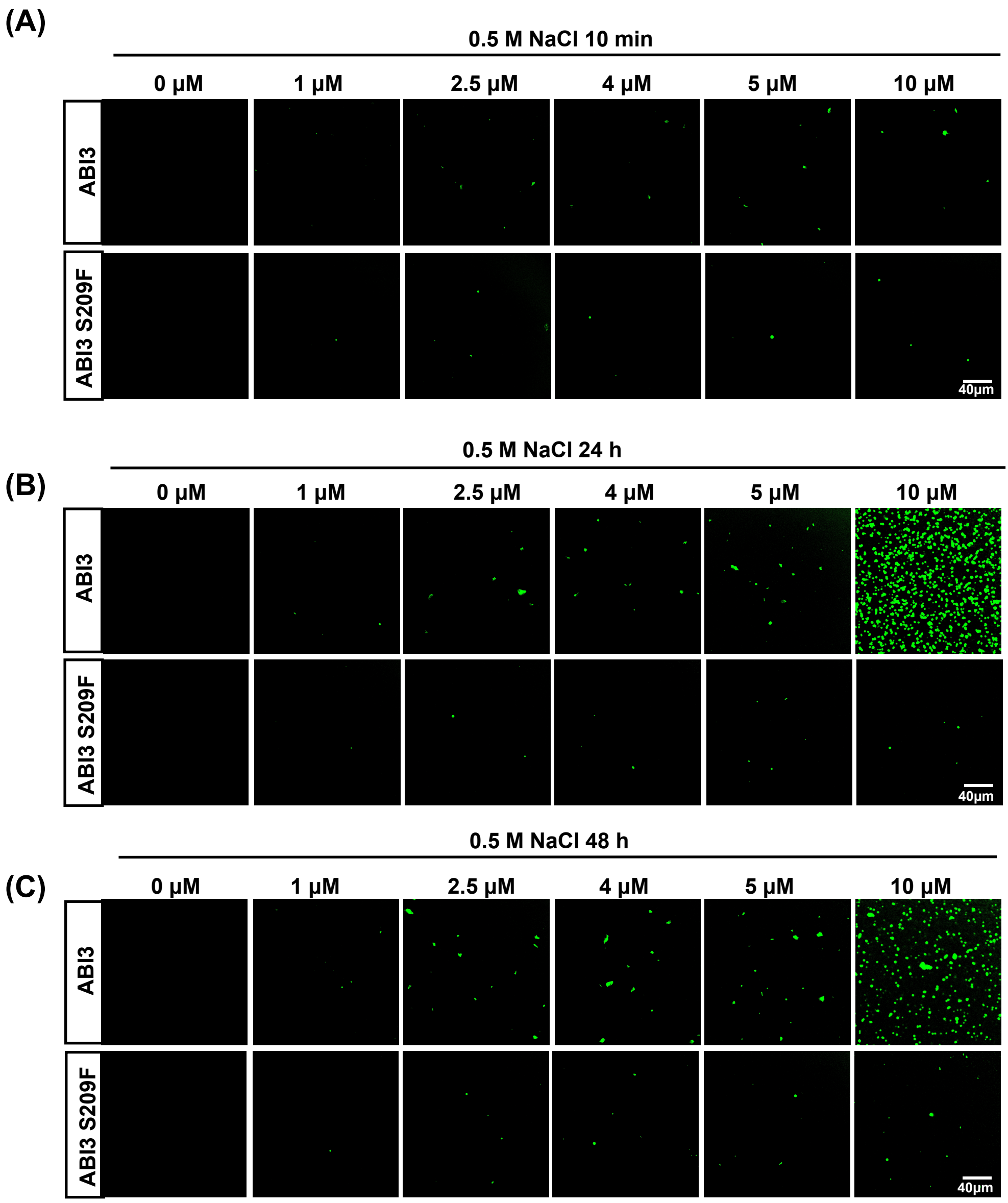

SUPPLEMENTARY FIGURE 17 High-concentration ABI3 forms more and larger droplets over time when mixed with 0.5 M NaCl.

(A) Representative fluorescence images of liquid-liquid phase separation (LLPS) in vitro after mixing different concentrations of ABI3 and *ABI3 S209F* with 0.5 M NaCl for 10 min. Scale bar: 40 μm. **(B)** Representative fluorescence images showing differences in LLPS in vitro after mixing different concentrations of ABI3 and *ABI3 S209F* with 0.5 M NaCl for 24 h. Scale bar: 40 μm. **(C)** Representative fluorescence images showing differences in LLPS in vitro after mixing different concentrations of ABI3 and *ABI3 S209F* with 0.5 M NaCl for 48 h. Scale bar: 40 μm.
