# Supplementary material for: Aberrant phase separation from a rare ABI3 mutation drives microglial dysfunction and Alzheimer's risk
Source: Alzheimers Dement. 2026 Sep 24;22(9):e71812. doi: 10.1002/alz.71812 (PMC13602175; doi:10.1002/alz.71812)
Supplement: Supplementary file 29 [file ALZ-22-e71812-s029.pdf]

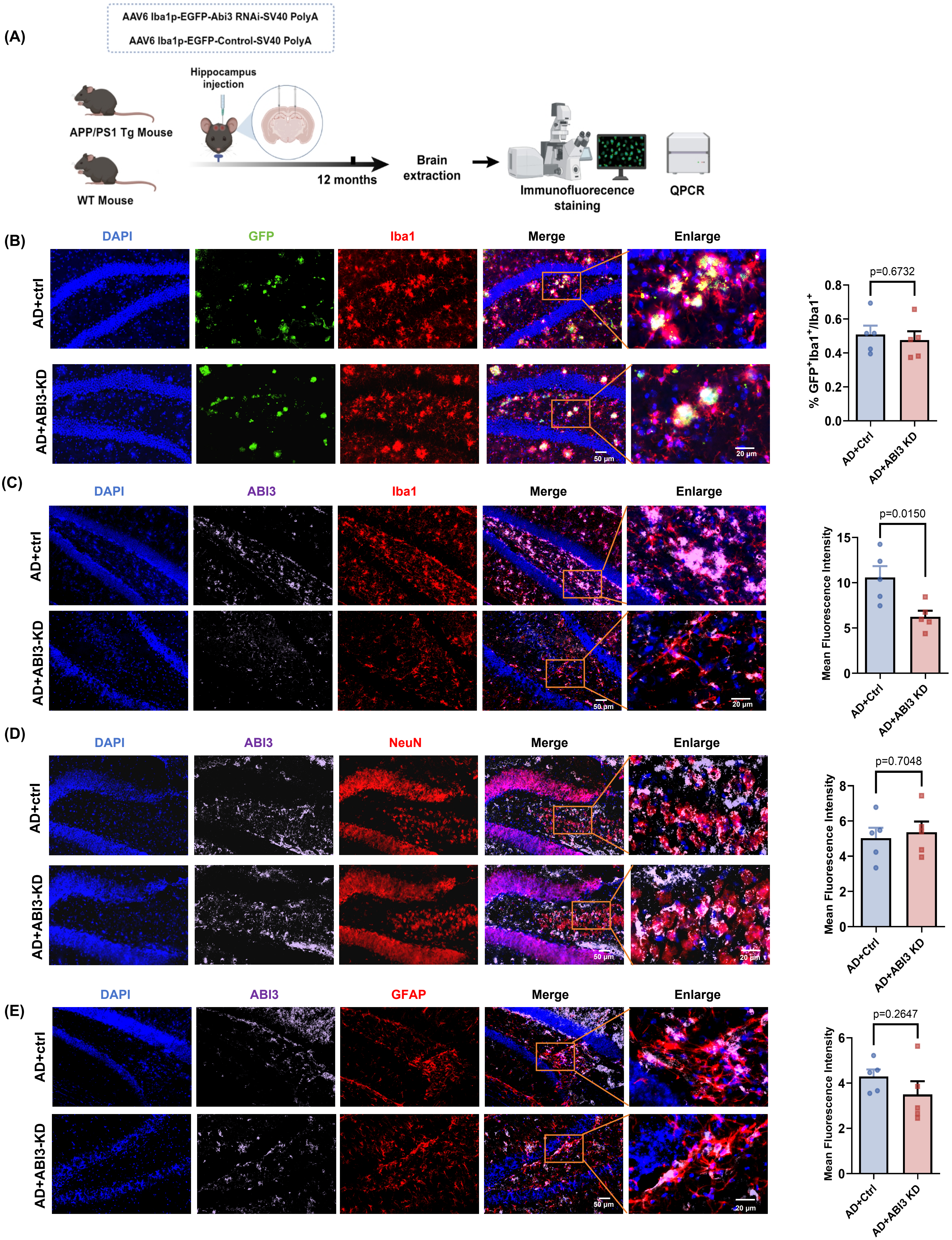

(A)
AAV6 Iba1p-EGFP-Abi3 RNAi-SV40 PolyA
AAV6 Iba1p-EGFP-Control-SV40 PolyA
APP/PS1 Tg Mouse
WT Mouse
Hippocampus injection
12 months
Brain extraction
Immunofluorescence staining
QPCR
(B)
DAPI
GFP
Iba1
Merge
Enlarge
AD+ctrl
AD+ABI3-KD
50 μm
20 μm
p=0.6732
% GFP+Iba1+/Iba1+
AD+Ctrl
AD+ABI3 KD
(C)
DAPI
ABI3
Iba1
Merge
Enlarge
AD+ctrl
AD+ABI3-KD
50 μm
20 μm
p=0.0150
Mean Fluorescence Intensity
AD+Ctrl
AD+ABI3 KD
(D)
DAPI
ABI3
NeuN
Merge
Enlarge
AD+ctrl
AD+ABI3-KD
50 μm
20 μm
p=0.7048
Mean Fluorescence Intensity
AD+Ctrl
AD+ABI3 KD
(E)
DAPI
ABI3
GFAP
Merge
Enlarge
AD+ctrl
AD+ABI3-KD
50 μm
20 μm
p=0.2647
Mean Fluorescence Intensity
AD+Ctrl
AD+ABI3 KD

Supplementary Figure 2 Validation of ABI3 knockdown efficiency and cell-type specificity in the mouse hippocampus.

(A) Timeline of experimental procedures for C57BL/6 and APP/PS1 Tg mice. **(B)** Quantification of transduction efficiency, shown as the percentage of $Iba1^{+}$ microglia that are GFP^{+}. n = 5 mice / group.Scale bar:50 μm, scale bar:20 μm (Enlarge). **(C)** Low magnification whole section immunofluorescence images showing ABI3 (purple), Iba1 (microglia, red), and DAPI (blue) in the hippocampal injection region of control and knockdown mice. Scale bar:50 μm; high magnification insets from the boxed areas in **(C)**, demonstrating single cell level reduction of ABI3 specifically in $Iba1^{+}$ microglia in the knockdown group. Scale bar:20 μm (Enlarge). n = 5 mice / group. **(D)** Cell type specificity analysis: co-staining of ABI3 (purple) with NeuN (neurons, red) in control and knockdown sections. Scale bar:50 μm, scale bar:20 μm (Enlarge). n = 5 mice / group. **(E)** Celltype specificity analysis: co-staining of ABI3 (purple) with GFAP (astrocytes, red) in control and knockdown sections. Scale bar:50 μm, scale bar:20 μm (Enlarge). n = 5 mice / group. ABI3 signal is selectively reduced in $Iba1^{+}$ cells **(C)**, while $NeuN^{+}$ and $GFAP^{+}$ cells show no change **(D-E)**. Data are presented as mean $\pm$ SEM. Statistical analyses were performed using Student's t-test **(B-E)**.
