# Supplementary material for: Aberrant phase separation from a rare ABI3 mutation drives microglial dysfunction and Alzheimer's risk
Source: Alzheimers Dement. 2026 Sep 24;22(9):e71812. doi: 10.1002/alz.71812 (PMC13602175; doi:10.1002/alz.71812)
Supplement: Supplementary file 30 [file ALZ-22-e71812-s030.pdf]

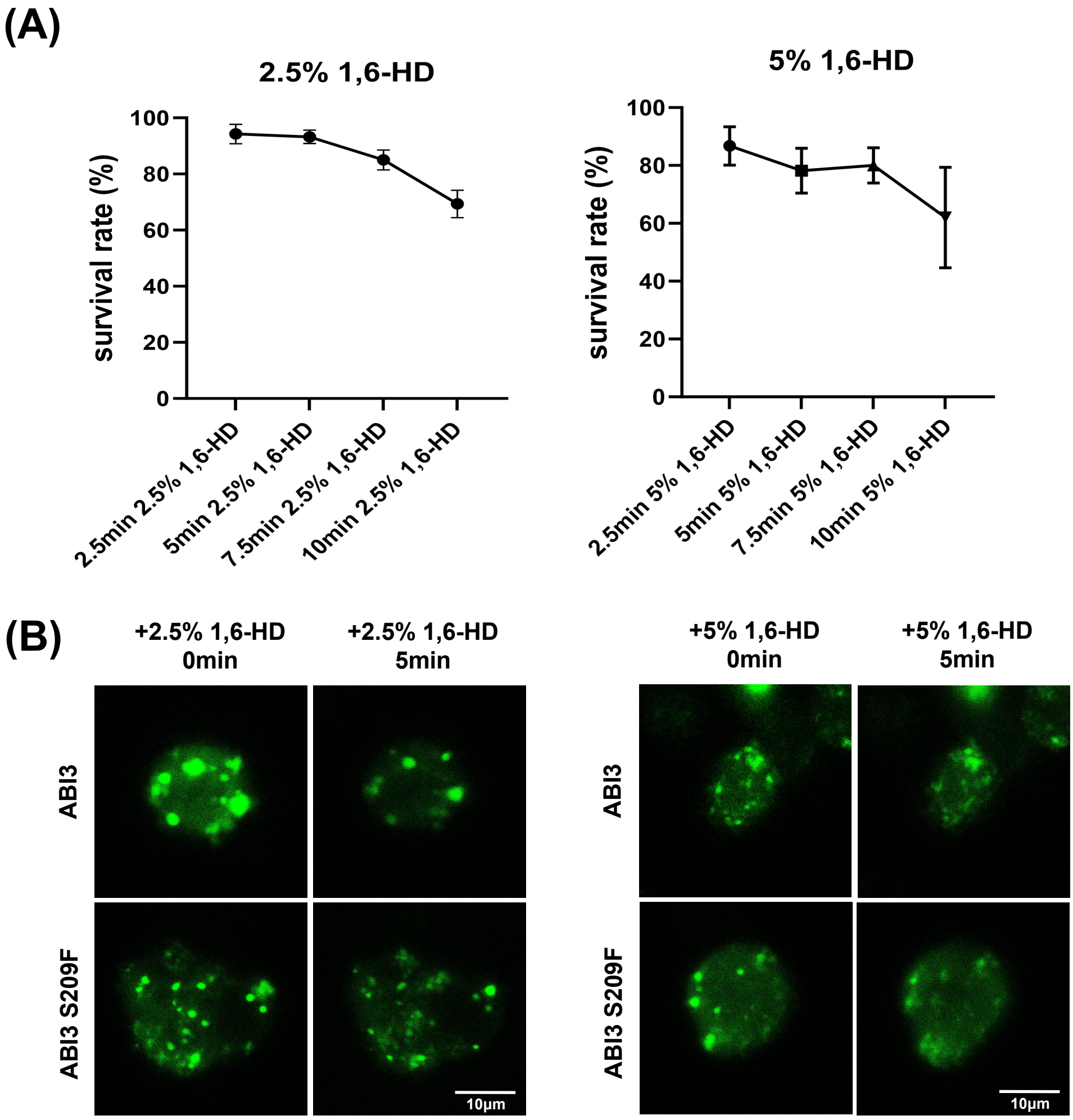

SUPPLEMENTARY FIGURE 10 Non-cytotoxic low concentrations of 1,6-HD disrupt the liquid-liquid phase separation (LLPS) of intracellular ABI3 and *ABI3 S209F*.

(A) CCK-8 assay results showed that low concentrations （2.5% or 5%） of 1,6-hexanediol (1,6-HD) applied for a short duration exhibited no obvious cytotoxicity in 293T cells. **(B)** Both 2.5% and 5% 1,6-HD can disrupt the liquid-liquid phase separation (LLPS) of intracellular ABI3 and *ABI3 S209F* in 293T cells. Scale bar: 10 μm.
